# S···O Conformation Locks Synergistic Alkoxy Chain Engineering of NIR‐II Phototheranostic Molecules for Precision Hepatocellular Carcinoma Theranostics

**DOI:** 10.1002/advs.202506664

**Published:** 2025-11-28

**Authors:** Gui‐long Wu, Fan Wu, Senyou Tan, Hao Xiao, Qiang Kang, Sanlin Deng, Fen liu, Jinkang Zheng, Chaoqiang Li, Guodong Chen, Qinglai Yang

**Affiliations:** ^1^ Department of Hepatopancreatobiliary Surgery The First Affiliated Hospital Hengyang Medical School University of South China Hengyang Hunan 421001 China; ^2^ Center for Molecular Imaging Probe Cancer Research Institute & Hunan Engineering Research Center for Early Diagnosis and Treatment of Liver Cancer & MOE Key Lab of Rare Pediatric Disease & NHC Key Laboratory of Birth Defect Research and Prevention Hengyang Medical School University of South China Hengyang Hunan 421001 China; ^3^ Department of Radiology The Second Affiliated Hospital Hengyang Medical School University of South China Hengyang Hunan 421001 China; ^4^ Department of General Surgery Turpan City People's Hospital Tulufan 838000 China

**Keywords:** hepatocellular carcinoma, NIR‐II phototheranostics, S···O conformation locks, S‐D‐A‐D‐S, theranostics

## Abstract

NIR‐II phototheranostics offers a promising strategy for precisely managing deep‐seated and refractory tumors. However, the overall optimization of molecular functional properties remains a challenge, and due to the lack of a comprehensive design strategy, there have been limitations in achieving long‐wavelength phototherapy, high‐fluorescence quantum yield, and photothermal/photodynamic conversion efficiency. This study initially proposes an innovative strategy that involves S···O confirmation locks (SoCLs), synergistic alkoxy chain engineering, and the regulatory influence of the SoCLs on NIR‐II S‐D‐A‐D‐S‐type (shield‐donor‐acceptor‐donor‐shield) molecular planarity and successfully optimizes the molecular structure. The resulting IR‐BTOG molecules exhibit extended coverage across the NIR‐IIa (λex: 1000–1300 nm) and NIR‐IIb (λem: 1500–1700 nm) regions, achieving high fluorescence quantum yields and significantly improved photothermal performance. Building on this molecular design, BTOGP‐GPC3 nanoparticles (NPs) are further developed by conjugating the hepatocellular carcinoma (HCC)‐specific targeting molecule Glypican‐3 peptide (GPC3). This conjugation enables precise recognition and diagnosis of HCC. The excellent phototheranostic performance of BTOGP‐GPC3 NPs confirms that the SoCLs synergistic alkoxy chain modification strategy markedly enhances the diagnostic performance of the molecule in deep‐seated tumors, offering novel opportunities for applying precision phototheranostics in HCC. Moreover, it provides a significant structural design foundation for the future advancement of NIR‐II phototheranostic formulations.

## Graphical abstract

This study introduces a synergistic S‐O conformation locking and alkoxy chain engineering strategy to enhance S‐D‐A‐D‐S molecular planarity. The engineered IR‐BTOG achieves concurrent NIR‐IIa absorption and NIR‐IIb emission, demonstrating exceptional photothermal efficiency and fluorescence quantum yield. GPC3‐targeted BTOGP‐GPC3 nanoparticles enable precise diagnosis and treatment of deep‐seated hepatic tumors, advancing NIR‐II biomedical applications.

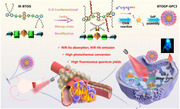

## 1 Introduction

The near‐infrared II (NIR‐II) spectral region (1000–1700 nm) provides advantages for precision medicine applications owing to its superior tissue penetration depth, minimized autofluorescence interference, and high spatial resolution.^[^ ^1^, ^2^, ^3^, ^4^, ^5^ ^]^ These remarkable optical characteristics render NIR‐II‐based phototheranostic molecules well‐suited for addressing challenging clinical scenarios, such as deep‐seated and refractory tumors.^[^ ^2^, ^6^, ^7^ ^]^ Currently, within the realm of precision tumor diagnosis and treatment, the development of comprehensive phototheranostic probes that integrate near‐infrared II (NIR‐II) fluorescence imaging with photothermal therapy (PTT)/photodynamic therapy (PDT) represents a hot point of research. It exhibits exceptional tissue penetration depth, superior photothermal conversion efficiency, and a high fluorescence quantum yield for the incorporated NIR‐II fluorophore. Addressing the fundamental scientific challenge in this field requires devising strategies to effectively mitigate intramolecular energy dissipation while achieving an optimal balance among critical molecular properties, including tissue penetration, photothermal efficiency, and fluorescence quantum yield.^[^ ^8^, ^9^, ^10^, ^11^ ^]^

D‐A‐D (donor‐acceptor‐donor) type of organic phototheranostic molecules have garnered significant attention. This prominence stems from their good biocompatibility within the tested dose range and structural versatility, which allows for precise modulation of fluorescence intensity, emission wavelength, and photothermal properties.^[^ ^3^, ^12^, ^13^ ^]^ Lee et al. demonstrated NIR‐IIb (1500 nm) emission by optimizing fluorine substitution and incorporating selenium into the compound design.^[^ ^14^ ^]^ Fan et al. enhanced molecular planarity and photothermal properties through carbon chain adjustments and introduced bulky groups such as ethylenedioxythiophene (EDOT).^[^ ^15^ ^]^ Tang and Wang et al. employed Noncovalent conformational locks (NoCLs) to regulate intramolecular interactions (e.g., O‐S and N‐S bonding), thereby reinforcing molecular planarity and rigidity.^[^ ^16^ ^]^ Concurrently, integrating aggregation‐induced emission (AIE) principles has proven effective in enhancing fluorescence emission and achieving wavelength red‐shifts.

The strategies mentioned above provide valuable insights and mark a significant advancement in the molecular engineering of NIR‐II molecules. Upon excitation with appropriate wavelengths, NIR‐II molecules undergo electronic transitions from the ground state (S_0_) to the first singlet excited state (S_1_). The absorbed energy can then be dissipated via radiative decay (fluorescence) or, more critically for therapeutic purposes, through non‐radiative pathways that convert the energy into photothermal. This photothermal effect elevates the local temperature, thereby inducing apoptosis in tumor cells. Alternatively, photoexcited electrons may undergo intersystem crossing (ISC) from the singlet to the triplet state (T_1_). The photosensitizer in its excited triplet state can subsequently interact with endogenous biomolecules or molecular oxygen to generate reactive oxygen species (ROS).^[^ ^17^ ^]^ However, the intrinsic competition among fluorescence emission (radiative deactivation), photothermal conversion (non‐radiative relaxation), and photodynamic processes (triplet‐mediated ROS generation) presents formidable challenges for the rational design and controllable optimization of narrow‐bandgap organic chromophores in long‐wavelength NIR‐II applications.^[^ ^11^, ^15^, ^18^, ^19^, ^20^ ^]^ Although existing NIR‐II molecular systems demonstrate notable strengths in individual performance metrics (e.g., extended fluorescence emission wavelengths or high photothermal conversion efficiencies), achieving the holistic optimization necessary for effective integrated theranostics remains a significant challenge.^[^ ^15^, ^21^, ^22^ ^]^ Therefore, a more holistic strategy that considers the comprehensive phototheranostic characteristics of the D‐A‐D NIR‐II molecule needs to be developed. To be specific, for D‐A‐D type NIR‐II molecules, a mature and readily implementable strategy for the simultaneous co‐regulation of excitation (within the 1000‐1300 nm range) and emission (within the 1500–1700 nm range) alongside fluorescence quantum yield (QY) and PTT/PDT efficiency, remains elusive from a molecular design perspective. The comprehensive challenge of tunability presents a significant bottleneck, hindering advancements in NIR‐II phototheranostic performance.

Noncovalent interactions critically influence the physicochemical properties of organic molecules. In particular, the emphasis on intramolecular and intermolecular S···O contacts was established through the seminal analysis of Rosenfield et al.^[^ ^23^ ^]^ Subsequently, a pivotal synthetic strategy has been the strategic employment of noncovalent through‐space intramolecular interactions, commonly known as “noncovalent conformational locks,” to enforce planarity and rigidity upon the conjugated backbone, thereby maximizing π‐system delocalization. This class of interactions includes O···S, N···S, and X···S (where X represents a halide), as well as hydrogen‐bonding motifs.^[^ ^24^ ^]^ Notably, McCullough et al. established a foundational understanding of structure‐property relationships in 1993 by demonstrating the profound influence of molecular conformation on the electrical conductivity of conjugated poly(3‐alkylthiophene) (PAT), marking a seminal contribution to the field of organic semiconductors.^[^ ^25^ ^]^ The “noncovalent conformational lock” paradigm was formally established in 2012 as a rational design strategy for constructing highly planar π‐conjugated architectures, resulting in substantially enhanced charge carrier mobilities in organic field‐effect transistors (OFETs).^[^ ^26^ ^]^ This design principle has subsequently revolutionized the development of planar and rigid conjugated organic semiconductors, fundamentally transforming molecular engineering approaches and significantly advancing performance metrics across a broad spectrum of optoelectronic applications, including organic photovoltaics, light‐emitting diodes, and near‐infrared photodetectors. Within the donor‐acceptor‐donor (D‐A‐D) organic architectural framework for NIR‐II molecular engineering, investigations into the strategic incorporation of S···O noncovalent conformational locks remain notably limited. Although the pivotal role of S···O interactions in modulating molecular planarity and consequent optoelectronic properties has been established, comprehensive mechanistic investigations and systematic structure‐property correlations in this domain are largely underexplored. This knowledge gap presents compelling opportunities to advance fundamental understanding of NIR‐II chromophore design principles and unlock new pathways for molecular optimization in second near‐infrared applications.

**Scheme 1:**
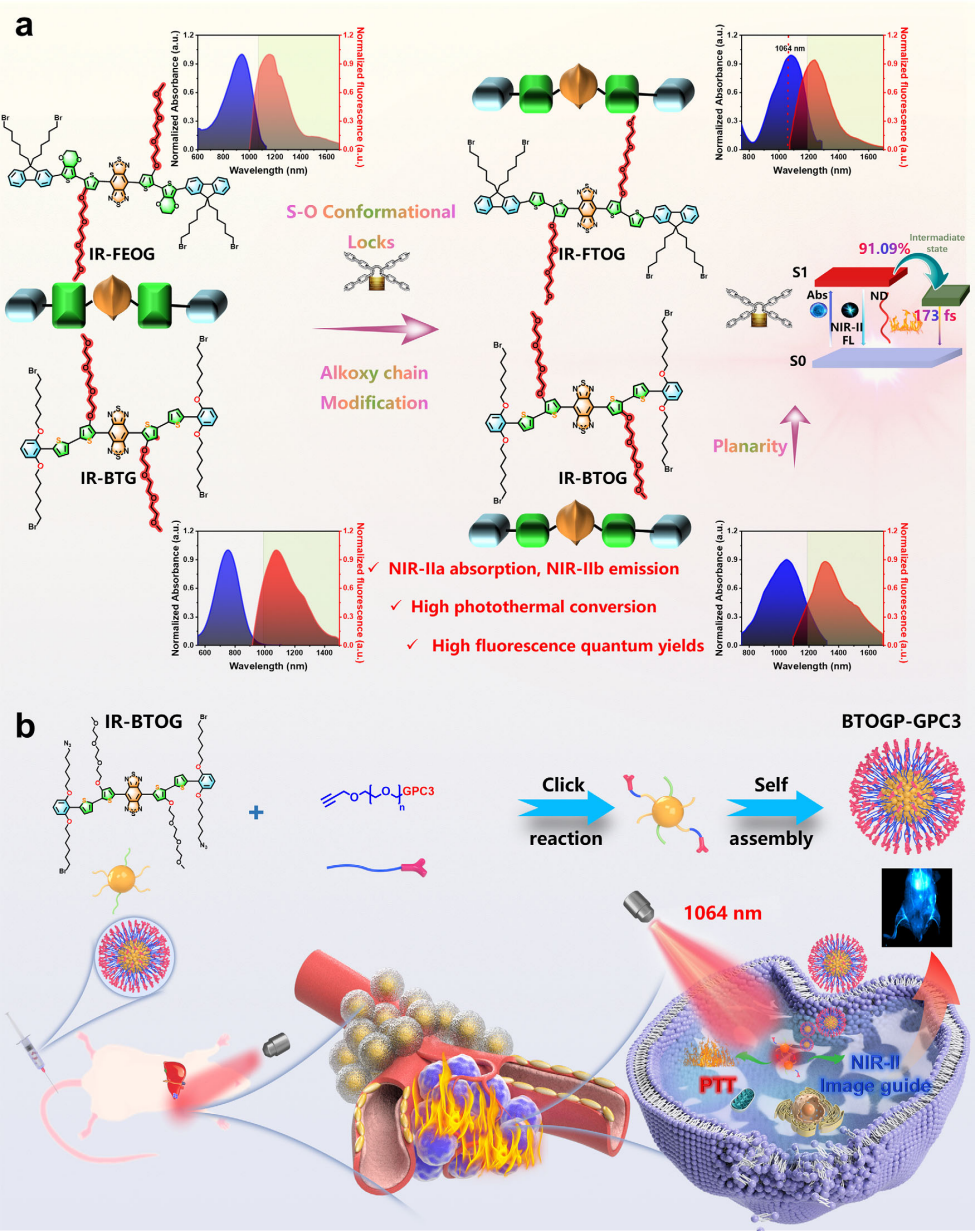
a) SoCLs synergistic alkoxy chain engineering strategy optimized for S‐D‐A‐D‐S type molecular. b) The IR‐BTOG molecule integrated with a GPC3 targeting strategy for the phototheranostics of HCC.

This study initially proposed an original S···O conformation locks (SoCLs) synergistic alkoxy chain engineering strategy applied to the donor unit of an S‐D‐A‐D‐S (shield‐donor‐acceptor‐donor‐shield) type NIR‐II molecule.^[^ ^27^, ^28^, ^29^, ^30^ ^]^ This strategy ultimately developed an S‐D‐A‐D‐S type molecule (IR‐BTOG, where “IR” refers to infrared. The structure incorporates a 2‐bromo‐1,3‐bis((6‐bromohexyl)oxy)benzene shielding unit (“B”), a thiophene donor unit (“T”), and a 3‐(2‐(2‐(2‐methoxyethoxy)ethoxy)ethoxy)thiophene donor unit (“OG”) with high wavelength penetration and excellent photothermal ability (**Scheme** **1**a). This innovative design significantly extends the excitation and emission wavelengths, effectively covering the NIR‐II (λex: 1000–1300 nm) and NIR‐IIb (λem: 1500‐1700 nm) regions. The optimized molecular IR‐BTOGexhibits excellent fluorescence quantum yields (FLQYs) and photothermal conversion efficiency (PCE) at extended wavelengths, providing a robust structural foundation for diagnosis and treatment. To enhance tumor specificity, IR‐BTOG was further optimized by integrating the Glypican 3 peptide (GPC3 peptide)‐targeting strategy, resulting in the formation of BTOGP‐GPC3 nanoparticles (NPs) (Scheme 1b). GPC3 peptide is prominently overexpressed in hepatocellular carcinoma (HCC).^[^ ^31^, ^32^ ^]^ Therefore, BTOGP‐GPC3 NPs hold significant potential for precise and practical phototheranostic applications in HCC. To the best of our knowledge, this study represents the first successful demonstration of a comprehensive optimization strategy for the optical properties of molecules featuring an S‐D‐A‐D‐S configuration. In vitro and in vivo experimental results demonstrate that IR‐BTOG molecules, optimized via the synergistic oxygen‐containing chain length (SoCL) engineering strategy, exhibit a significant red shift in both excitation and emission wavelengths, a substantial enhancement in photothermal conversion efficiency, and a remarkably high fluorescence quantum yield. Furthermore, IR‐BTOG‐modified BTOGP‐GPC3 nanoparticles (NPs) demonstrate pronounced efficacy in both the diagnosis and photothermal therapy of HCC, providing a novel conceptual and structural foundation for the future development of NIR‐II S‐D‐A‐D‐S type molecules.

## 2 Results and Discussion

### 2.1 Molecular Design, Synthesis, and Theoretical Calculation

**Figure 1:**
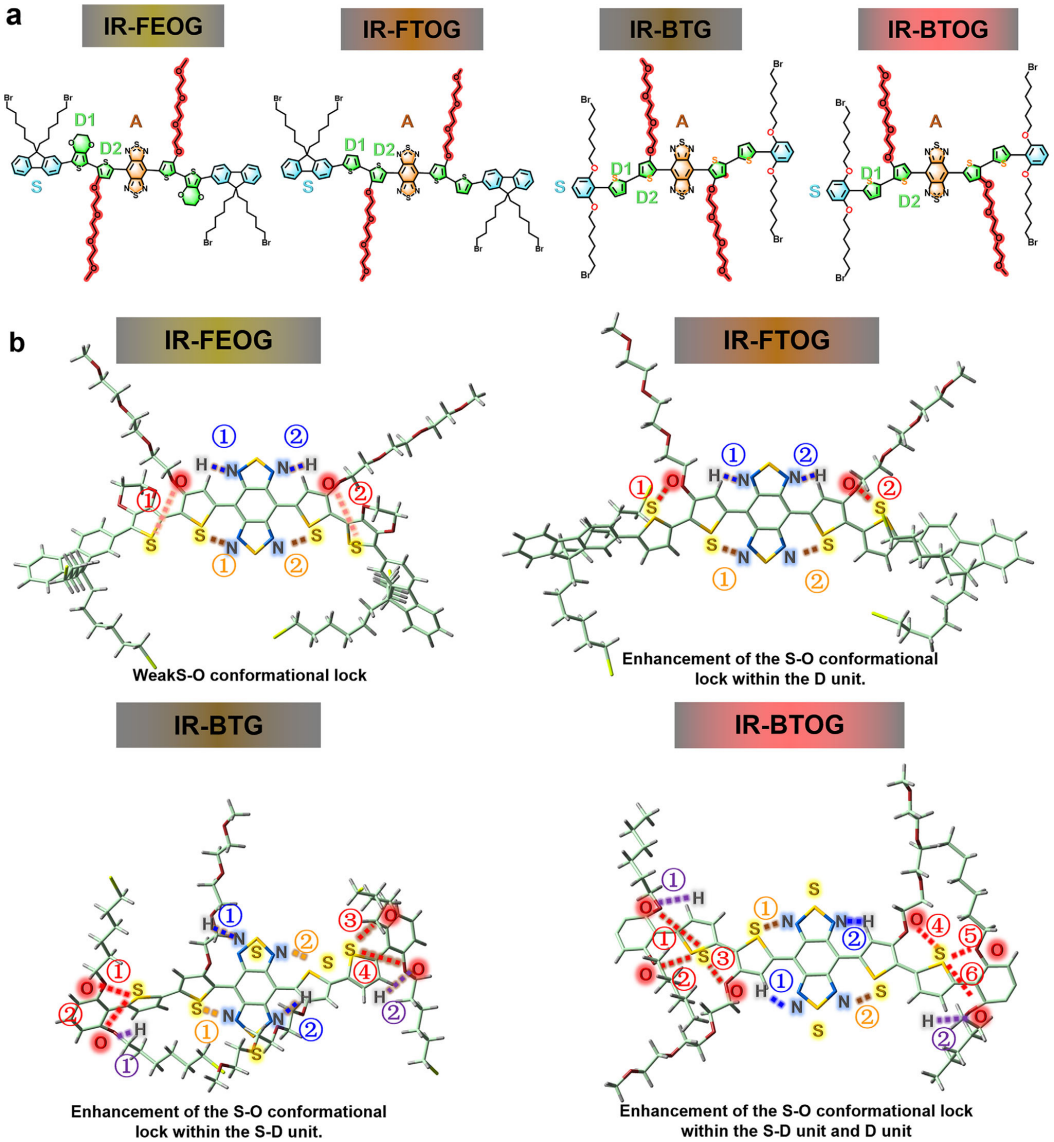
Chemical structures and Molecular calculation of NoCLs. a) Chemical structures of IR‐FEOG, IR‐FTOG, IR‐BTG, and IR‐BTOG, together with the distribution of S (shielding group), D (donor group: including D1 and D2), and A (acceptor group). b) The ball‐and‐stick models of IR‐FEOG, IR‐FTOG, IR‐BTG, and IR‐BTOG serial number, together with their S··O, S··N, N··H, O··H distances and associated ∠C‐S··O ∠C··S··N ∠C··N··H ∠C··O··H angles.

This study, based on the design principles previously reported by our group^[^ ^30^, ^33^, ^34^, ^35^, ^36^ ^]^ (Figure S1, Supporting Information), this investigation systematically integrates the donor (D), 3‐(2‐(2‐(2‐methoxyethoxy)ethoxy)ethoxy)thiophene, and the shielding group (S), 2‐bromo‐1,3‐bis((6‐bromohexyl)oxy)benzene, within a D‐A‐D organic molecular architecture. A series of molecules incorporating both SoCLs and alkoxy chain (IR‐FEOG, IR‐FTOG, IR‐BTG, IR‐BTOG: featuring varied S···O interactions and alkoxy chain between the S and D (D1, D2) groups (**Figure** **1**a) were synthesised to further explore strategies to optimize the optical properties of D‐A‐D‐type NIR‐II fluorophores (Schemes S1–S3 and Figures S2–S28, Supporting Information). This investigation focused on elucidating the impact of subtle structural modifications, specifically the concurrent incorporation of alkoxy chains and SoCLs, on the resultant molecular optical properties. To investigate S···O and alkoxy chain interactions of IR‐FEOG, IR‐FTOG, IR‐BTG, and IR‐BTOG molecules, density functional theory (DFT) calculations were performed employing the B3LYP/6‐311+G (d,p) level of theory (Table S1, Supporting Information). To ensure the accuracy of the geometry optimization and potential energy surface (PES) calculations, PES scans were conducted using the Gaussian program, with a memory allocation of 20 GB and 40 parallel computation cores. Geometry optimization was performed using the parameters Opt (ModRedundant, Calcfc, Maxcycles = 200), incorporating restricted scans for specific dihedral angles, with geometry optimization applied at each scan point. The integration precision was set to the UltraFine grid, and molecular symmetry was turned off (NoSymm), while the SCF convergence criterion was set to SCF = Tight. All calculations successfully converged within the specified iteration limits. Comprehensive PES scans were executed for all rotatable single bonds in IR‐FEQS, IR‐FTQG, IR‐BTG, and IR‐BTOG, with the optimized minimum energy values for each dihedral angle presented below. These calculations enabled the identification of the most stable configurations (Figure S29, Supporting Information). Based on all computational data, this study selectively analyzed regions with strong intermolecular noncovalent interactions, as presented in Figure 1 and **Table** **1** of the main text. Data related to weak interactions can be found in Supporting Information Figure S30 (Supporting Information).

**Table 1:** Detailed data of the various noncovalent conformational locks shown in Figure 1b.

		Noncovalent conformational locks			
Compound	Serial Number	S•••O	S•••N	N•••H	O•••H
IR‐FEOG	①	4.44 Å, 107.59 °	2.78 Å, 95.78 °	2.22 Å, 103.82 °	–
	②	4.73 Å, 92.0 °	2.80 Å, 94.89 °	2.17 Å, 103.19 °	–
IR‐FTOG	①	2.93 Å, 146.4 °	2.76, 165.00 °	2.17 Å, 119.8 °	–
	②	2.89 Å, 113.77 °	2.79 Å, 164.84 °	2.18 Å, 119.23 °	–
IR‐BTG	①	3. 01 Å, 141.18 °	2.91 Å, 139.67 °	2.79 Å, 115.95 °	2.19 Å, 108.2 °
	②	3.20 Å, 91.67 °	3.12 Å, 152.76 °	2.77Å, 99.49 °	2.25 Å, 107.5 °
	③	2.490 Å, 153.15 °	–	–	–
	④	2.820 Å, 94.82 °	–	–	–
IR‐BTOG	①	2.73 Å, 170.63 °	2.79 Å, 154.29 °	2.16 Å, 120.39°	1.83 Å, 123.18 °
	②	3.13 Å, 94.53 °	2.77 Å, 164.67°	2.17 Å, 120.54 °	1.86 Å, 131.30 °
	③	3.06 Å, 144.90 °	–	–	–
	④	3.01 Å, 140.02 °	–	–	–
	⑤	2.54 Å, 137.16 °	–	–	–
	⑥	3.01 Å, 97.95 °	–	–	–

As a control lacking SoCLs, the IR‐FEOG molecule exhibits distinct structural parameters. The S···N and N···H interactions between its A acceptor and D2 donor were found to be S···N (d = 2.78 Å to 2.80 Å) and ∠C‐S···N (94.89° to 95.78°), and N···H (d = 2.17 Å, 2.22 Å) with an ∠C‐H···N ranging from 103.82° to 103.19°. These results indicate strong S···N and N···H interactions between the A acceptor and D2 donor in IR‐FEOG. However, the S···O interaction within the molecule is relatively weak. Within the D1 and D2 regions, the S···O interatomic distance (*d*) ranges from 4.44 Å to 4.73 Å, and the ∠C‐O···S angle measures between 92.0° and 107.59° (The serial Number of S···O SoCLs within the IR‐FEOG: ① and ②, Figure 1b, Table 1). The results above indicate a significant distortion in the ∠C‐O…S angle. Furthermore, the observed S···O interatomic distance substantially exceeds the sum of the van der Waals radii for sulfur and oxygen (drw, S···O distance = 3.25 Å^[^ ^37^ ^]^). This deviation suggests the absence of effective S···O chalcogen bonding interactions within the IR‐FEOG molecules.

**Figure 2:**
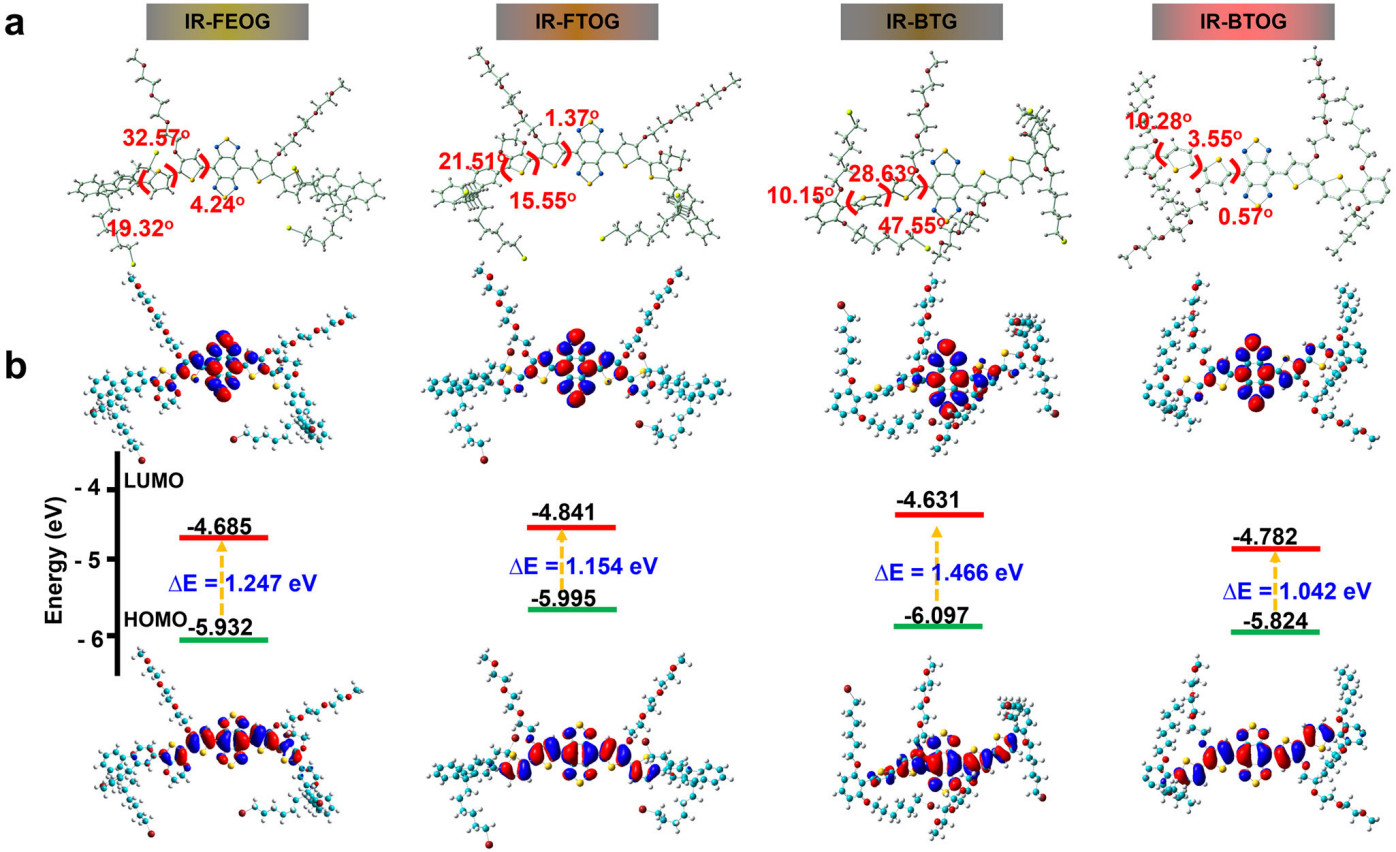
Molecular calculation of dihedral angle, andthe HOMOs and LUMOs energy levels of IR‐FEOG, IR‐FTOG, IR‐BTG, and IR‐BTOG. a) The dihedral angles S‐D1, D1‐D2, and D2‐A of the S0 optimum geometry for IR‐FEOG, IR‐FTOG, IR‐BTG, and IR‐BTOG are calculated employing the DFT approach at the B3LYP/6‐311+G (d,p) level. b) The frontier molecular orbital distributions and energy levels of IR‐FEOG, IR‐FTOG, IR‐BTG, and IR‐BTOG have been calculated employing the B3LYP/6‐311+G (d,p) level by density functional theory (DFT) methodology.

Similarly, the IR‐FTOG molecule exhibits distinct structural parameters. The S···N and N···H interactions between its A acceptor and D2 donor were found to be S···N (d = 2.76 Å to 2.79 Å) and ∠C‐S···N (164.84° to 165.00°), and N···H (d = 2.17 Å to 2.18 Å) with an ∠C‐H···N ranging from 119.23° to 119.23°. These results indicate strong S···N and N···H interactions between the A acceptor and D2 donor in IR‐FTOG. In contrast, although the D2‐A of IR‐FTOG molecule demonstrates negligible intramolecular SoCLs, the SoCLs between the D1 and D2 presents S···O distance = 2.89–2.93 Å, with ∠C‐O…S angles of 113.77°–146.40° (the serial Number of S···O SoCLs within the IR‐FTOG: ① and ②, Figure 1b, Table 1), indicating that the S···O distance is below 3.25 Å and the planarity is enhanced. As a result, the outcomes of the molecular dihedral angle calculation (IR‐FEOG: S‐D1 = 19.32°, D1‐D2 = 32.57°, D2‐A = 4.24°; IR‐FTOG: S‐D1 = 21.51°, D1‐D2 = 15.55°, D2‐A = 1.37°) indicate that the deflection of the dihedral angle diminishes in the D1‐D2 and D2‐A segments. The overall molecular core deflection correspondingly decreased, and planar configuration improved, affording a HOMO‐LUMO bandgap of 1.154 eV (lower than 1.247 eV for IR‐FEOG) (**Figure** **2**a,b). These results indicate that the introduction of 3‐(2‐(2‐(2‐methoxyethoxy)ethoxy)ethoxy)thiophene as D2 indeed enhances the planar configuration of adjacent unit structures throughthe S···O effect, dramatically reduces the bandgap energy, and facilitates wavelength red‐shift. Simultaneously, it is determined that the adjustment of the S···O conformation of the D donor can significantly influence molecular planarity and band gap energy.

The optimized molecular design of IR‐FTOG enhances SoCLs in the D1‐D2 connection. Nevertheless, the planarity of S‐D and D‐A is hardly affected by this configuration. Based on this concept, IR‐BTG was further designed to amplify the influence of SoCLs on the molecular framework of S‐D and D‐A. Similarly, the IR‐BTG molecule exhibits distinct structural parameters. The S···N and N···H interactions between its A acceptor and D2 donor were found to be S···N (d = 2.91 to 3.12 Å) and ∠C‐N···S (139.67° to 152.76°), and N···H (d = 2.77 to 2.79 Å) with an ∠C‐H···N ranging from 99.49° to 115.95°. These results indicate strong S···N and N···H interactions between the A acceptor and D2 donor in IR‐BTG. The design of IR‐BTG resulted in a notable enhancement of the S···O distance (d = 2.49–3.20 Å) and bond angle ∠C‐O…S = 91.67–153.15° (The serial Number of S···O SoCLs within the IR‐BTG: **①**, **②**, **③**, and **④**, Figure 1b, Table 1), accomplished by optimizing the S···O conformation inside the S‐D1unit. This alteration significantly enhances the coplanarity of S‐D1; Meanwhile, the O···H distance (d = 2.19–2.25 Å) and bond angle ∠C‐O…H = 107.5–108.2° (serial numbers of O···H SoCLs within IR‐BTG: ① and ②, Figure 1b, Table 1) were optimized within the S‐D1 unit. This O···H Noncovalent interaction further enhances the coplanarity of S‐D1. However, the spatial arrangement deviation of the alkoxy chain results in an increased S···O distance in the D1‐D2 unit, measuring d = 5.61–6.36 Å (The serial Number of S···O SoCLs within the IR‐BTG: **⑤** and **⑧**, Figure S30, Supporting Information), thereby diminishing the effectiveness of the SoCL in this region. The S···O distance between the D2 and A is d = 4.19–4.34Å (The serial Number of S···O SoCLs within the IR‐BTG: **⑥** and **⑦**, Figure S30, Supporting Information), which notably exceeds the sum of the van der Waals radii (S···O, 3.25Å). Molecular dihedral angle calculations revealed that IR‐BTG exhibited dihedral angles of S‐D1 = 10.15 °, D1‐D2 = 28.63 °, and D2‐A = 47.55 °. In contrast to IR‐FTOG and IR‐FEOG, the sterically demanding long alkoxy chain in IR‐BTG is positioned closer to the acceptor (A) unit. This spatial arrangement induces a pronounced increase in the torsional distortion between the donor (D) and acceptor (A) units. Furthermore, this effect, combined with the weaker intramolecular S···O interaction between the D and A units, contributes to a significantly larger overall molecular dihedral angle in IR‐BTG. The HOMO‐LUMO band gap of the associated IR‐BTG is markedly elevated by 1.466 eV (Figure 2a,b). The results above indicate that the IR‐BTG design is unsatisfactory, suggesting that SoCLs in S (shield) ‐D (donor) are weaker than SoCLs in D to contribute to the entire molecular plane. Yet, it provides an opportunity for further optimization.

Based on the molecular design strategies outlined above, the IR‐BTOG molecule exhibits distinct structural parameters. The S···N and N···H interactions between its A acceptor and D2 donor were found to be S···N (d = 2.77–2.79 Å) and ∠C‐S···N (154.29° to 164.67°), and N···H (d = 2.16–2.17 Å) and ∠C‐H···N (120.39° to 120.54°). These results indicate strong S···N and N···H interactions between the A acceptor and D2 donor in IR‐BTOG. Building upon the experience gained from the above molecular design strategies, the IR‐BTOG molecule, designed after further optimization, provides an accurate design for achieving the optimal SoCLs within the S‐D‐A‐D‐S configuration. In the design of IR‐BTOG, the shielding effect of the S‐D is enhanced by altering the alkoxy chain distal to the donor side of A, creating a stable SoCLs structure. The critical parameters of the conformational lock between the S‐D and D1‐D2 internal S···O are d = 2.54–3.13 Å and ∠C‐O…S = 94.53° to 170.63° (The serial Number of S···O SoCLs within the IR‐BTOG: **①**, **②**, **③**, **④**, **⑤**, and **⑥**, Figure 1b, Table 1); Meanwhile, the O···H distance (d = 1.83–1.86 Å) and bond angle ∠C‐H…O = 123.18° to 131.30° (serial numbers of O···H SoCLs within IR‐BTG: ① and ②, Figure 1b, Table 1) were optimized within the S‐D1 unit. This O···H Noncovalent interaction further enhances the coplanarity of S‐D1. This design markedly enhances the molecular planarity of the S‐D segment of the core (S‐D1 dihedral angle = 10.28°, D1‐D2 dihedral angle = 3.55°). Despite the D2‐A S···O interaction exhibiting a distance (d) of 5.44–5.54 Å and ∠C‐O···S angles ranging from 62.97° to 64.06° (The serial number of S···O SoCLs within the IR‐BTOG: **⑦** and **⑧**, Figure S30, Supporting Information), the spatial separation of the extended alkoxy chain from unit A significantly mitigates steric hindrance. Consequently, a dihedral angle of 0.57° is observed in the D‐A segment, indicating a substantially reduced overall molecular core deflection compared to IR‐FTOG, IR‐FEOG, and IR‐BTG. The HOMO‐LUMO band gap of the relevant IR‐BTOG is markedly reduced to 1.042 eV (Figure 2a,b).

In contrast to conventional geometric analyses relying on traditional parameters such as S···O distances, bond angles, and standard DFT calculations, Liu et al. developed a sophisticated multi‐dimensional metric descriptor *S* to evaluate the degree of noncovalent conformational locking quantitatively. They explicitly reveal a positive correlation between the descriptor *S* and the strength of NoCLs, defined as *S* = (− cos α)•cos ^2^θ(1 − e^Δ*d*^)^2^ (Figure S31, Supporting Information). This is demonstrated through trends such as S···O < Se···O < Te···O and S···F < Se···F < Te···F NoCLs.^[^ ^38^ ^]^ The strength of these NoCLs has a positive association with the value of the *S* descriptor. Building upon Liu et al.'s established methodology, this investigation systematically calculates the *S* descriptor values for S···O conformational interactions within four molecular systems (IR‐BTOG, IR‐FEOG, IR‐FTOG, and IR‐BTG) to elucidate the gradation of interaction strength through multi‐dimensional analysis. As detailed in Table S2 (Supporting Information), the calculated results for S···O, S···N, N···H, O···H interactions, along with bond lengths and bond angles, corroborate the *S* descriptor values in the molecules IR‐BTOG, IR‐FEOG, IR‐FTOG, and IR‐BTG. It is evident that, both in terms of quantity and magnitude, the S descriptor values for IR‐BTOG are significantly higher than those for IR‐FEOG, IR‐FTOG, and IR‐BTG. This finding is consistent with previously established concepts in the literature, where the strength of noncovalent conformational locks (NoCLs) is directly correlated with the *S* descriptor values.

Based on the above results, it can be concluded that the presence of S···N and N···H interactions in the cores of IR‐FEOG, IR‐FTOG, and IR‐BTOG results in relatively unchanged overall planarity in the D2‐A region, leading to high structural rigidity. In IR‐FEOG, the weak S···O interactions within the S‐D and D regions reduce the planarity of the core. In comparison, IR‐FTOG exhibits stronger S···O interactions in the D region, which enhance the rigidity of that region. IR‐BTG, through the S···O interaction at the S‐D end, enhances the planarity of this segment, but its noncovalent conformational lock in the D region is weaker, leading to increased torsion. Furthermore, the spatial steric hindrance effect of the alkoxy side chains in IR‐BTG causes significant twisting in the D2‐A region, resulting in distortion of the core. IR‐BTOG combines the advantages of the previous molecules, with strong S···N and N···H interactions in the D2‐A region, and almost no steric hindrance effect from the alkoxy side chains, resulting in excellent core planarity. This study concludes that NoCLs significantly enhance the planarity of molecules by systematically restricting their rotational degrees of freedom. Within S‐D‐A‐D‐S molecular architectures, the modulatory effects of SoCLs on molecular planarity and bandgap engineering follow a hierarchical order: internal D > S‐D > D‐A interactions. This structure‐property relationship establishes a novel theoretical foundation for the molecular design of NIR‐II materials.

**Figure 3:**
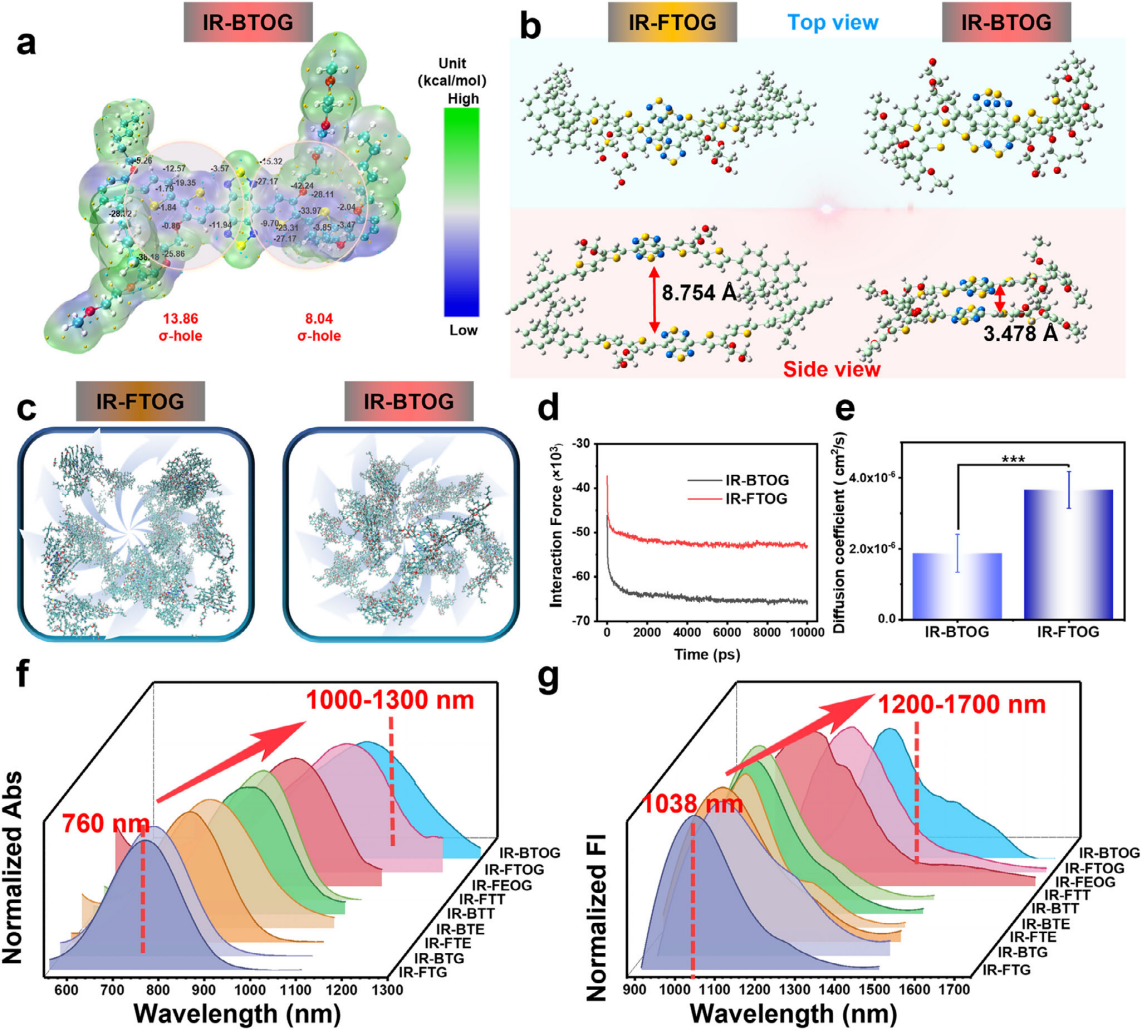
ESP analysis, molecular stacking dynamics modeling, and spectrum characteristics of various molecules. a) ESP analysis is performed with the Multiwfn program and subsequently visualized with the VMD application. The σ‐porosity of the sulfide bond is 14.56 and 12.13 kcal mol^−1^. b) Molecular arrangement and interspacing of IR‐BTOG and IR‐FTOG. c) Molecular dynamics simulations of IR‐FTOG and IR‐BTOG. d) The interaction force between IR‐FTOG and IR‐BTOG. e) Diffusion coefficients of IR‐FTOG and IR‐BTOG. f) Absorption wavelengths and g) emission wavelengths of various D‐A‐D type NIR‐II molecules in DCM.

The classical investigation outlines noncovalent but highly directed intramolecular interactions between sulfur and oxygen atoms, which can also be estimated from the electrostatic potential (ESP) of the outer surface of sulfur atoms.^[^ ^16^, ^39^ ^]^ As shown in **Figure** **3**a and Table S3 (Supporting Information), computed via the Multiwfn program and visualized using the VMD program, the σ hole potential of sulfur (positioned along the extension of the O‐S covalent bond) was found to be + 8.04 and + 13.86 kcal mol^−1^, respectively. The results demonstrate that the σ hole of the associated sulfur in the molecule encounters a significant slump, reflecting a substantial change in electric potential from positive to negative (‐1.79 to ‐42.4 kcal mol^−1^). This result can be attributed to the electrostatic attraction between the negatively potential oxygen atoms in the alkoxy chain and the positively potential sulfur atoms, forming multiple SoCLs within the intramolecular main chain. This mechanism significantly enhances the molecular planarity on both sides of the molecule and substantially restricts intramolecular motion. Furthermore, as shown in Figure 3b, the stacking distance of IR‐FTOG (8.754 Å) goes above that of IR‐BTOG (3.478 Å), thus demonstrating that the enhanced planarity of the SoCLs relatively elevates the π‐π stacking potential of the molecules.

The results mentioned above are consistent with the energy band data (Figure 1d; Table S4, Supporting Information). The strategic incorporation of multiple SoCLs structures within the molecular framework significantly enhances molecular rigidity, affirms the molar extinction coefficient, and strengthens the donor‐acceptor (D‐A) interaction, leading to redshifted absorption and emission spectra, thereby collectively boosting its efficacy in phototheranostic applications.

Moreover, molecular dynamics (MD) simulations were conducted to examine the intermolecular interactions of the designed NIR‐II fluorophores(100 monomers of each aggregate to mimic their behavior in real systems) (Supplementary Information for computational details). As illustrated in Figure 3C, the molecules evolve from a monodisperse state into loosely associated dimers or trimers, with no significant formation of large aggregates.  Simultaneously, the motion trajectories of IR‐BTOG and IR‐FTOG molecules inside a limited region were recorded from 0 to 200 000 ps. The linear simulation results indicated that the molecular force stabilized at 65 826.3 kJ mol^−1^ for IR‐BTOG and 53 029.5 kJ mol^−1^ for IR‐FTOG (Figure 3d; Figure S32, Supporting Information). In addition, the corresponding diffusion coefficients IR‐BTOG and IR‐FTOG are 0.187 × 10^−5^ and 0.366 × 10^−5^ cm^2^ s^−1^ (Figure 3e). The MD calculations results indicated that IR‐BTOG and IR‐FTOG preserve distinct intermolecular interactions owing to the integration of SOCLs and alkoxy chains. The extended alkoxy chains in both the IR‐BTOG and IR‐FTOG compounds enhance molecular dispersion. However, due to distinct intermolecular interactions, IR‐BTOG demonstrates a lower degree of molecular dispersion relative to IR‐FTOG.

As shown in Figure 3f,g, Figure S1, and Table S5 (Supporting Information),^[^ ^30^, ^33^, ^34^, ^35^, ^36^ ^]^ our group's previous investigation has demonstrated that S and D unit optimization significantly influences the molecule's essential optical characteristics, consequently enhancing the excitation and emission wavelengths to IR‐FTT (λex = 890 nm, λem = 1112 nm). In this study, through the optimization strategy of SoCLs synergistic alkyl oxygen chain engineering, the newly developed IR‐FEOG, IR‐FTOG, and IR‐BTOG fluorophores exhibit significant red shifts in absorption and emission spectra. The absorption peaks of IR‐FEOG, IR‐FTOG, and IR‐BTOG in dichloromethane (DCM) are located at 944, 1032, and 1051 nm, respectively. Their corresponding emission maxima were recorded at 1140, 1242, and 1303 nm, respectively. The fluorescence quantum yields (QYs) of fluorophores in dichloromethane were calculated to be 0.0596% (IR‐FTT), 0.1353% (IR‐FEOG), 0.1731% (IR‐FTOG), and 0.0956% (IR‐BTOG), respectively.

The calculations above indicate that, during molecular optimization, the HOMO‐LUMO energy gap minimizes due to the planar effect of S···O, thereby enhancing the potential for electron transfer from the ground state S_0_ to the excited state S_1_ (S_0_‐S_1_ absorption). The absorption and fluorescence of IR‐BTG, IR‐FEOG, IR‐FTOG, and IR‐BTOG molecules exhibit a progressive red shift. The substantial steric hindrance conferred by these molecular architectures enables IR‐FEOG, IR‐FTOG, and IR‐BTOG to maintain well‐preserved quantum yields relative to the IR‐26 reference standard, thereby demonstrating promising potential for photodynamic diagnosis and therapeutic applications.

### 2.2 Photothermal Mechanism Studies

**Figure 4:**
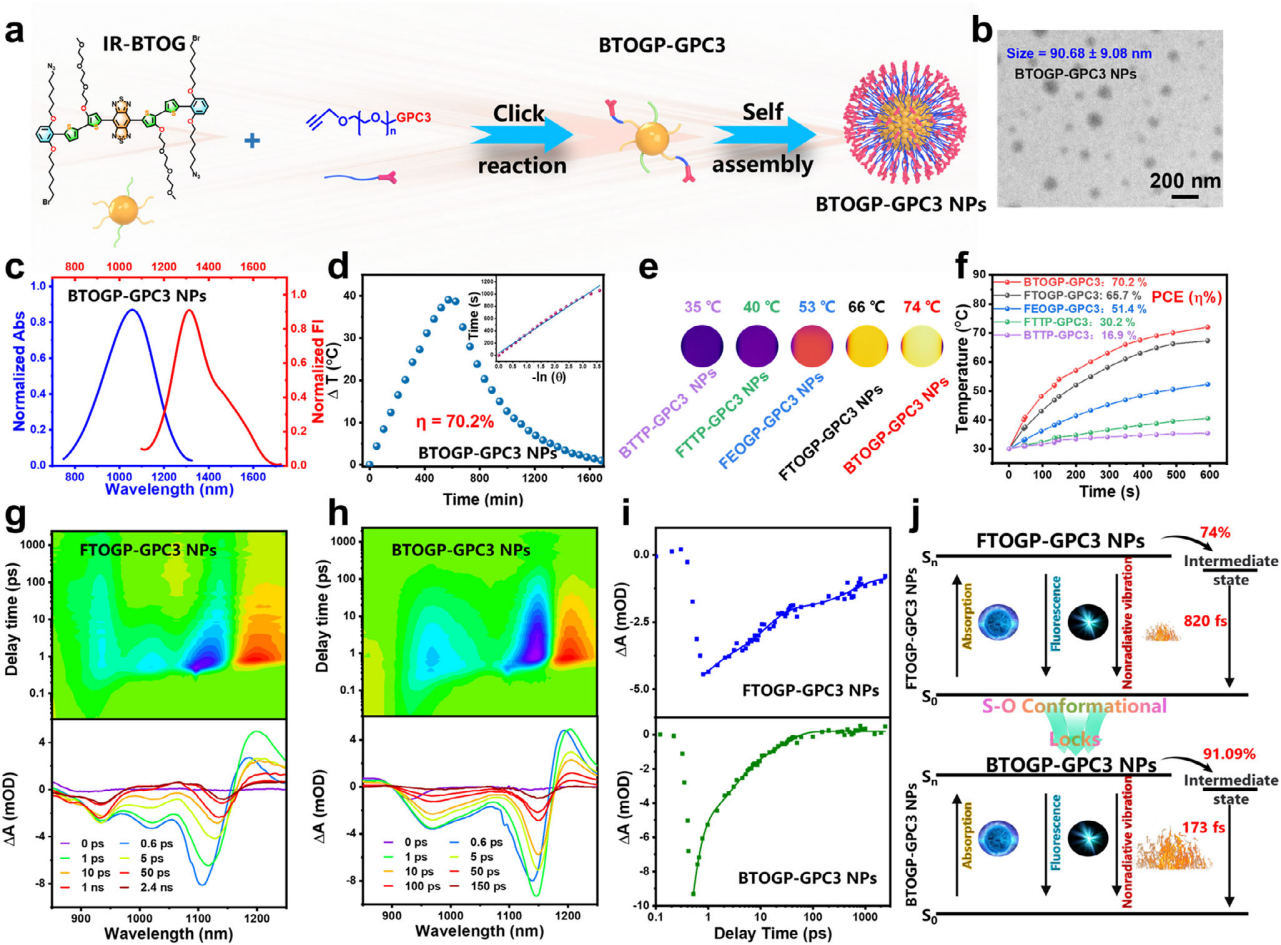
Preparation and analysis of the photothermal capabilities of nanoparticles. a) Preparation of HCC targeting near‐infrared II nanoparticles: IR‐BTOG NPs. b) Transmission electron microscope image of BTOGP‐GPC3 NPs (BTOG‐GPC3 NPs, 80 µm, 200 µL). c) Normalized absorption and emission spectra of IR‐BTOG NPs in aqueous solution (1064 nm, 1.0 W cm^−2^). d) Photothermal images and e) temperature curves of the NPs under 1064 nm laser irradiation (80 µm, 1 mL, 1064 nm, 1.0 W cm^−2^). f) PCE curve of IR‐BTOG nanoparticles (80 µm, 1 mL,1064 nm, 1.0 W cm^−2^). g) 2D Pseudocolour fs‐TA spectra and different pump‐probe delay times fs‐TA spectra of BTOGP‐GPC3 NPs. h) FTOGP‐GPC3 NPs in water following photoexcitation with 1064 nm laser pulses. i) Kinetic decay curves and fitting lines corresponding to ground‐state bleaching (GSB) at the peak absorption. j) Diagram illustrating the mechanism of intermediate‐induced non‐radiative processes. (Error bars: mean ± SD (n = 3)).

Regarding the superior long‐wave penetration and fluorescence characteristics of IR‐BTOG, it was further coupled with GPC3 peptide to target HCC, resulting in IR‐BTOGP‐GPC3 (hereafter referred to as BTOGP‐GPC3 NPs), aiming to achieve exceptional targeted diagnostic and therapeutic efficacy of HCC (**Figure** **4**a). The morphology and size distribution of the samples were analyzed using transmission electron microscopy (TEM) and dynamic light scattering (DLS) (BTOG‐GPC3 NPs, 80 µm, 200 µL). The results show that BTOGP‐GPC3 NPs were spherical with an average diameter of about 90.68 ± 9.08 nm (Figure 4b). The hydrodynamic diameter of BTOGP measured by DLS was 91.94 ± 7.45 nm, consistent with TEM results (Figure S33a, Supporting Information). Figure S33b (Supporting Information) shows that the size and polydispersity index (PDI) of BTOGP‐GPC3 NPs remained stable over 14 days in PBS. In addition, the spectrum of BTOGP‐GPC3 NPs shows a high absorption peak in the range of 1100–1300 nm, which proves that it has an excellent penetration depth of excited light. Fluorescence under 405 nm excitation supports CLSM‐based cellular investigations. (Figure S34, Supporting Information). A broad emission peak is shown in the 1200‐1700 nm range, indicating that BTOGP‐GPC3 NPs have significant potential in bioimaging (Figure 4c).

Subsequently, a comprehensive investigation into the photothermal properties of NIR‐II in five representative nanoparticles, including BTOGP‐GPC3 NPs, FTOGP‐GPC3, FEOGP‐GPC3, FTTP‐GPC3, and BTTP‐GPC3, has been conducted. As shown in Figure 4e, the temperature of all tested nanoparticles (80 µm, 1 mL, in PBS) increased significantly after 10 minutes of irradiation with a 1064 nm laser at the maximum permissible exposure (MPE) power density (1.0 W cm^−^^2^). Among them, the temperature of BTOGP‐GPC3 NPs and FTOGP‐GPC3 NPs reached 74 and 66 °C, respectively. BTOGP‐GPC3 NPs showed the highest temperature rise of all the tested nanoparticles, which may be attributed to their higher molecular flatness. With a 1064 nm laser, the BTTP‐GPC3 NPs, FTTP‐GPC3 NPs, FEOGP‐GPC3 NPs, FTOGP‐GPC3 NPs, and BTOGP‐GPC3 NPs, NPs near‐infrared II photothermal conversion efficiencies (PCE) were 16.9%, 30.2%, 51.4%, 65.7%, and 70.2%, respectively (Figure 4d,e,f). This result further substantiates that BTOGP‐GPC3 NPs with multiple SoCLs attain optimal photothermal conversion efficiency.

Additionally, fs‐TA spectroscopy was used to investigate the underlying reason for the exceptional photothermal performance of BTOGP‐GPC3 NPs. The ground‐state bleaching (GSB) region indicates the deactivation of the excited population primarily by non‐radiative decay.^[^ ^15^, ^40^ ^]^ Investigating the excited state dynamics in the GSB region offers a straightforward strategy for clarifying the NIR‐II photothermal effects caused by materials. The contour plots of Figure 4g,h demonstrate that both samples exhibit positive excited‐state absorption (ESA) bands at ≈1200 nm. Matching with the dynamics of ESA signals, the narrow negative signals at 1141 nm are assigned to GSB signals caused by the depletion of ground states. The almost mirror‐like symmetry between ESA and GSB indicates their similar origin. After reaching the most vigorous intensity, signals of GSB and ESA gradually diminish through some relaxation pathways of the excited states. The fs‐TA plots shown in Figure 4g validate the presence of long‐lived species in FTOGP‐GPC3 NPs, as evidenced by the intense ESA signal observed at ≈1200 nm at delay times of up to 2.4 ns. In comparison, similar ESA signals were observed in the BTOGP‐GPC3 NPs fs‐TA plots with delay times of 150 ps (Figure 4h). The results mentioned above could be preliminarily confirmed: the GSB signal intensities of the BTOGP‐GPC3 NPs diminish more rapidly than those of the FTOGP‐GPC3 NPs, reflecting the accelerated non‐radiative decay of excited states and the more facile non‐radiative decay to promote photothermal conversion efficiency.

Representative kinetic curves acquired in the GSB region demonstrate an expedited non‐radiative process (Figure 4i). Each material was thereafter selected for optimum absorption along the kinetic curve to eliminate the influence of stimulating the emission zone. A sufficient fit for precise dynamic Information necessitates a tri‐exponential model (Table S6, Supporting Information). The non‐radiative component's averaged time constant (***τ***_avg_) was computed by weighting the constituent elements according to their amplitudes. The exceptional non‐radiative decay rate (***τ***_avg_^−1^) of BTOGP‐GPC3 NPs compared to FTOG NPs is the primary factor influencing its greater NIR‐II photoconversion efficiency (PCE). As presented in Table S6 (Supporting Information), the BTOGP‐GPC3 NPs manifest a shorter average half‐lifetime than the FTOGP‐GPC3 NPs (1.069 vs 37.16 ps). It again suggests their expedited non‐radiative decay rates (0.935 vs 0.027 ps^−1^) for superior photothermal efficiency. Moreover, it is worth noting that BTOGP‐GPC3 NPs have a femtosecond component of 173 fs, which is much lower than FTOGP‐GPC3 NPs (820 fs). In addition, up to 91.09% of the excited population is depleted through this ultra‐fast, non‐radiative decay pathway, resulting in the highest photothermal effect.

The results above may be ascribed to the interaction between excited BTOGP‐GPC3 NPs and adjacent ground‐state BTOGP‐GPC3 NPs, leading to the ultrafast depletion of excited atomic population and enhancing NIR‐II photothermal effects. We ascribe that BTOGP‐GPC3 NPs exhibit a more planar configuration than FTOGP‐GPC3 NPs via SoCLs, which is probably significant in that the ultrafast depletion of the excited population produces the superior NIR‐II photothermal. To our knowledge, this PCE is superior among D‐A‐D type nanoparticles exhibiting both NIR‐II fluorescence and NIR‐II photothermal capabilities reported to date (Table S7, Supporting Information), surpassing most other photothermal agents at 1064 nm irradiation. Significantly, BTOGP‐GPC3 NPs fully utilize the remaining 9% of the excited groups to achieve the fluorescence characteristics of NIR‐II (Figure 4j).

### 2.3 Intracellular Phototherapeutic Effect Evaluation

**Figure 5:**
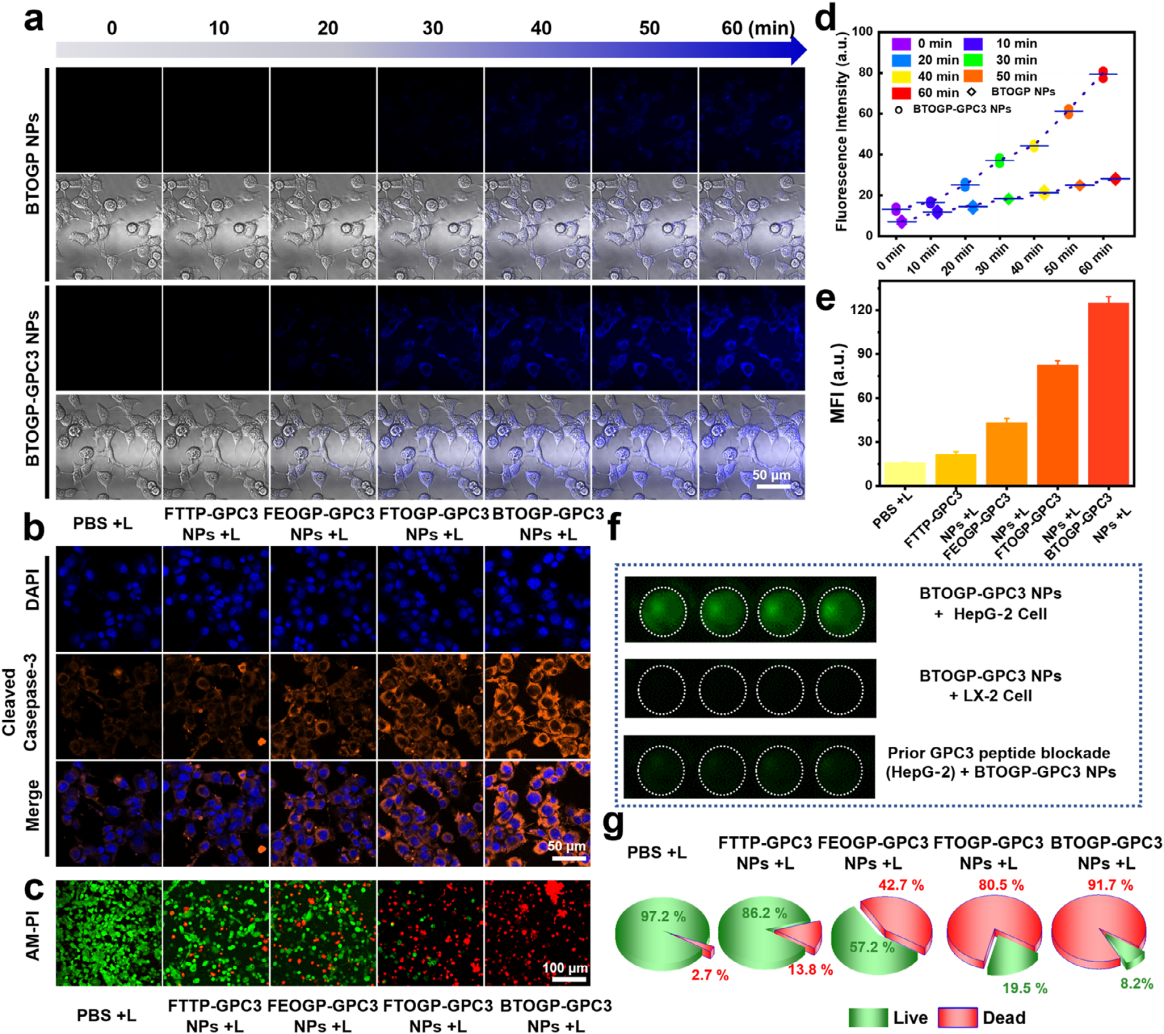
Assessment of cellular targeting, variation in Cleaved caspase levels, and cell viability rates. a) Fluorescence of uptake varied amongBTOGP NPs (80 µm, 100 µL) and BTOGP‐GPC3 NPs (80 µm, 100 µL) throughout 60 minutes (live‐cell images were acquired from a fixed field of view for each experimental group). b) Variations in Cleaved caspase staining among different experimental treatment groups. c) AM–PI index changes among distinct treatments. d) Fluorescence intensity calibration curve corresponding to the inset (a). e) Fluorescence intensity calibration curve corresponding to the inset (b). f) NIR‐II fluorescence image of BTOGP‐GPC3 NPs (80 µm, 100 µL) after co‐incubation with Hep‐G2 cells and LX‐2 cells. Hep‐G2 cells were pre‐blocked by adding 800 µm GPC3 peptide, and after incubation for 1 h, the NIR‐II fluorescence images of non‐specific binding were washed away. g) Pie chart depicting changes in fluorescence signal in inset (c). + L is present as 1064 nm laser irradiation. For the experiments shown in inset (b,c,f,g), all samples were incubated with 100 µL of an 80 µm nanoparticle suspension. Following a wash step to remove non‐specifically bound particles, the samples were subjected to a single 10‐minute laser irradiation (1064 nm, 1.0 W cm^−^^2^) (Error bars: mean ± SD (n = 4).

Further, the HCC targeting capabilities of BTOGP‐GPC3 NPs were systematically evaluated through incubation experiments using HepG‐2 cells as an in vitro HCC model. The results indicated that compared to the BTOGP NPs group, the BTOGP‐GPC3 NPs group exhibited significantly enhanced fluorescence signals after 60 minutes of incubation (**Figure** **5**a). The mean fluorescence intensity of the BTOGP‐GPC3 NPs group escalated almost eightfold from the initial value, while the BTOGP group exhibited an elevation of only 2‐fold (Figure 5d). This remarkable difference indicates that the internalization efficiency of BTOGP‐GPC3 NPs is notably enhanced owing to the active targeting by the GPC3 peptide. Meanwhile, a GPC3 peptide pre‐blocking assay was performed to further verify the targeting effect.As Figure 5f and Figure S35 (Supporting Information) demonstrated, compared with normal LX‐2 cells, HepG‐2 cells exhibited strong fluorescence uptake in the absence of pre‐blocking. A 10‐fold GPC3‐peptide pre‐block sharply diminished cellular fluorescence of BTOGP‐GPC3 NPs, confirming that their tumor targeting depends on peptide‐specific affinity.In conclusion, the Cell targeting experiment results unequivocally demonstrate that GPC3 peptide alteration considerably enhances the molecular targeting of BTOGP‐GPC3 NPs

Cell survival was quantitatively evaluated via the HepG‐2 hepatoma cell and LX‐2 cell counting kit 8 (CCK‐8) (AmBeed) to measure the cytotoxicity of fluorescent compounds modified with various S···O conformation locking and alkoxy engineering strategies. Figure S36a (Supporting Information) shows that within the concentration range of 20–200 µm, FTOGP‐GPC3 nanoparticles, without laser irradiation, have no significant effect on the survivability of HepG2 and LX‐2 cells, with cell survival rates consistently above 90%. Comparable results indicating excellent biocompatibility were seen with BTOGP‐GPC3 nanoparticles, with cell survival rates exceeding 90%. These results indicate that the BTOGP‐GPC3 series of nanoparticles effectively mitigates the inherent biotoxicity of the constituent materials, exhibiting negligible adverse effects in organisms in the absence of laser irradiation. Figure S37 (Supporting Information) demonstrates that the survival rates of BTOGP‐GPC3 NPs +L, FTOGP‐GPC3 NPs +L, FEOGP‐GPC3 NPs +L, FTTP‐GPC3 NPs +L, and PBS NPs +L groups against HepG‐2 cells were 6.8%, 15.8%, 51.8%, 80.2%, and 90.5%, respectively. Immunofluorescent staining for cleaved caspase‐3 (Cas‐3) indicated cellular apoptosis following different treatments. Figure 5b,e show that cleaved caspase staining exhibited no effects in the PBS group after laser irradiation. The levels of cleaved caspase‐3 in the BTOGP‐GPC3 NPs +L, FTOGP‐GPC3 NPs +L, FEOGP‐GPC3 NPs +L, and FTTP‐GPC3 NPs +L groups all exhibited an increase, with the cleaved caspase levels demonstrating the following trends: BTOGP‐GPC3 NPs +L > FTOGP‐GPC3 NPs +L > FEOGP‐GPC3 NPs +L > FTTP‐GPC3 NPs +L. Moreover, live/dead cell staining analyses corroborated the data above. Figure S36b (Supporting Information) demonstrated that in the absence of laser irradiation, all four nanoparticle formulations exhibited excellent biocompatibility, manifesting negligible dark toxicity. Figure 5c,g demonstrate that the BTOGP‐GPC3 NPs + L group exhibited a pronounced capacity for tumor cell cytotoxicity, notably surpassing the efficacy of other control molecules. The above results indicated that BTOGP‐GPC3 NPs exhibited exceptional antitumor activity, primarily because of the substantial variation in photothermal characteristics of BTOGP‐GPC3 NPs after optimization by S···O conformation locks and alkoxy engineering strategies.

In summary, BTOGP‐GPC3 NPs, optimized by SoCLs synergistic alkoxy engineering strategies, achieved selective targeting of tumor cells via GPC3 peptide, demonstrating remarkable anti‐HCC effectiveness upon illumination triggering, as demonstrated in HepG‐2 cells.

### 2.4 In Vivo NIR‐II Fluorescence Imaging

**Figure 6:**
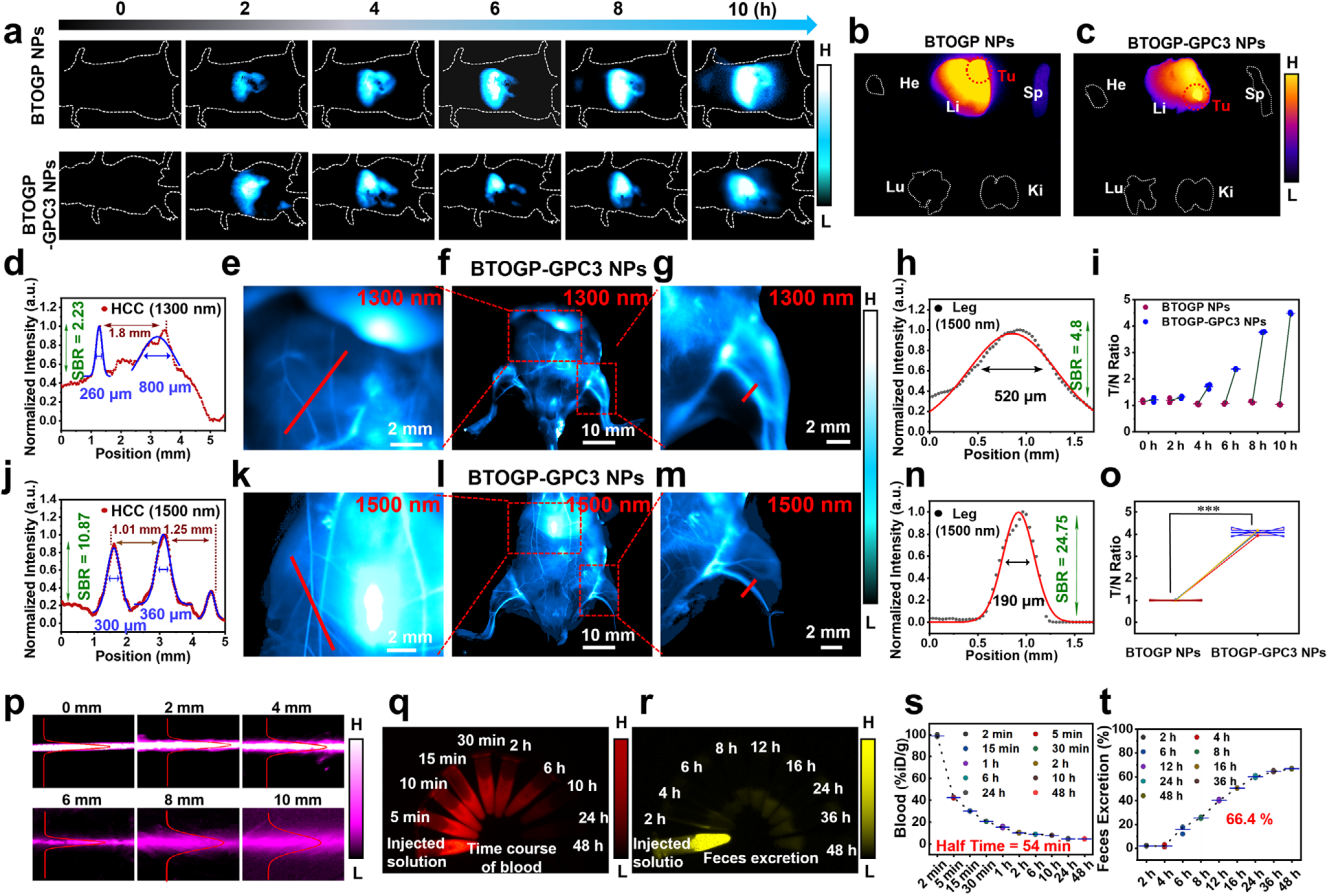
In vivo NIR‐II imaging of hepatic carcinoma and nanoparticle metabolism. a) Targeted NIR‐II fluorescence imaging of orthotopic HCC using BTOGP and BTOGP‐GPC3 NPs (IV dose: 200 µM, 200 µL). Imaging parameters: power density 45 mW·cm^−^²; laser height 40 cm; illuminated area 144 cm²; exposure 1000 ms. b,c) Tumor and major‐organ NIR‐II fluorescence for BTOGP/BTOGP‐GPC3 NP groups at 10 h. d) NIR‐II vessel‐intensity distribution (scatter) with Gaussian fit (blue) near HCC after BTOGP‐GPC3 NPs. BALB/c nude, orthotopic model; two tail‐vein injections (200 µL of 200 µm) 8 h later. Imaging: 1064 nm, 45 mW·cm^−^², 30 cm height, 81 cm² field, 1300‐nm LP, 50 ms; within 30 min. e) NIR‐II fluorescent images of BTOGP‐GPC3 NPs (Intravenous injection dose of nanoparticles: 200 µm, 200 µL, irradiation of the chest, abdomen, and legs. The parameters of imaging: 1064 nm excitation, power density, 45 mW cm^−2^, laser height, 30 cm; laser area, 81 cm^2^, 1300 nm long‐pass filter, exposure time:50 ms, within 30 minutes), illustrating the imaging effects on the hepatocellular carcinoma, whole body (f), and leg (g), respectively. h)NIR‐II leg‐vessel intensity distribution (scatter) and corresponding Gaussian fit (red) with a 1300‐nm long‐pass filter after BTOGP‐GPC3 NPs. i) Comparison of the tumor to normal tissue (T/N) ratio of inset (a). j) NIR‐II vessel intensity around HCC: scatter data with a blue Gaussian fit after BTOGP‐GPC3 NPs. (Intravenous injection dose of nanoparticles: 200 µm, 200 µL, irradiation of the chest, abdomen, and legs. The parameters of imaging: 1064 nm excitation, power density, 45 mW cm^−2^, laser height, 30 cm; laser area, 81 cm^2^, 1500 nm long‐pass filter, exposure time:50 ms, within 30 minutes). k) NIR‐II fluorescent images of BTOGP‐GPC3 NPs (Intravenous injection dose of nanoparticles: 200 µm, 200 µL, irradiation of the chest, abdomen and legs. 45, illustrating the imaging effects on the HCC, whole body (l), and leg (m), respectively. n) NIR‐II fluorescence intensity distribution curve (scatter) and Gaussian fitting curve (blue) of leg blood vessels with a 1500 nm long‐pass filter using BTOGP‐GPC3 NPs. o) Comparison of the T/N ratio in insets (b,c). p) NIR‐II fluorescence imaging of BTOGP‐GPC3 NPs (Concentration of nanoparticles: 200 µm. within capillary tubes wrapped in different thicknesses of chicken. q) Time course of blood concentration of BTOGP‐GPC3 NPs (Intravenous injection dose of nanoparticles: 200 µm, 200 µL, irradiation of the chest, abdomen, and legs. The parameters of imaging: 1064 nm excitation, power density, 45 mW cm^−2^, laser height, 30 cm; laser area, 81 cm^2^, 1500 nm long‐pass filter, exposure time:50 ms) ‐treated mic in 48 hours and s) corresponding semi‐quantitative analysis of blood samples. r) Cumulative feces excretion fluorescence imaging and t) corresponding semi‐quantitative analysis. The significance of the difference between more than two groups was determined via an ANOVA‐LSD *post hoc* test. * *P *< 0.05, ** *P *< 0.01, *** *P *< 0.001. Error bars: mean ± SD (n = 4).

Inspired by the excellent phototheranostic performance of BTOGP‐GPC3 NPs in vitro, the potential of these NPs for phototheranostics in vivo was further investigated. Firstly, the NIR‐II fluorescence imaging performance of BTOGP‐GPC3 NPs (excited at 1064 nm) was assessed using an in situ tumor‐bearing mouse model of HepG‐2 cells. As shown in **Figure** **6**a, compared with group BTOGP NPs (200 µm, 200 µL), the fluorescence signal of group BTOGP‐GPC3 NPs (200 µm, 200 µL) at the tumor location markedly aggregated throughout time and stabilized after 8 hours. To further validate the in vivo distribution of BTOGP‐GPC3 NPs, fluorescent pictures of isolated organs were obtained by dissecting euthanized mice. As Figure 6b,c illustrate, in contrast to the BTOGP NPs group, the BTOGP‐GPC3 NPs group exhibited a strong aggregation ability at the tumor location, underscoring the superior targeting efficacy of GPC3‐modified fluorescent molecules in vivo.

Furthermore, with the spleen and liver serving as the primary distribution organs of the mononuclear phagocytic system (MPS), BTOGP and BTOGP‐GPC3 NPs exhibit specific accumulation in these organs. Notably, BTOGP‐GPC3 NPs outlined HCC more distinctly than BTOGP NPs, achieving a high image tumour‐to‐normal tissue (T/N) signal ratio from 4.26 (Whole mouse body: Figure 6i) to 4.10 (mouse organ: Figure 6o). This result demonstrates that BTOGP‐GPC3 NPs significantly enhance tumor imaging clarity, establishing a robust basis for further tumor detection and therapy use. In addition, BTOGP‐GPC3 NPs (200 µm, 200 µL) were delivered intravenously into tumor‐bearing BALB/c nude mice (approximately 9–10 hours after the pre‐intravenous injection of BTOGP‐GPC3 nanoparticles). Under the excitation of a 1064 nm laser, BTOGP‐GPC3 NPs effectively achieved fast imaging of the principal blood arteries (1064 nm excitation, within 30 minutes). The imaging results of the upper abdomen and legs were further analyzed using 1300 and 1500 nm long‐pass filters (Figure 6f,l). In the 1300 nm filter imaging mode, the two neighboring major blood arteries surrounding the HCC exhibited blurring at distances of 1.8 mm, respectively, with a signal‐to‐noise ratio (SBR) of 2.23, while the half‐height full width (FWHM) measured 260 and 800 µm (Figure 6d,e). Conversely, with the 1500 nm filter, the delineation of blood vessel contours surrounding the HCC was more distinct, with the intervascular distances measuring 1.01 and 1.25 mm, respectively. The signal‐to‐noise ratio improved to 10.87, while the full width at half maximum (FWHM) recorded values of 300 and 360 µm (Figure 6j,k). Furthermore, NIR‐II imaging in leg imaging may identify four contiguous blood vessels. Compared to 1300 nm filter imaging (SBR: 4.8, FWHM: 520 µm) (Figure 6g,h), 1500 nm filter imaging exhibited superior resolution and picture quality (SBR: 24.75, FWHM: 190 µm) (Figure 6m,n).

Based on the stronger performance of the optimized BTOGP‐GPC3 NPs in the long‐wave spectrum, the imaging penetration of BTOGP‐GPC3 NPs inside the NIR‐II window (1500 nm filter) was evaluated by filling the capillary and depositing a 1% lipid layer of different thicknesses. The analysis of the signal fitting curve for the experimental group with differing lipid layer thicknesses demonstrates that BTOGP‐GPC3 NPs have exceptional imaging penetration capabilities, achieving an optical penetration depth of 8–10 mm (Figure 6p). The results indicate that BTOGP‐GPC3 NPs possess considerable promise for applications in deep tissue bioimaging.

To investigate the in vivo pharmacokinetics of BTOGP‐GPC3 NPs, the extraction of blood and feces samples was monitored at different times. As shown in Figure 6q–t, the blood circulation and feces excretion of BTOGP‐GPC3 NPs were noted during the 48 hours that followed intravenous injection. The half‐time of circulation for BTOGP‐GPC3 NPs was ≈54 min. In addition, the feces excretion data showed that ≈66.4% of BTOGP‐GPC3 NPs were excreted via feces within 48 hours post intravenous injection, demonstrating slow processing of hepatic excretion.

### 2.5 In Vivo Antitumor Efficacy

**Figure 7:**
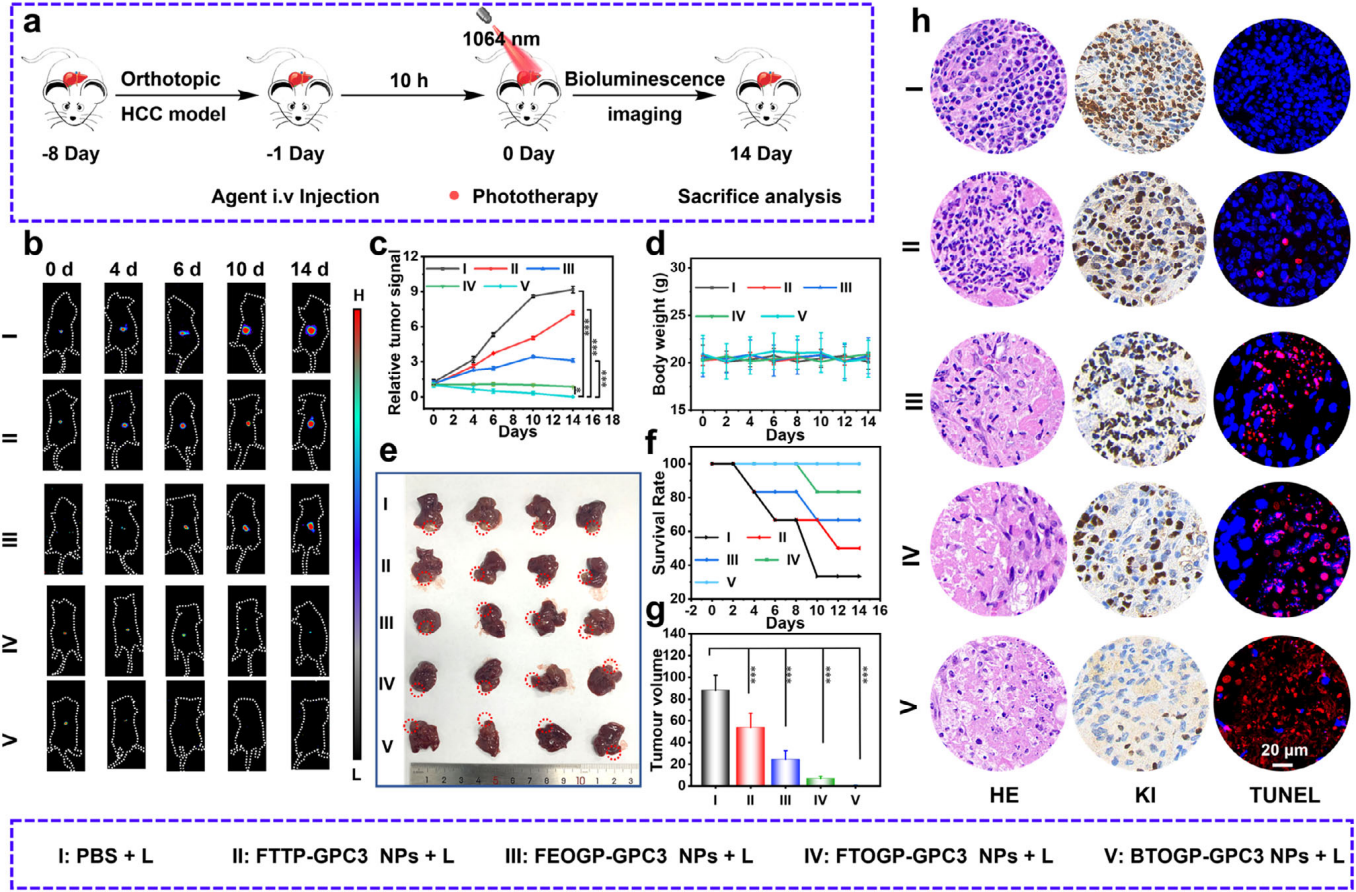
Therapy validation in an in situ HCC model. a)Schematic of orthotopic HCC establishment and the treatment workflow. b) SCID mouse bioluminescence for each treatment group at days 0, 2, 4, 6, 10, and 14 (Take the representative luminescence image of one mouse from each experimental group). c) Hepatocellular tumor growth curves, d) mouse weight changes, e) excised livers containing the tumor, and f) Mouse survival by treatment group. g) Excised liver tumor sizes. h) Day‐14 liver histology: H&E, Ki‐67, and TUNEL.+ L is present as 1064 nm laser irradiation. The treatment methods of different groups in Figure 7 are as follows: I: PBS + L, II: FTTP‐GPC3 NPs + L, III: FEOGP‐GPC3 NPs + L, IV: FTOGP‐GPC3 NPs + L, V: BTOGP‐GPC3 NPs + L. All Fig. 7 groups received tail‐vein injections (NP dose: 200 µm, 200 µL). Irradiated once with 1064 nm, 1.0 W cm^−2^ laser for 10 min, irradiation of the tumor, and acted at the time of the highest aggregation concentration of nanoparticles for 10 h. The significance of the difference between more than two groups was determined via an ANOVA‐LSD *post hoc* test. * *P *< 0.05, ** *P *< 0.01, *** *P *< 0.001. Error bars: mean ± SD (n = 4).

Based on the above experimental results, in vivo experiments further demonstrated the efficacy of light diagnosis and therapy under the optimized strategy. To get a non‐invasive visualization of tumor size, HCC cells were first pre‐transfected with luciferase. During a 14‐day interval, bioluminescent signals at luciferase‐labeled tumor sites were observed via an in vivo imaging system (**Figure** **7**a), and tumor volume was evaluated based on luminous intensity. Figure 7b,c illustrate that under laser irradiation, the tumor growth rate in the FTTP‐GPC3 NPs and PBS groups was significantly enhanced, but tumor suppression was observed in the FTOGP‐GPC3 NPs, FEOGP‐GPC3 NPs, and BTOGP‐GPC3 NPs groups. The inhibition rate of hepatoma carcinoma was FTTP‐GPC3 (39.7%) < FEOGP‐GPC3 (72.2%) < FTOGP‐GPC3 (92.0%) < BTOGP‐GPC3 NPs (99.8%) group (Figure 7g). The relative body weights of all groups were unaffected during the treatments, suggesting the non‐toxicity of these molecular phototheranostic agents (Figure 7d). Under experimental protocols, murine hepatic tissues were surgically resected and analyzed across different conditions. Figure 7e illustrates that the liver of mice in the BTOGP‐GPC3 NPs group had a vibrant red hue with a minimal tumor region, corroborating the findings from bioluminescence imaging. As depicted in Figure 7f, the BTOGP‐GPC3 NP treatment group exhibited sustained tumor suppression following initial tumor elimination, with no evidence of tumor recurrence observed over the 14‐day observation period. Consequently, the survival rate in this group remained at 100%. In contrast, the control groups displayed varying degrees of tumor progression and mortality, indicating a significantly reduced therapeutic efficacy compared to the BTOGP‐GPC3 NPs treatment.

H&E staining was used to assess tumor apoptosis (Figure 7h). Apoptotic cells were markedly enhanced in tumor sections, and the expression of TUNEL was heightened. The substantial downregulation of the proliferative marker Ki‐67 indicated a considerable inhibition of tumor cell growth, coupled with increased apoptosis. Moreover, H&E staining pictures and blood biochemical analysis (Figures S38 and S39, Supporting Information) demonstrated that BTOGP‐GPC3 NPs exhibited favorable biocompatibility.

## 3 Conclusion

In summary, this study incorporated an S···O conformational lock and tailored alkoxy chains strategy, resulting in an IR‐BTOG molecule with comprehensive phototheranostic characteristics. Furthermore, the BTOGP‐GPC3 molecule was enhanced by conjugation with the GPC3 target, prominently expressed in HCC, to facilitate precise identification of HCC and effective detection and therapy. The findings from in vivo and in vitro investigations indicate that the S···O conformational lock and alkoxy chain modification technique may successfully address the constraints on the energy conversion efficiency of phototheranostic molecules. IR‐BTOG molecules exhibit increased photothermal conversion efficiency. The fluorescence quantum yield is markedly elevated in the NIR‐IIb range (1500–1700 nm). Research indicates that the BTOGP‐GPC3 molecule has exceptional overall characteristics in diagnosing and treating advanced tumors, serving as a potent molecular instrument for accurately diagnosing and managing HCC. This study's revolutionary design overcomes performance limitations of optical diagnostic and therapeutic molecules in excitation band, emission band, and energy usage efficiency, offering a novel method and optimism for detecting and treating deeply resistant cancers.

## 4 Experimental Section

The experimental section is available in the Supporting Information. The University of South China Animal Experiment Ethics Review and the Health Guide for the Care and Use of Laboratory Animals of the National Institutes approved all animal experiments. The assigned accreditation number of the laboratory is 2023027.

### Statistical Analysis

Statistical analyses were performed using SPSS 26.0. software. All results are expressed as the mean ± standard deviation (SD) from a minimum of three independent experiments. The significance of the difference between more than two groups was determined via an ANOVA‐LSD *post hoc* test. * *P *< 0.05, ** *P *< 0.01. ***, *P *< 0.001. Error bars: mean ± SD (n = 4).

## Conflict of Interest

The authors declare no conflict of interest.

## Author Contributions

G.‐L.W. and F.W. contributed equally to this work. All authors have approved the final version of the manuscript.

## Supporting information



Supporting Information

## Data Availability

The data that support the findings of this study are available from the corresponding author upon reasonable request.
