# Supplementary material for: S···O Conformation Locks Synergistic Alkoxy Chain Engineering of NIR‐II Phototheranostic Molecules for Precision Hepatocellular Carcinoma Theranostics
Source: Adv Sci (Weinh). 2025 Nov 28;13(1):e06664. doi: 10.1002/advs.202506664 (PMC12767041; doi:10.1002/advs.202506664)
Supplement: Supplementary file 1 — Supporting Information [file ADVS-13-e06664-s001.pdf]

Supporting Information

S-O Conformation Locks Synergistic Alkoxy Chain Engineering of NIR-II Phototheranostic Molecules for Precision Hepatocellular Carcinoma Theranostics

Gui-long Wu[1,2‡], Fan Wu[1,2‡], Senyou Tan[1,2], Hao Xiao[1,2], Qiang Kang[1,2], Sanlin Deng[1,2], Fen liu[1,2,4], Jinkang Zheng[1,2], Chaoqiang Li[1,2], Guodong Chen[1,2,3], Qinglai Yang[1,2,3]**

[1] Department of Hepatopancreatobiliary Surgery, The First Affiliated Hospital, Hengyang Medical School, University of South China, Hengyang, Hunan, 421001, China.

[2] Center for Molecular Imaging Probe, Cancer Research Institute & Hunan Engineering Research Center for Early Diagnosis and Treatment of Liver Cancer & MOE Key Lab of Rare Pediatric Disease & NHC Key Laboratory of Birth Defect Research and Prevention, Hengyang Medical School, University of South China, Hengyang, Hunan 421001, China.

[3] Department of General Surgery, Turpan City People's Hospital, Tulufan 838000, China.

[4] Department of Radiology, The Second Affiliated Hospital, Hengyang Medical School, University of South China, Hengyang, Hunan 421001, China.

Correspondence should be addressed to: chenguodong@usc.edu.cn, qingyu513@usc.edu.cn

‡ These authors contributed equally to this work.

1

Table of Contents

1. Experimental section 6

1.1 Materials and reagents 6

1.2 General measurements 6

1.3 Density functional theory calculations 7

1.4 Molecular dynamics (MD) simulations. 7

1.5 *In vitro* photothermal and photothermal conversion efficiency assessment 9

1.6 Measurement of quantum yield 10

1.7 Tumor cell targeting assays 11

1.8 Live/Dead Cells Staining 11

1.9 CCK8 Assay 11

1.10 Immunofluorescence staining 12

1.11 Establishment of HepG-2 Tumor Models 12

1.12 NIR-II Fluorescence imaging 13

1.13 Pharmacokinetics, biodistribution, and excretion of BTOG-GPC3 NPs 14

1.14 Phototherapy of *in situ* tumor mice 15

1.15 Pathologic analysis 16

1.16 Ethical Approval 16

1.17 Statistical Analysis 16

1.18 Synthetic procedures and characterization data for the molecular fluorophores. 17

2. Supplementary Figures 31

Figure S1. The NIR-II D-A-D type organic molecule was previously designed and synthesized by our research group[8]. 31

Figure S2. ^{1}H NMR of compound 4 32

Figure S3. ^{13}C NMR of compound 4 32

Figure S4. HRMS of compound 4 33

Figure S5. ^{1}H NMR of compound 7 33

Figure S6. ^{13}C NMR of compound 7.......34
Figure S7. HRMS of compound 7.......34
Figure S8. ^{1}H NMR of compound **IR-BTOG**. 35
Figure S9. ^{13}C NMR of compound **IR-BTOG**. 35
Figure S10. HRMS of compound **IR-BTOG**. 36
Figure S11. ^{1}H NMR of compound **BTOGP-GPC3**. 36
Figure S12. ^{13}C NMR of compound **BTOGP-GPC3**.......37
Figure S13. ^{1}H NMR of compound 12.......37
Figure S14. ^{13}C NMR of compound 12.......38
Figure S15 HRMS of compound 12.......38
Figure S16. ^{1}H NMR of compound **IR-FEOG**.......39
Figure S17. ^{13}C NMR of compound **IR-FEOG**. 39
Figure S18. HRMS of compound **IR-FEOG**.......40
Figure S19. ^{1}H NMR of compound **FEOGP-GPC3** 40
Figure S20. ^{13}C NMR of compound **FEOGP-GPC3**.......41
Figure S21. ^{1}H NMR of compound **15**.......41
Figure S22. ^{13}C NMR of compound **15.**.......42
Figure S23. HRMS of compound **15.**.......42
Figure S24. ^{1}H NMR of compound **IR-FTOG.**.......43
Figure S25. ^{13}C NMR of compound **IR-FTOG.**.......43
Figure S26. HRMS of compound **IR-FTOG.**.......44
Figure S27. ^{1}HNMR of compound **FTOG-GPC3.**.......44
Figure S28. ^{13}C NMR of compound **FTOG-GPC3.**.......45
Figure S29. Molecular dynamics simulations of IR-FTOG and IR-BTOG. 48
Figure S30. Nanoparticle size (a) and (b) 14-day stability of BTOGP-GPC3 nanoparticles.48
Figure S31. (a) Absorption spectra of BTOG-GPC3 NPs (300–1500 nm). (b) fluorescence emission spectra of BTOG-GPC3 NPs (λex = 405 nm).......49
Figure S32. Photothermal imaging of mice with orthotopic hepatocellular carcinoma with

different treatments. .... 错误!未定义书签。

Figure S34. (a) The CCK8 cell viability assays of FTOGP-GPC3 NPs -L and BTOGP-GPC3 NPs -L. (b) The Calcein-AM/Propidium Iodide (AM/PI) live/dead cell staining assays of FTTP-GPC3 NPs -L, BTOGP-GPC3 NPs -L, FTOGP-GPC3 NPs -L and BTOGP-GPC3 NPs -L..50

Figure S35. Cell viability of HepG-2 cells with different treatments. 50

Figure S36. Whole blood and blood biochemistry of healthy BALB/c mice treated with PBS + L, BTOG-GPC3 NPs + L for 14 days. Error bars, mean ± SD (n = 3). 51

Figure S37. H&E staining images of significant organs from healthy BALB/c mice treated with PBS + L, BTOG-GPC3NPs + L. 52

3. Supplementary tables 53

Table S1. Calculated first vertical S_0-S_1 excitation energies (E_{01}), first vertical S_1-S_0 emission energies (E_{10}), electronic configurations, and reorganization energies determined at the B3LYP */6-31G g (d,p) level...53

Table S2. The optimized dihedral angles (θ), orbital plane angles (α), bond lengths (*d*, Δ*d*=*d*v–*d* (*d*v: The values of the sum of van der Waals radii of two atoms)), and descriptors $S = -\cos\alpha \bullet \cos 2\theta 1 - e\Delta d 2$ for IR-FEOG, IR-FTOG, IR-BTG and IR-BTOG. 54

Table S3. Cartesian coordinates for DFT optimized structure of IR-BTOG. 56

Table S4. LUMO composition analysis results of the molecular fluorophores IR-FEOG, IR-FTOG, IR-BTG, and IR-BTOG based on the Hirshfeld method by Multiwfn. 61

Table S5. Optical data of NIR-II fluorophores 62

Table S6. Fitting parameters of ground states and excited states of materials. 63

Table S7. Summary of photothermal agents (PTAs) for phototheranostics application. (η = photothermal conversion efficiency，1064 nm)..64

4. Reference 67

1. Experimental section

1.1 Materials and reagents

The Cell Counting Kit (CCK8), AM/PI staining kit, M Matrigel, and D-Luciferin potassium salt were purchased from Sigma-Aldrich Co., HepG-2-luc cell (human hepatocellular carcinoma Cell Line-Luciferase Labeled) and LX-2 cell (normal liver cells) were obtained from ATCC (American Type Culture Collection). Calcein-AM/PI double stain kit was purchased from Abbkine (KTA1001, Abbkine, Wuhan, China). GPC3 peptides and Alkynyl-PEG_{1000}-GPC3 were purchased from Chinapeptides Co., Ltd. Chemicals for organic synthesis were ordered from Shanghai Titan Scientific Co. Tetrahydrofuran (THF) and Dimethyl formamide (DMF) used for reactions were purified by a solvent purification system (Innovative Technology, Inc.) before use. All air and moisture-sensitive reactions were carried out in flame-dried glassware under a nitrogen atmosphere.

1.2 General measurements

Nuclear magnetic resonance (NMR) spectra were recorded on an AVANCE NEO 500 spectrometer (Bruker, Germany) using $CDCl_3$ as the internal reference. High-resolution mass spectra (HRMS) were obtained on a Thermo Scientific Q Exactive Combined quadrupole Orbitrap mass spectrometer (Thermo Fisher Scientific Co, USA) in a Positive ion mode. Confocal laser scanning microscopy (CLSM) images were collected on a Zeiss LSM880 (Zeiss, Germany). Transmission electron microscopy (TEM) images were performed on an HT7800 transmission electron microscope (Hitachi

Electronics, accelerating voltage 80 kV). Typically, the nanomorphology of BTOG-GPC3 was photographed by TEM. The size distributions of the NPs were obtained from Nano-ZS90 (Malvern, China). Fluorescence spectra were measured on a Thermo Scientific Lumina fluorescence spectrometer (Thermo Fisher Scientific Co., USA). NIR-II fluorescence imaging was performed on SD640 (Xi'an Tianying Optoelectronics Technology Co., China). Ultraviolet-visible-near infrared (UV-Vis-NIR) absorption spectra were measured by a UA-3200S spectrometer (MAPADA, China). Temperature evolution curves were tested by an infrared thermal imaging camera (Fotric 225s, China) upon irradiation with a 1064 nm near-infrared (NIR) laser (1.0 W/cm^2, MDL-XF-1064 nm/10W, Changchun New Industries Optoelectronics Technology Co, Ltd.). The pathological sections were observed *via* Pannoramic DESK (3D HISTECH, HUN).

1.3 Density functional theory calculations

All the calculations were performed using the Gaussian 09 software. The ground-state (S_0) geometries of the simplified structures IR-BTG, IR-FEOG, IR-FTOG, and IR-BTOG were firstly optimized using the B3LYP/6-311+G (d, p) level and re-optimized [1]. Each molecule's corresponding range-separation parameter (ω, in Bohr−1) was optimally tuned according to the GAP-tuning method and listed in Table S1.

1.4 Molecular dynamics (MD) simulations.

Classical molecular dynamics (MD) simulations were carried out to investigate IR-BTG, IR-FEOG, IR-FTOG, and IR-BTOG molecules[2]. The initial configuration of the simulated box had dimensions of 18.96 × 18.96 × 14, with IR-BTG, IR-FEOG, IR-

FTOG, and IR-BTOG molecules positioned at the center of the box. The number of molecules was 100. The initial configurations were constructed using the PACKMOL43 software. The Charmm force field was employed to describe the system. The molecular force field consists of nonbonded and bonded interactions. The nonbonded interactions contain van der Waals (vdW) and electrostatic interaction, defined by Equations S1 and S2, respectively.

$$E_{LJ}(r_{ij}) = 4\varepsilon_{ij}\left(\left(\frac{\varepsilon_{ij}}{r_{ij}}\right)^{12} - \left(\frac{\varepsilon_{ij}}{r_{ij}}\right)^{6}\right) \quad \text{(S1)}$$

$$E_1(r_{ij}) = \frac{q_i q_j}{4\pi\varepsilon_o\varepsilon_r k_\gamma r_{ij}} \quad \text{(S2)}$$

The Lorentz−Berthelot mix rules were adopted for different kinds of atoms for vdW interactions, described in Equation S3. The cutoff distance of vdW and electronic interactions was set to 1.2 nm, and the particle mesh Ewald (PME) method was employed to calculate long−range electrostatic interactions.

$$\sigma_{ij} = \frac{1}{2}(\sigma_{ii} + \sigma_{jj});\ \varepsilon_{ij} = (\varepsilon_{ii} \times \varepsilon_{jj})^{\frac{1}{2}} \quad \text{(S3)}$$

For the simulation, energy minimization was first employed to relax the simulation box. Then, a canonical (NVT) ensemble with a 1.0 fs time step was employed to optimize the simulation box, where the temperature was set to 298 K. The temperature was maintained at 300 K *via* the Nose−Hoover thermostat. The optimization time was 200.0 ns, long enough to obtain a stable system. The trajectory coordinates of molecules were collected with a storage frequency of 20000 steps. In all the MD simulations, the motion of atoms was described by classical Newton's equations, which were solved

using the velocity−Verlet algorithm. All simulations were performed using the Gromacs 2019.6 package.

1.5 *In vitro* photothermal and photothermal conversion efficiency assessment

The photothermal characteristics of the nanoparticles were assessed in vitro using a laser with a wavelength of 1064 nm (1.0 W/cm²). BTG-GPC3 NPs, FEOG-GPC3 NPs, FTOG-GPC3 NPs, and BTOG-GPC3 NPs (50 μM PBS solution) were individually exposed to laser irradiation for 10 minutes, with thermal imaging data collected at 2-minute intervals. The sample solution volume was 1 ml, and the laser irradiation duration was 10 minutes, during which the temperature variation was observed via a thermal imager. Upon completion of the laser on/off cycle test, the photothermal conversion efficiency (PCE, η) is calculated using the formula S4-S8.

$$\eta = \frac{hS(T_{max} - T_{surr}) - Q_0}{I(1 - 10^{-A_\lambda})} \quad \text{(S4)}$$

The following equation can calculate hS:

$$hS = \frac{\sum m_i \, C_{P.i}}{\tau_s} \quad \text{(S5)}$$

$$\tau_s = \frac{\mathrm{t}}{-\ln \theta} \quad \text{(S6)}$$

$$\theta = \frac{\mathrm{T} - T_{surr}}{T_{max} - T_{surr}} \quad \text{(S7)}$$

$$Q_0 = \mathrm{hs}(T_{max} - T_{surr}) \quad \text{(S8)}$$

h represents the heat transfer coefficient,

s represents the sample container surface area,

T_{max} represents the steady-state maximum temperature,

T_{surr} represents the ambient room temperature,

T represents the instantaneous temperature during cooling,

t represents the time it takes for T to cool to room temperature,

C is approximately to the specific heat capacity of water,

m represented the mass of the solution (g),

Q_0 represents the energy input by the same solvent without NPs in the same quartz cuvette after laser irradiation.

1.6 Measurement of quantum yield.

The fluorescence quantum yield of the fluorophore was determined using the technique outlined in the reference [3]. The fluorescence spectra were obtained in the 900-1800 nm region using a 1064 nm laser (MDL-XF-1064 nm, 160 mW) for excitation, with a fiber optic spectrometer (NIR-17S) and a 1100 nm long pass filter (Thorlabs) applied as the emission filter. The fluorescence quantum yield was ascertained by referencing the fluorophore IR-26[4], which has a quantum yield of 0.05% in DCM solution. In the experiment, the optical density (OD) of the reference fluorophore IR-26 was established at 0.08, and the fluorescence emission intensity of the test sample was assessed under identical excitation conditions of 1064 nm. The quantum yield of the test sample is determined using the optical density (OD) and spectrum-integrated fluorescence intensity (F) at a wavelength of 1064 nm, according to the following formula S9-S10:

$$\Phi_x(\lambda) = \Phi_{st}(\lambda) \times \frac{F_x}{F_{st}} \times \frac{A_{st}(\lambda)}{A_x(\lambda)} \qquad \text{(S9)}$$

$$= \Phi_{st}(\lambda) \times \frac{F_x}{F_{st}} \times \frac{1 - 10^{-OD_{st}(\lambda)}}{1 - 10^{-OD_x(\lambda)}} \qquad \text{(S10)}$$

1.7 Tumor cell targeting assays

BTOGP NPs and BTOG-GPC3 NPs solutions (80 μM, 200μL) were co-incubated with HepG-2 cells. The cells were thoroughly rinsed with PBS and investigated with a confocal laser scanning microscope for fluorescent signals. The cell targeting was further confirmed using the NIR-II fluorescence imaging system. BTOG-GPC3 NPs solution (80 μM, 200μL) was co-incubated with HepG-2 cells, LX-2 cells, and HepG-2 cells pre-treated with 800 μM GPC3 (to block specific binding), followed by semi-quantitative fluorescent signal analysis.

1.8 Live/Dead Cells Staining

HepG-2 cells were cultivated in Dulbecco's Modified Eagle Medium (DMEM) supplemented with 10% fetal bovine serum (FBS) and 1% penicillin-streptomycin (PS). The cells were injected at 37°C and 5% CO_2 with a density of 3 × 10^5 cells per dish. Following overnight incubation, the medium was replaced with a solution containing several experimental treatments at a concentration of 80 μM. Following a 4-hour incubation, the cells were rinsed with PBS and stained with CALcIN-AM/PI. Ultimately, confocal laser scanning microscopy (CLSM) was used for analysis.

1.9 CCK8 Assay

The cytotoxicity was evaluated by CCK8 assay. HepG-2 cells or LX-2 cells were seeded into a 96-well plate at the density of 1 × 10^4 cells/well, and cultured in RPMI1640 supplemented 10% fetal bovine serum (FBS, Gibco), 5% CO_2 at 37°C,

penicillin (100 U/mL) and streptomycin (100 μg/mL) for 48 hours. The incubation media were replaced with fresh ones for different treatments, respectively. After 4 hours of co-incubation, the cells were added to CCK8 solution for another 1 hour, and the absorbance at 450 nm was measured in a Biotek/800TS (Berten, USA).

1.10 Immunofluorescence staining

HepG-2 cells were cultured in confocal dishes and treated with the PBS group, FTTG-GPC3 NPs group, FEOG-GPC3 NPs group, FTOG-GPC3 NPs group, and BTOG-GPC3 NPs group. The following was irradiated with a 1064 nm laser (power density 1.0 W/cm^2, duration 10 minutes). The cells were fixed with a 4% paraformaldehyde solution for 15 min. Excess protein binding sites were subsequently eliminated by incubating the cells in a blocking buffer for 60 min. The cells were subsequently exposed to primary antibodies targeting cleaved caspase-3 for a duration of 2 h. Subsequently, the cells were treated with the appropriate secondary antibodies for 1 h at ambient temperature. Following this, the cells were stained with DAPI for 5 min, washed with PBS, and fluorescence images were acquired. The corresponding images were imported into ImageJ software for fluorescence intensity quantification analysis.

1.11 Establishment of HepG-2 Tumor Models

All animal experiments in this study complied with the "Guidelines for the Care and Use of Laboratory Animals at the University of South China" and were approved by the Animal Ethics Committee of the University of South China. HepG-2 cells (2×10^7 cells/mL) were injected subcutaneously under the peritoneum of the lower segment of

the left outer lobe of the liver in SCID mice or Balb/c nude mice (5 weeks old, male) to establish an in situ transplantation tumor model. The tumor was determined by intraperitoneal injection of the substrate D-Luciferin potassium salt. A bioluminescence assay was carried out under the IVIS to determine whether or not the tumor had become a tumor.

1.12 NIR-II Fluorescence imaging

All animal experiments in this study complied with the "Guidelines for the Care and Use of Laboratory Animals at the University of South China" and were approved by the Animal Ethics Committee of the University of South China. Mice with orthotopic hepatic tumors were randomly assigned to two groups, each receiving an injection of 200 μL of 200 μM BTOGP NPs and BTOG-GPC3 NPs *via* the tail vein, respectively. NIR-II imaging was conducted at 0, 2, 4, 6, 8, and 10 hours. The mice were killed 10 hours later, and their organs and tumors were extracted for fluorescence imaging (FLI) and semi-quantitative fluorescence analysis (Whole-body imaging protocol: Excitation wavelength: 1064 nm; power density: 45 mW/cm^2; laser height: 40 cm; illumination area: 144 cm^2; long-pass filter: 1300 nm; exposure time: 1000 ms). Mice with orthotopic hepatic tumors were randomly assigned to two groups, each receiving an injection of 200 μL of 200 μM BTOG-GPC3 NPs *via* the tail vein, respectively, and 9-10 hours later, 200 μL of 200 μM BTOG-GPC3 NPs was injected via the tail vein again. Then the whole body vascular images (thorax, abdomen and legs) were rapidly acquired within 30 min. (Regional imaging protocol (thorax, abdomen, and hindlimbs):

Excitation wavelength: 1064 nm; power density: 45 mW/cm²; laser height: 30 cm; illumination area: 81 cm²; long-pass filter: 1300 nm; exposure time: 50 ms). *In vivo* NIR-II imaging: time-series analysis was conducted in tumor-bearing SCID mice, and BALB/c nude mice were utilized for high-resolution vascular imaging, as their hairless phenotype provides superior clarity for rapid angiography.

BTOG-GPC3 nanoparticles (200 μM) were loaded into capillary tubes and positioned beneath chicken breast tissue phantoms of varying thicknesses for tissue penetration assessment. NIR-II fluorescence imaging was performed using the following parameters: excitation wavelength, 1064 nm; power density, 45 mW/cm²; laser height, 30 cm; illumination area, 81 cm²; long-pass filter, 1500 nm; exposure time, 50 ms. Fluorescence signal intensities were quantified using ImageJ software analysis tools, with standardized regions of interest applied across all samples. Image preprocessing protocols, including noise reduction and background subtraction, were implemented prior to quantitative analysis. Quantified fluorescence data were systematically recorded and compiled for statistical evaluation.

1.13 Pharmacokinetics, biodistribution, and excretion of BTOG-GPC3 NPs

The healthy mice were injected with BTOG-GPC3 NPs (200 μM, 200 μL) intravenously to explore the pharmacokinetics, biodistribution, and excretion of BTOG-GPC3 NPs. The mice's blood and feces solutions were obtained for fluorescence analysis at regular intervals at 1500 nm to study the blood concentration, hepatic

excretion, and distribution of BTOG-GPC3 NPs (Their feces and all organs were homogenized and ground). The calculations for the pharmacokinetics, biodistribution, and excretion of BTOG-GPC3 NPs were performed according to several previous works [3b, 5]. Briefly, the fluorescence intensity of the solution from feces, blood, and all organs is multiplied by the volume of solution as the tissue or feces dose. Meanwhile, the background signal from all feces and organs of healthy mice was subtracted before calculation. And then the excretion can be calculated as S11, the curve was fitted with an exponential decay function:

$$Excretion = \frac{\sum_{1}^{n} V_n \times I_n}{V \times I} \qquad \text{(S11)}$$

1.14 Phototherapy of *in situ* tumor mice

SCID mice with in situ liver tumors were randomly assigned to the PBS group, FTTG-GPC3 NPs group, FEOG-GPC3 NPs group, FTOG-GPC3 NPs group, and BTOG-GPC3 NPs group. Following the nanoparticle administration into the tail vein, the tumor was irradiated with a 1064 nm laser (power density 1.0 W/cm^2, duration 10 minutes) 12 h after the nanoparticle injection. The mice's weight and tumor volume were evaluated every three days, with tumor volume determined using the formula S12-S13 to estimate treatment efficacy and Tumor inhibition.

$$Tumor\ volume\ (V) = \frac{W^2 \times L}{2} \qquad \text{(S12)}$$

$$Relative\ tumor\ (V) = \frac{V}{V0} \qquad \text{(S13)}$$

1.15 Pathologic analysis

After phototherapy, the tumor tissues from each experimental group were evaluated using hematoxylin-eosin (H&E), Ki-67, and TUNEL staining assays. Fourteen days post-treatment, the mice were euthanised, and whole blood and serum samples were collected for haematological analysis and assessment of liver and kidney function. Concurrently, significant organs and tumor tissues were excised, followed by histomorphological examination with H&E staining.

1.16 Ethical Approval

All procedures in the studies involving animals were performed in accordance with the ethical standards of the Institutional Animal Care and Use Committees (IACUC) of the University of South China (Hengyang, China). The assigned accreditation number of the laboratory is 2023027.

1.17 Statistical Analysis

Statistical analysis was conducted using two-tailed paired sample Student's t-tests in SPSS 26.0. Results are expressed as mean ± standard deviation (SD) from experiments performed in triplicate or more. The significance of the difference between the two groups was determined *via* Student's t-test. The significance of the difference between more than two groups was determined via an ANOVA-LSD *post hoc* test. * P<0.05. ** P<0.01. *** P<0.001. Error bars: mean ± SD (n = 3)

1.18 Synthetic procedures and characterization data for the molecular fluorophores.

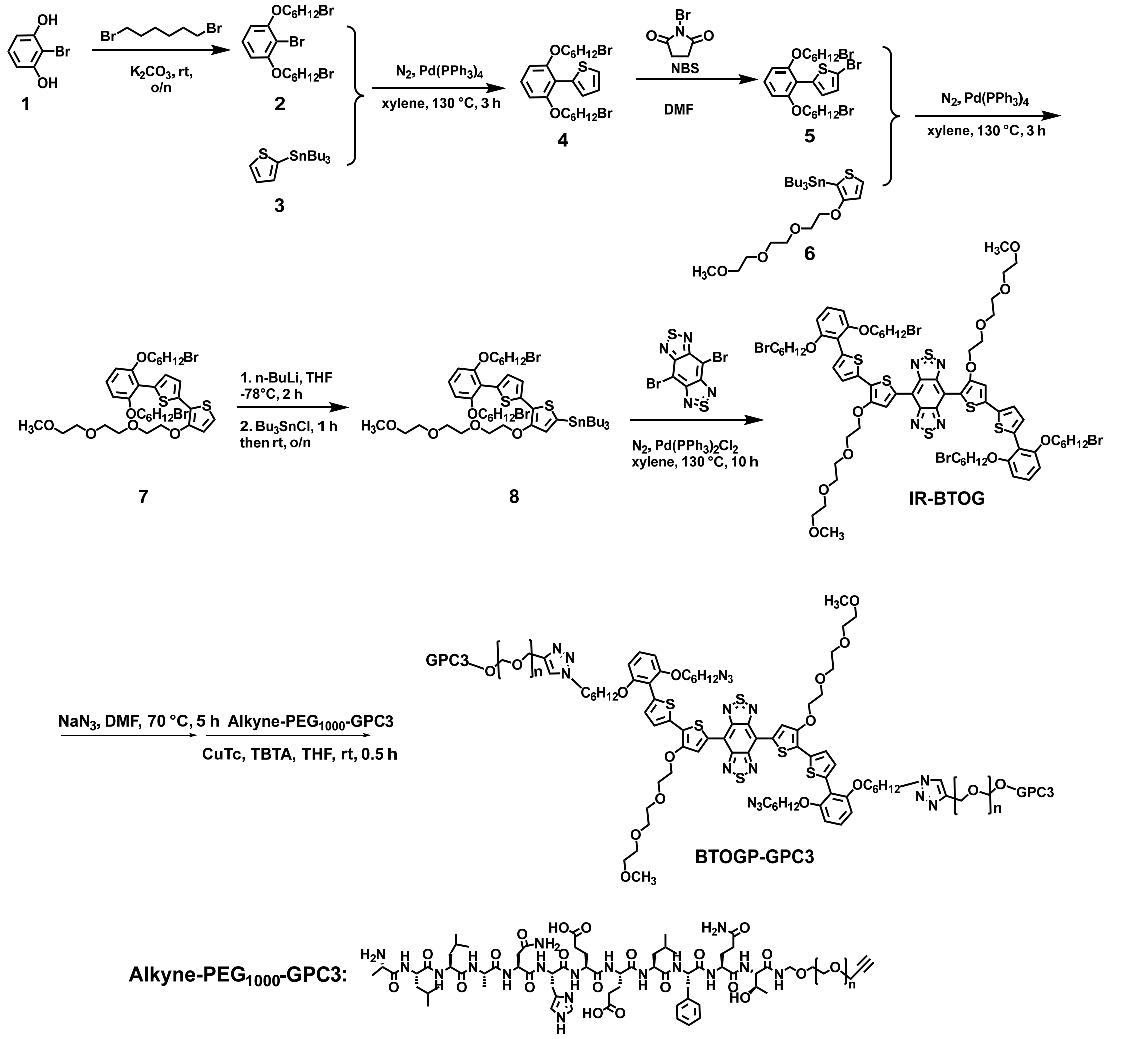

Scheme S1. Synthetic route of **BTOG-GPC3**

2-bromo-1,3-bis((6-bromohexyl)oxy)benzene (2) was synthesized according to the reference [6].

2-(2,6-bis((6-bromohexyl)oxy)phenyl)thiophene (4): To a solution of compound 2 (1.2 g, 2.35 mmol) and compound 3 (878 mg, 2.35 mmol) in toluene (15 mL) under nitrogen, $Pd(PPh_3)_4$ (269.2 mg, 0.233 mmol) was added. The mixture was stirred at 130 °C for 3 h. After cooling to room temperature, the mixture was poured into water, extracted twice with ethyl acetate, dried with $MgSO_4$, and evaporated *in vacuo*. The

crude product was subjected to column chromatography on silica gel to afford compound 4 as a white solid (1.08 g, 88.6%). ^{1}H NMR (500 MHz, $CDCl_3$) δ 7.40 (d, *J* = 3.5 Hz, 1H), 7.29 (d, *J* = 5.1 Hz, 1H), 7.10 (s, 1H), 7.02 (dd, *J* = 13.3, 9.3 Hz, 1H), 6.54 (d, *J* = 8.3 Hz, 1H), 6.45 (s, 1H), 3.95 – 3.92 (m, 4H), 3.34 (d, *J* = 8.0 Hz, 4H), 1.80 (dd, *J* = 14.3, 7.1 Hz, 8H), 1.48 – 1.46 (m, 4H), 1.39 – 1.37 (m, 4H). ^{13}C NMR (500 MHz, $CDCl_3$) δ 157.12, 156.73, 133.98, 128.70, 128.46, 128.04, 125.63, 125.05, 113.02, 105.77, 105.34, 102.17, 77.29, 77.04, 76.79, 74.15, 69.06, 68.79, 68.58, 33.90, 33.87, 32.70, 32.66, 28.97, 28.95, 27.87, 27.79, 25.32, 25.27, 13.63. HRMS (ESI) calcd for $C_{22}H_{31}O_2Br_2S_2^+$, ([M+H$^+$]) 519.0385, Found 519.0372.

5'-(2,6-bis((6-bromohexyl)oxy)phenyl)-3-(2-(2-(2-methoxyethoxy)ethoxy)ethoxy)-2,2'-bithiophene (7): To a solution of compound 4 (1.12 g, 2.16 mmol) in DMF (15 mL) under nitrogen, 1-bromopyrrolidine-2,5-dione (383.8 mg, 2.16 mmol) was added. The mixture was stirred at − 8 °C for 1 h. After cooling to room temperature for 4 h, the mixture was poured into water, extracted twice with ethyl acetate, dried with $MgSO_4$, and evaporated *in vacuo*. The crude product was subjected to column chromatography on silica gel to afford compound 5 as a yellow oil (1.01 g, 78.9%). To a solution of compound 5 (1 g, 1.67 mmol) and compound 6 (895 mg, 1.67 mmol) in toluene (15 mL) under nitrogen, $Pd(PPh_3)_4$ (193.49 mg, 0.167 mmol) was added. The mixture was stirred at 130 °C for 3 h. After cooling to room temperature, the mixture was poured into water, extracted twice with ethyl acetate, dried with $MgSO_4$, and evaporated *in vacuo*. The crude product was subjected to column chromatography on silica gel to

afford compound 7 as a brownish-black oil (0.81 g, 63.9%). ^1H NMR (500 MHz, $CDCl_3$) δ 7.34 – 7.20 (m, 2H), 7.08 (t, *J* = 8.3 Hz, 1H), 6.99 – 6.92 (m, 1H), 6.80 (t, *J* = 4.8 Hz, 1H), 6.53 (d, *J* = 3.7 Hz, 1H), 4.21 – 4.17 (m, 2H), 3.97 – 3.87 (m, 4H), 3.82 – 3.78 (m, 2H), 3.65 (dd, *J* = 5.8, 3.7 Hz, 2H), 3.58 – 3.53 (m, 4H), 3.45 – 3.42 (m, 2H), 3.30 – 3.25 (m, 7H), 1.74 (dd, *J* = 8.8, 7.0 Hz, 6H), 1.39 (dd, *J* = 9.3, 6.0 Hz, 6H), 1.21 – 1.17 (m, 4H). ^{13}C NMR (500 MHz, $CDCl_3$) δ 157.10, 157.01, 156.61, 154.44, 152.24, 152.17, 151.93, 136.56, 135.93, 134.55, 133.62, 133.21, 132.96, 132.31, 131.77, 130.98, 130.03, 129.81, 129.39, 129.06, 128.58, 128.49, 128.24, 126.96, 126.48, 126.42, 125.16, 124.49, 124.01, 122.51, 122.19, 122.05, 121.49, 121.06, 119.86, 119.74, 118.80, 118.33, 118.10, 117.07, 116.43, 113.68, 113.15, 109.49, 109.31, 105.47, 105.30, 105.25, 77.38, 77.13, 76.87, 72.86, 71.95, 71.92, 71.77, 71.15, 71.02, 70.97, 70.90, 70.82, 70.73, 70.69, 70.67, 70.63, 70.57, 70.05, 69.97, 69.84, 69.10, 68.91, 60.43, 59.04, 34.00, 33.96, 33.90, 33.88, 32.80, 32.71, 32.66, 32.53, 31.96, 31.47, 30.21, 29.89, 29.73, 29.05, 28.90, 27.88, 27.81, 26.60, 25.55, 25.44, 25.34, 25.10, 22.73, 21.10, 14.24, 14.18. HRMS (ESI) calcd for $C_{33}H_{47}O_6Br_2S_2^+$, ([M+H$^+$]) 763.1154, Found 763.1145.

IR-BTOG: To a solution of compound 7 (800 mg, 1.048 mmol) in THF (15.00 ml) at - 78 °C under nitrogen, *n*-BuLi solution (1.6 M in hexane, 806 μL, 1.29 mmol) was added dropwise. After stirring the mixture at this temperature for another 2.0 h, tributyltin chloride (420 mg, 1.29 mmol) was added to the solution. The reaction mixture was then slowly warmed to room temperature and stirred for 1.0 h. After that, the mixture was poured into water and extracted twice with ethyl acetate. The combined

organic phase was dried with $MgSO_4$ and evaporated *in vacuo* to afford compound 8. To a solution of compound 4,7-dibroMobenzo[1,2-c:4,5-c']bis([1,2,5]thiadiazole) BBTD (93.68 mg, 0.27 mmol) and compound 8 (944.5 mg, 0.81 mmol) in toluene (10 ml) under nitrogen, Pd $(PPh_3)_2Cl_2$ (56.04 mg, 0.048mmol) was added. The mixture was stirred at 130 °C for 10 h. After cooling to room temperature, the mixture was poured into water, extracted twice with ethyl acetate, dried with $MgSO_4$, and evaporated *in vacuo*. The crude product was subjected to column chromatography on silica gel to afford IR-BTOG as a brown solid (258.6 mg, 56.0%). ^{1}H NMR (500 MHz, $CDCl_3$) δ 7.69 (dd, *J* = 12.0, 7.1 Hz, 2H), 7.58 (dd, *J* = 13.7, 5.0 Hz, 2H), 7.50 (dd, *J* = 7.7, 2.7 Hz, 2H), 7.21 (dd, *J* = 8.3, 3.5 Hz, 3H), 6.65 (dd, *J* = 11.4, 8.4 Hz, 3H), 4.55 (s, 1H), 4.33 (s, 1H), 4.03 (ddd, *J* = 47.7, 27.1, 18.6 Hz, 12H), 3.82 – 3.51 (m, 18H), 3.40 (ddd, *J* = 14.9, 11.3, 9.4 Hz, 14H), 1.88 (d, *J* = 6.6 Hz, 8H), 1.53 – 1.47 (m, 8H), 1.33 – 1.28 (m, 8H), 0.92 – 0.85 (m, 8H). ^{13}C NMR (500 MHz, $CDCl_3$) δ 177.53, 162.58, 135.17, 134.80, 132.86, 132.15, 132.08, 132.04, 132.00, 131.98, 130.41, 129.56, 128.58, 128.49, 128.15, 127.90, 127.85, 127.35, 77.34, 77.08, 76.83, 75.59, 71.90, 71.75, 70.66, 69.88, 68.91, 60.21, 59.03, 57.78, 36.53, 33.89, 32.69, 31.46, 30.20, 29.63, 29.05, 27.74, 25.31. HRMS (ESI) calcd for $C_{72}H_{90}O_{12}Br_4N_4S_6^+$, ($[M]^-$) 1714.1577, Found 1714.1681. Optical parameters in water: ε (10^3L/mol·cm，1064 nm)：52.5, λex_{max} = 1051 nm, λem_{max} = 1303 nm, quantum yield is 0.0956% (QY data were quantified with IR-26 in DCM quantum yield is 0.05% [7]).

IR-BTOGP-GPC3 (BTOGP-GPC3): Compound **IR-BTOG** (100 mg, 0.058 mmol) and sodium azide (47 mg, 0.72 mmol) were dissolved in DMF (10 mL) and heated for 5 h at 70°C. After that, a large amount of water was added and stirred until all solids dissolved. Then, it was extracted twice with ethyl acetate, and the combined organic phase was dried with $MgSO_4$ and evaporated *in vacuo*. The crude product was subjected to flash column chromatography (DCM/MeOH = 5) on silica gel to afford a dark brown solid (86 mg, 0.055 mmol). The dark brown solid was dissolved in 5 mL THF and copper(I) thiophene-2-carboxylate (CuTc) (10 mg), alkyne-PEG_{1000}-GPC3 (159 mg), and tris[(1-benzyl-1H-1,2,3-triazol-4-yl) methyl] amine (TBTA) (5 mg) was added. The system was stirred at RT for 0.5 h and then filtered with diatomite. The solvent was evaporated *in a vacuum*. The crude product was subjected to column chromatography on silica gel with (DCM/MeOH = 5) and recrystallized by methyl tert-butyl ether to afford BTOGP-GPC3 as a dark brown solid (167.6 mg, 85.5%). ^{1}H NMR (500 MHz, $CDCl_3$) δ 7.60 (dd, *J* = 11.9, 7.6 Hz, 2H), 7.54 – 7.44 (m, 3H), 7.40 (t, *J* = 6.4 Hz, 4H), 7.11 (s, 3H), 6.56 (dd, *J* = 11.0, 8.7 Hz, 4H), 4.35 (d, *J* = 110.4 Hz, 3H), 4.02 – 3.23 (m, 168H), 1.81 (dd, *J* = 25.6, 19.2 Hz, 26H), 1.47 – 1.14 (m, 34H), 0.78 (dd, *J* = 9.0, 4.8 Hz, 8H). ^{13}C NMR (500 MHz, CDCl3) δ 132.15, 131.13, 128.48, 115.00, 105.47, 89.64, 77.31, 77.06, 76.80, 71.92, 70.57, 59.05, 33.99, 32.71, 29.03, 27.87, 25.45. Optical parameters in water: ε (10^3L/mol·cm，1064 nm)：45.4, λex_{max} = 1062 nm, λem_{max} = 1316 nm, quantum yield is 0.00736% (QY data were quantified with IR-26 in DCM quantum yield is 0.05% [7]).

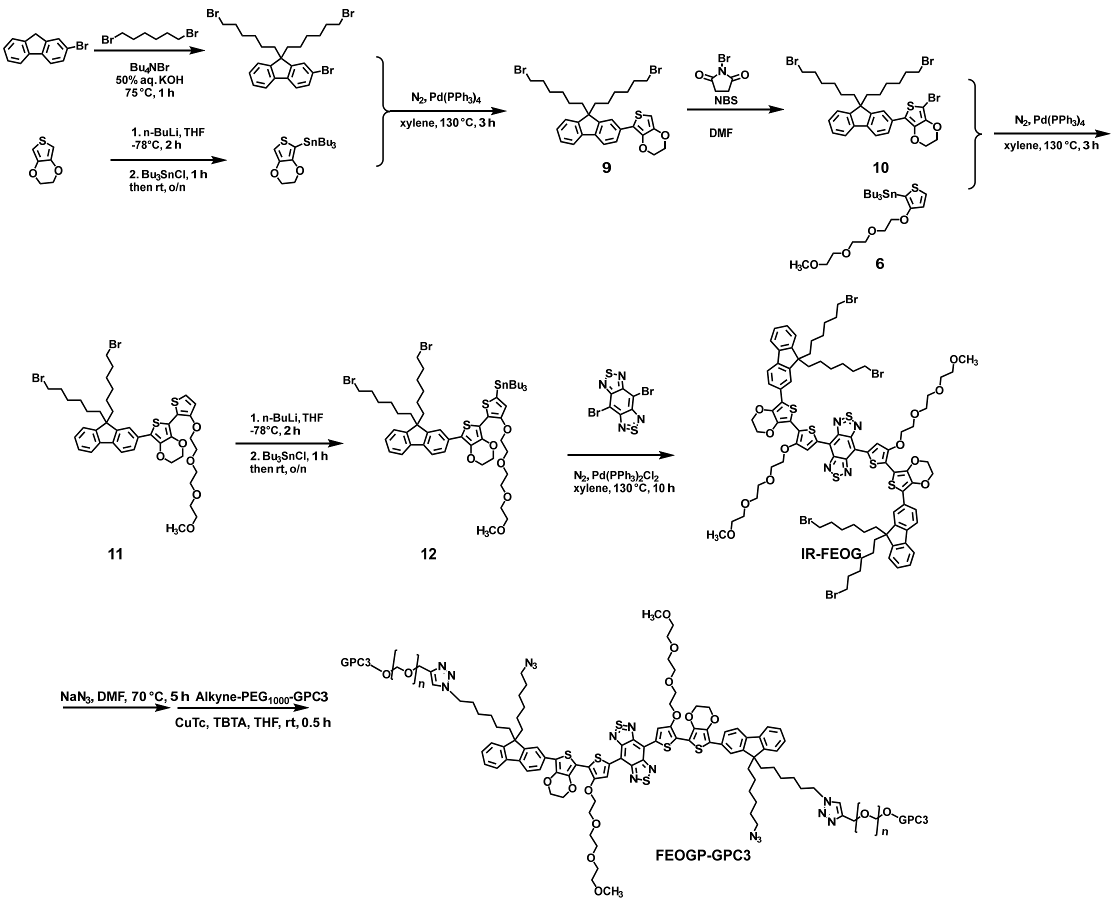

Scheme S1. Synthetic route of **FEOG-GPC3**

Compounds 9, 10, and 11 were synthesised according to the method described in reference [9].

5-(9,9-bis(6-bromohexyl)-9H-fluoren-2-yl)-7-(3-(2-(2-(2-methoxyethoxy)ethoxy)ethoxy)thiophen-2-yl)-2,3-dihydrothieno[3,4-b][1,4]dioxine (11): To a solution of compound **9** (1 g, 1.58 mmol) in DMF (12 mL) under nitrogen, 1-bromopyrrolidine-2,5-dione (281.4 mg, 1.58 mmol) was added. The mixture was stirred at − 8 °C for 1 h. After cooling to room temperature for 4 h, the mixture was poured into water, extracted twice with ethyl acetate, dried with $MgSO_4$, and evaporated *in vacuo*. The crude product was subjected to column chromatography on silica gel to afford compound 10 as a light yellow oil (0.93 g, 83.0%). To a solution of compound 6

(715.6 mg, 1.33 mmol) and compound 10 (945 mg, 1.33 mmol) in toluene (15 mL) under nitrogen, $Pd(PPh_3)_4$ (153.6 mg, 0.176 mmol) was added. The mixture was stirred at 130 °C for 3 h. After cooling to room temperature, the mixture was poured into water, extracted twice with ethyl acetate, dried with $MgSO_4$, and evaporated *in vacuo*. The crude product was subjected to column chromatography on silica gel to afford compound 11 as a brownish-black oil (0.87 g, 75%). ^{1}H NMR (500 MHz, $CDCl_3$) δ 7.75 – 7.73 (m, 2H), 7.37 (dd, *J* = 10.9, 4.7 Hz, 2H), 7.23 – 7.19 (m, 1H), 7.11 (dd, *J* = 6.9, 1.5 Hz, 1H), 6.89 – 6.85 (m, 2H), 6.26 (d, *J* = 2.2 Hz, 1H), 4.37 – 4.33 (m, 4H), 4.27 – 4.23 (m, 4H), 3.94 – 3.90 (m, 2H), 3.78 (dd, *J* = 7.2, 3.1 Hz, 2H), 3.68 – 3.64 (m, 4H), 3.55 – 3.51 (m, 4H), 3.36 (d, *J* = 1.9 Hz, 3H), 2.34 (s, 2H), 2.06 – 1.97 (m, 2H), 1.71 – 1.60 (m, 4H), 1.48 (dd, *J* = 14.9, 7.4 Hz, 4H), 1.27 (d, *J* = 1.7 Hz, 2H), 0.96 (td, *J* = 7.3, 2.0 Hz, 6H). ^{13}C NMR (500 MHz, $CDCl_3$) δ 152.39, 152.19, 141.11, 137.73, 137.04, 136.57, 133.32, 128.60, 126.23, 125.89, 122.41, 122.06, 119.88, 116.97, 116.88, 114.49, 114.01, 113.98, 110.58, 108.93, 97.30, 77.42, 77.17, 76.91, 71.93, 71.90, 71.57, 71.45, 70.96, 70.92, 70.70, 70.55, 70.05, 70.01, 65.02, 64.84, 64.74, 64.63, 60.40, 59.03, 59.01, 27.32, 21.83, 21.07, 16.80, 14.22, 13.25. HRMS (ESI) calcd for $C_{42}H_{53}O_6Br_2S_2^+$, ([M+H$^+$]) 877.1624, Found 877.1601.

IR-FEOG: To a solution of compound 11 (622 mg, 0.71 mmol) in THF (10.00 ml) at - 78 °C under nitrogen, *n*-BuLi solution (1.6 M in hexane, 530 μL, 0.85 mmol) was added dropwise. After stirring the mixture at this temperature for another 2.0 h, tributyltin chloride (267 μL, 0.85 mmol) was added to the solution. The reaction

mixture was then slowly warmed to room temperature and stirred for 1.0 h. After that, the mixture was poured into water and extracted twice with ethyl acetate. The combined organic phase was dried with $MgSO_4$ and evaporated *in vacuo* without further purification to afford compound 12. To a solution of compound BBTD (66.91 mg, 0.19 mmol) and compound 13(664.6 mg, 0.57 mmol) in toluene (10 ml) under nitrogen, Pd $(PPh_3)_2Cl_2$ (40.03 mg, 0.057 mmol) was added. The mixture was stirred at 130 °C for 10 h. After cooling to room temperature, the mixture was poured into water, extracted twice with ethyl acetate, dried with $MgSO_4$, and evaporated *in vacuo*. The crude product was subjected to column chromatography on silica gel to afford **IR-FEOG** as a brown solid (216.49 mg, 58.6%). ^{1}H NMR (500 MHz, $CDCl_3$) δ 7.73 (s, 2H), 7.46 (t, J = 19.2 Hz, 8H), 7.22 (t, J = 6.8 Hz, 3H), 7.08 (s, 3H), 4.47 (s, 1H), 4.29 (d, J = 4.5 Hz, 2H), 3.91 (s, 2H), 3.79 – 3.49 (m, 32H), 3.47 – 3.30 (m, 9H), 2.36 (t, J = 7.1 Hz, 8H), 1.81 – 1.60 (m, 12H), 1.50 (dd, J = 14.9, 7.4 Hz, 8H), 0.98 (t, J = 7.3 Hz, 12H).^{13}C NMR (500 MHz, $CDCl_3$) δ 135.13, 134.46, 134.07, 134.00, 133.05, 132.08, 131.12, 130.39, 130.20, 129.83, 129.76, 128.88, 128.58, 128.48, 128.28, 127.99, 127.55, 127.51, 127.47, 127.13, 116.86, 79.51, 77.30, 77.04, 76.79, 72.57, 71.88, 71.72, 71.48, 71.19, 71.01, 70.87, 70.68, 70.54, 70.32, 70.07, 69.26, 64.92, 64.70, 64.36, 60.62, 59.02, 52.67, 35.91, 34.51, 31.92, 31.36, 29.72, 29.34, 29.26, 29.14, 28.79, 27.19, 25.99, 25.54, 24.85, 22.71, 18.32, 14.15, 14.09, 13.64, -6.40. HRMS (ESI) calcd for $C_{90}H_{102}Br_4N_4O_{12}S_6^+$, ([M $^+$]) 1940.2526, Found 1940.2362. Optical parameters in water: ε (10^3L/mol·cm,

1064 nm)：30.8, λex_{max} = 944 nm, λem_{max} = 1140 nm, quantum yield is 0.1353% (QY data were quantified with IR-26 in DCM quantum yield is 0.05% [7]).

IR-FEOGP-GPC3 (FEOGP-GPC3): Compound **IR-FEOG** (113 mg, 0.058 mmol) and sodium azide (47 mg, 0.72 mmol) were dissolved in DMF (10 mL) and heated for 5 h at 70°C. After that, a large amount of water was added and stirred until all solids dissolved. Then, it was extracted twice with ethyl acetate, and the combined organic phase was dried with $MgSO_4$ and evaporated *in vacuo*. The crude product was subjected to flash column chromatography (DCM/MeOH = 5) on silica gel to afford a dark brown solid (93 mg, 0.050 mmol). The dark brown solid was dissolved in 5 mL THF, and copper(I) thiophene-2-carboxylate (CuTc) (10 mg), alkyne-PEG_{1000}-GPC3 (150 mg), and tris[(1-benzyl-1H-1,2,3-triazol-4-yl) methyl] amine (TBTA) (5 mg) were added. The system was stirred at RT for 0.5 h and then filtered with diatomite. The solvent was evaporated *in a vacuum*. The crude product was subjected to column chromatography on silica gel with (PE/EA = 1) and recrystallized by methyl tert-butyl ether to afford **FEOGP-GPC3** as a dark brown solid (146.2 mg, 77.20%). ^{1}H NMR (500 MHz, $CDCl_3$) δ 7.40 (d, *J* = 3.9 Hz, 14H), 7.15 (s, 2H), 7.01 (s, 5H), 4.08 – 3.19 (m, 140H), 1.98 (s, 6H), 1.73 (s, 12H), 1.56 (dd, *J* = 14.6, 7.5 Hz, 6H), 1.23 (dd, *J* = 20.6, 9.2 Hz, 30H).^{13}C NMR (500 MHz, $CDCl_3$) δ 135.13, 134.57, 134.06, 134.00, 133.45, 132.15, 132.07, 131.77, 130.48, 130.20, 130.04, 129.88, 129.76, 129.20, 128.59, 128.53, 128.49, 128.18, 127.99, 127.56, 127.52, 127.48, 122.62, 116.87, 90.92, 77.31, 77.05, 76.80, 75.56, 71.88, 71.64, 71.48, 71.38, 71.01, 70.83, 70.68, 70.57, 70.54, 70.32, 70.07, 66.66,

64.92, 59.38, 59.02, 56.25, 35.91, 35.46, 31.93, 30.80, 29.72, 29.55, 29.35, 29.26, 29.14, 28.24, 27.24, 26.52, 25.54, 24.39, 22.71, 17.28, 14.16, -15.03. Optical parameters in water: ε (10^3L/mol·cm，1064 nm)：33.4, λex_{max} = 952 nm, λem_{max} = 1151 nm, quantum yield is 0.0106% (QY data were quantified with IR-26 in DCM quantum yield is 0.05% [7]).

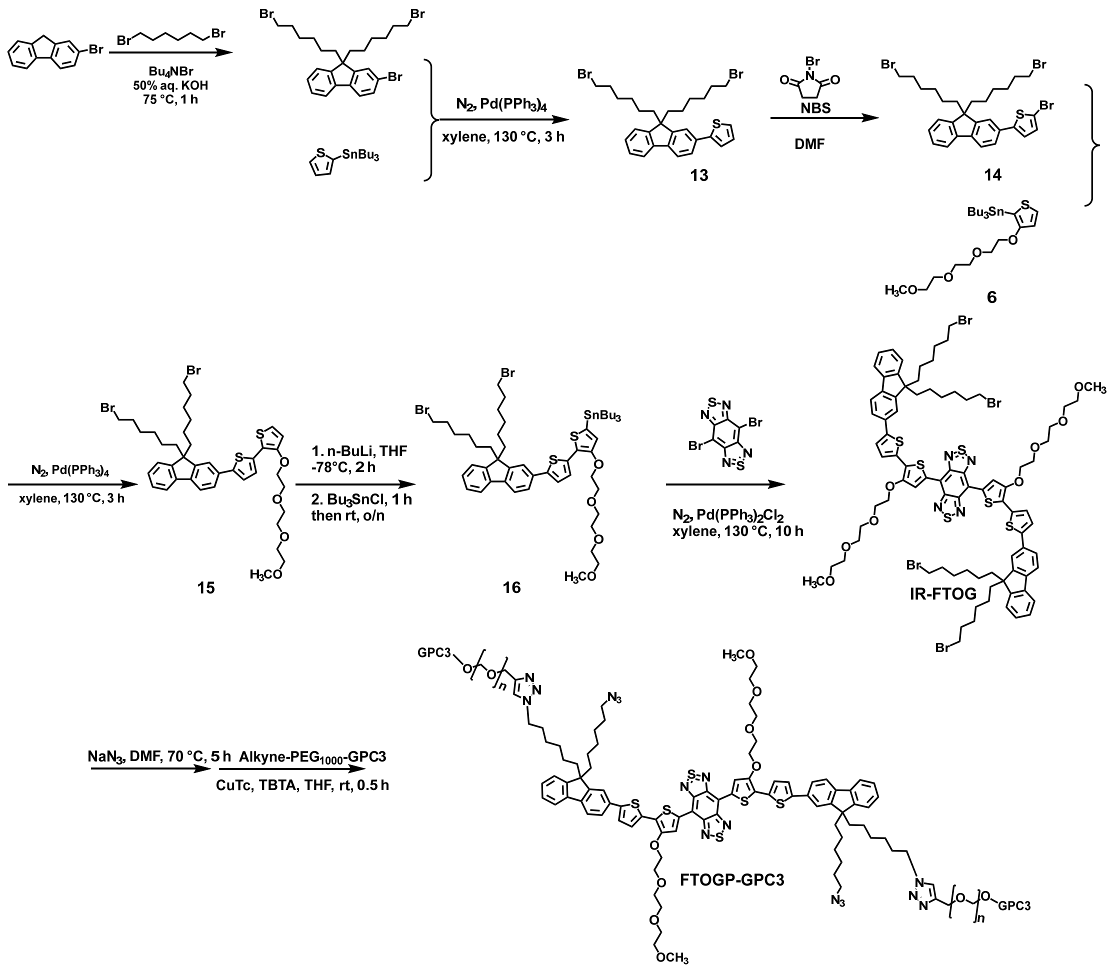

Scheme S3. Synthetic route of **FTOG-GPC3**

2-bromo-9,9-bis(6-bromohexyl)-9H-fluorene (13) was synthesized according to the reference. [9]

5'-(2,6-bis((6-bromohexyl)oxy)phenyl)-3-(2-(2-(2-methoxyethoxy)ethoxy)ethoxy)-2,2'-bithiophene (15): To a solution of compound **13** (1 g, 1.74 mmol) in DMF (15 mL)

under nitrogen, 1-bromopyrrolidine-2,5-dione (309.83 mg, 1.74 mmol) was added. The mixture was stirred at − 8 °C for 1 h. After cooling to room temperature for 4 h, the mixture was poured into water, extracted twice with ethyl acetate, dried with $MgSO_4$, and evaporated *in vacuo*. The crude product was subjected to column chromatography on silica gel to afford compound **14** as a light yellow oil (903 mg, 79.9%). To a solution of compound **14** (1.08 g, 1.67 mmol) and compound **6** (896 mg, 1.67 mmol) in toluene (15 mL) under nitrogen, $Pd(PPh_3)_4$ (193.49 mg, 0.167 mmol) was added. The mixture was stirred at 130 °C for 3 h. After cooling to room temperature, the mixture was poured into water, extracted twice with ethyl acetate, dried with $MgSO_4$, and evaporated *in vacuo*. The crude product was subjected to column chromatography on silica gel to afford compound **15** as a Brownish-black oil (1.16 g, 84.7%). ^{1}H NMR (500 MHz, $CDCl_3$) δ 7.61 (dd, J = 7.0, 4.8 Hz, 2H), 7.54 – 7.42 (m, 3H), 7.28 – 7.21 (m, 4H), 7.04 – 6.97 (m, 1H), 6.84 (t, J = 6.8 Hz, 1H), 4.24 (dd, J = 10.2, 5.5 Hz, 2H), 3.91 – 3.79 (m, 2H), 3.73 – 3.66 (m, 2H), 3.65 – 3.55 (m, 4H), 3.51 – 3.42 (m, 2H), 3.42 – 3.25 (m, 4H), 3.24 – 3.14 (m, 3H), 1.93 (t, J = 8.1 Hz, 4H), 1.57 (t, J = 7.0 Hz, 4H), 1.26 (s, 4H), 1.10 – 0.99 (m, 4H), 0.85 (t, J = 7.3 Hz, 4H). ^{13}C NMR (500 MHz, $CDCl_3$) δ 152.37, 151.62, 151.12, 150.69, 150.46, 147.27, 142.64, 142.14, 140.72, 140.47, 135.43, 134.41, 133.69, 133.39, 132.33, 132.16, 132.08, 131.74, 128.58, 127.50, 127.17, 126.99, 125.06, 124.59, 124.07, 123.32, 123.00, 122.78, 122.50, 121.58, 120.46, 120.14, 119.98, 119.74, 119.53, 118.88, 118.16, 116.70, 116.48, 111.91, 79.34, 77.31, 77.06, 76.80, 75.27, 71.95, 71.81, 71.33, 71.16, 70.99, 70.75, 70.62, 70.51, 70.09, 69.32,

59.07, 57.74, 55.04, 54.39, 45.11, 40.59, 40.30, 39.32, 34.01, 33.68, 32.65, 32.47, 32.07, 31.46, 31.19, 30.21, 29.73, 29.07, 28.70, 27.78, 27.56, 26.83, 26.62, 26.49, 26.09, 23.55, 15.46, 13.64, 0.92. HRMS (ESI) calcd for $C_{40}H_{51}O_4Br_2S_2^+$, ([M+H$^+$]) 819.1569, Found 819.1561.

IR-FTOG: To a solution of compound 15 (858 mg, 1.048 mmol) in THF (15.00 ml) at - 78 °C under nitrogen, *n*-BuLi solution (1.6 M in hexane, 806 μL, 1.29 mmol) was added dropwise. After stirring the mixture at this temperature for another 2.0 h, tributyltin chloride (420 mg, 1.29 mmol) was added to the solution. The reaction mixture was then slowly warmed to room temperature and stirred for 1.0 h. After that, the mixture was poured into water and extracted twice with ethyl acetate. The combined organic phase was dried with $MgSO_4$ and evaporated *in vacuo* to afford compound 16. To a solution of compound BBTD (66.91 mg, 0.19 mmol) and compound 17 (630.42 mg, 0.57 mmol) in toluene (10 ml) under nitrogen, Pd $(PPh_3)_2Cl_2$ (40.03 mg, 0.057 mmol) was added. The mixture was stirred at 130 °C for 10 h. After cooling to room temperature, the mixture was poured into water, extracted twice with ethyl acetate, dried with $MgSO_4$, and evaporated *in vacuo*. The crude product was subjected to column chromatography on silica gel to afford **IR-FTOG** as a brown solid (216.49 mg, 58.6%). ^{1}H NMR (500 MHz, $CDCl_3$) δ 7.74 – 7.71 (m, 4H), 7.62 (d, J = 7.0 Hz, 2H), 7.40 – 7.32 (m, 12H), 7.02 (s, 2H), 4.40 – 4.37 (m, 3H), 4.00 – 3.98 (m, 3H), 3.84 – 3.82 (m, 3H), 3.74 (s, 3H), 3.69 (s, 3H), 3.57 (s, 3H), 3.38 (d, J = 9.3 Hz, 8H), 3.30 (dd, J = 9.1, 4.5 Hz, 8H), 2.04 (d, J = 8.1 Hz, 10H), 1.64 (d, J = 21.3 Hz, 14H), 1.22 (s,

10H), 0.74 – 0.65 (m, 10H). ^{13}C NMR (500 MHz, $CDCl_3$) δ 177.22, 168.27, 157.60, 157.50, 157.11, 156.58, 152.61, 152.51, 151.92, 142.00, 135.18, 135.02, 134.55, 134.15, 133.39, 131.80, 130.41, 129.19, 129.06, 128.49, 128.24, 127.90, 127.23, 126.97, 126.91, 126.42, 125.34, 124.67, 122.96, 122.17, 121.60, 121.47, 121.41, 121.05, 120.85, 119.62, 119.18, 118.80, 118.44, 118.33, 115.41, 113.18, 106.73, 105.48, 99.33, 97.51, 86.51, 77.34, 77.08, 76.83, 74.21, 71.93, 71.51, 71.15, 71.11, 70.90, 70.84, 70.79, 70.68, 70.66, 70.58, 70.19, 70.04, 69.97, 69.70, 69.57, 68.91, 61.68, 59.06, 57.14, 45.08, 44.06, 38.80, 35.19, 33.96, 33.73, 32.70, 32.53, 31.67, 31.46, 31.04, 30.21, 30.15, 29.59, 29.04, 28.33, 28.18, 27.87, 27.06, 26.84, 26.74, 26.59, 26.01, 25.54, 25.43, 20.00, 13.64, -11.55. HRMS (ESI) calcd for $C_{86}H_{99}Br_4N_4O_8S_6^+$, ($[M^+]$) 1829.2454, Found 1829.2542. Optical parameters in water: ε (10^3L/mol·cm，1064 nm)：34.3, λex_{max} = 1032 nm, λem_{max} = 1242 nm, quantum yield is 0.1731% (QY data were quantified with IR-26 in DCM quantum yield is 0.05% [7]).

IR-FTOGP-GPC3 (FTOGP-GPC3): Compound **IR-FTOG** (113 mg, 0.058 mmol) and sodium azide (47 mg, 0.72 mmol) were dissolved in DMF (10 mL) and heated for 5 h at 70°C. After that, a large amount of water was added and stirred until all solids dissolved. Then, it was extracted twice with ethyl acetate, and the combined organic phase was dried with $MgSO_4$ and evaporated *in vacuo*. The crude product was subjected to flash column chromatography (DCM/MeOH = 5) on silica gel to afford a dark brown solid (93 mg, 0.055 mmol). The dark brown solid was dissolved in 5 mL THF and copper(I) thiophene-2-carboxylate (CuTc) (10 mg), alkyne-PEG_{1000}-GPC3 (150 mg),

and tris[(1-benzyl-1H-1,2,3-triazol-4-yl) methyl] amine (TBTA) (5 mg) was added. The system was stirred at RT for 0.5 h and then filtered with diatomite. The solvent was evaporated *in a vacuum*. The crude product was subjected to column chromatography on silica gel (PE/EA = 1) and recrystallized by methyl tert-butyl ether to afford **FTOGP-GPC3** as a dark brown solid (155.6 mg, 76.7%). ^{1}H NMR (500 MHz, $CDCl_3$) δ 7.40 (d, J = 3.9 Hz, 14H), 7.15 (s, 2H), 7.01 (s, 5H), 4.08 – 3.19 (m, 140H), 1.98 (s, 6H), 1.73 (s, 12H), 1.56 (dd, J = 14.6, 7.5 Hz, 6H), 1.23 (dd, J = 20.6, 9.2 Hz, 30H).^{13}C NMR (500 MHz, $CDCl_3$) δ 135.13, 134.57, 134.06, 134.00, 133.45, 132.15, 132.07, 131.77, 130.48, 130.20, 130.04, 129.88, 129.76, 129.20, 128.59, 128.53, 128.49, 128.18, 127.99, 127.56, 127.52, 127.48, 122.62, 116.87, 90.92, 77.31, 77.05, 76.80, 75.56, 71.88, 71.64, 71.48, 71.38, 71.01, 70.83, 70.68, 70.57, 70.54, 70.32, 70.07, 66.66, 64.92, 59.38, 59.02, 56.25, 35.91, 35.46, 31.93, 30.80, 29.72, 29.55, 29.35, 29.26, 29.14, 28.24, 27.24, 26.52, 25.54, 24.39, 22.71, 17.28, 14.16, -15.03. Optical parameters in water: ε (10^3L/mol·cm，1064 nm)：39.9, λex_{max} = 1034 nm, λem_{max} = 1250 nm, quantum yield is 0.0126% (QY data were quantified with IR-26 in DCM quantum yield is 0.05% [7]).

2. Supplementary Figures

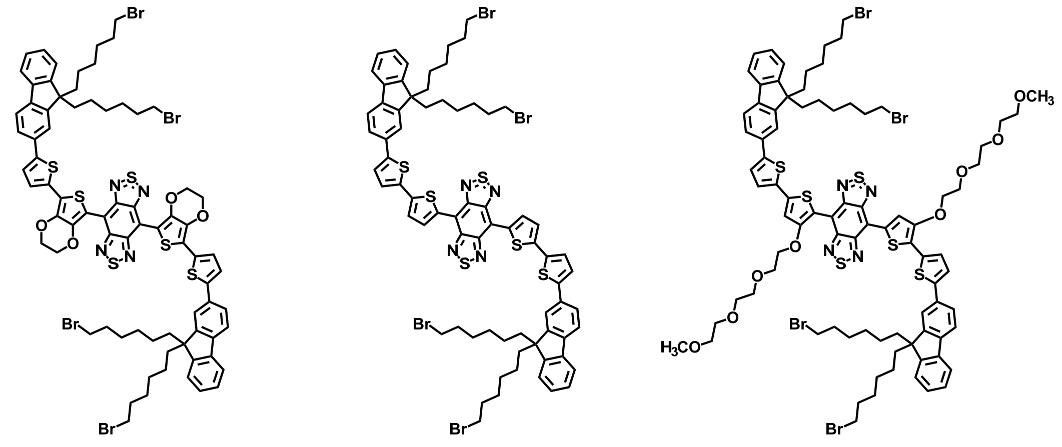

IR-FTE IR-FTT IR-FTG

***J. Am. Chem. Soc*, 2018, 140, 1715-1724**

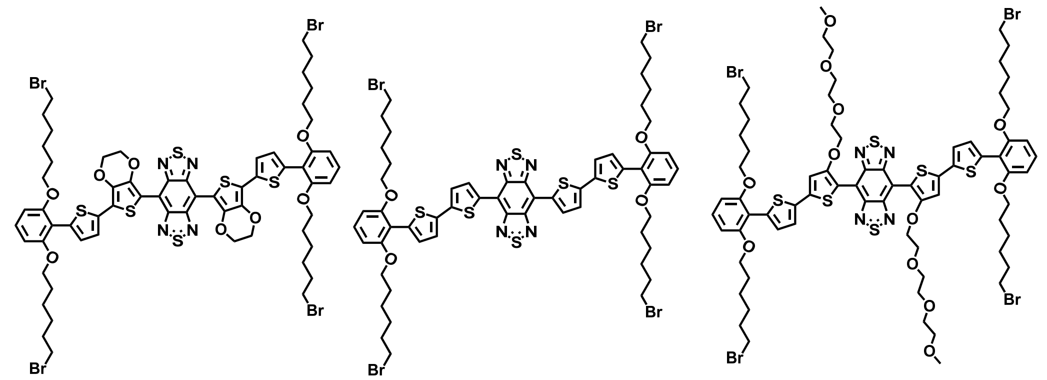

IR-BTE (IR-E1) IR-BTT (IR-T1) IR-BTG

***Adv. Mater*, 2016. 28. 6872**

***Adv.Fun. Mater*, 2018. 28. 1804956**

***Chem. Sci*, 2019. 210, 326-332**

***Sci China Chem.* 2024, 67, 2767-2774.**

Figure S1. The NIR-II D-A-D type organic molecule was previously designed and synthesized by our research group[8].

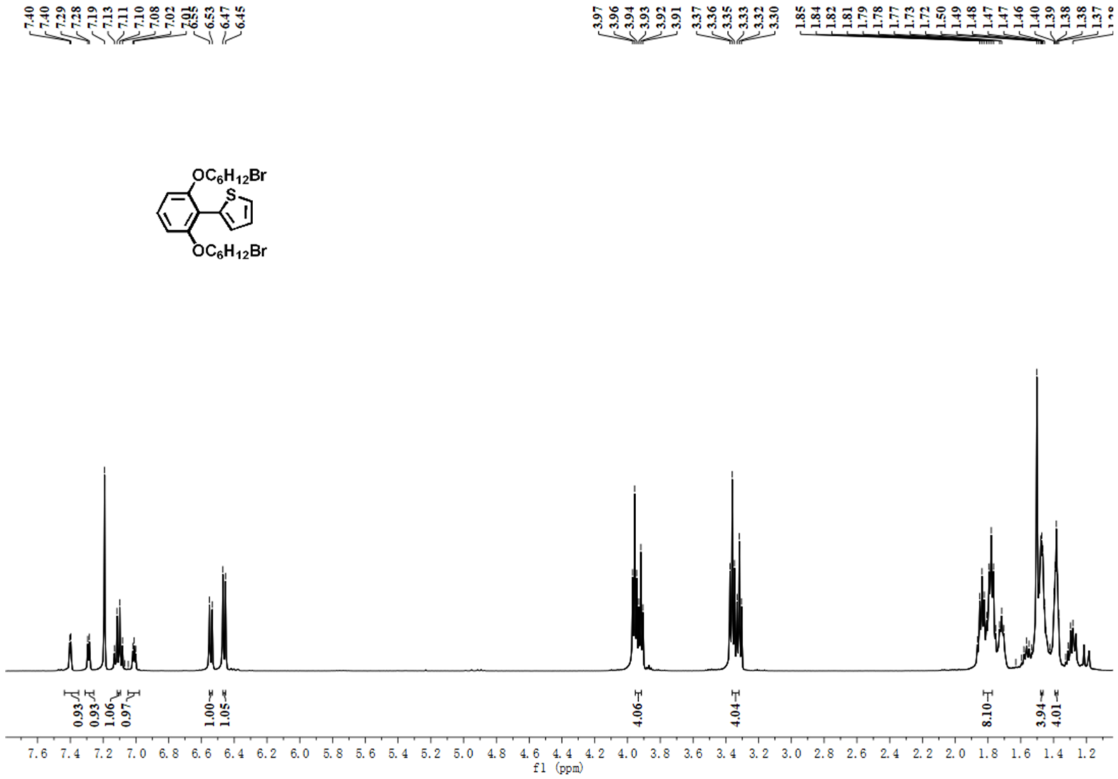

Figure S2. ^{1}H NMR of compound 4

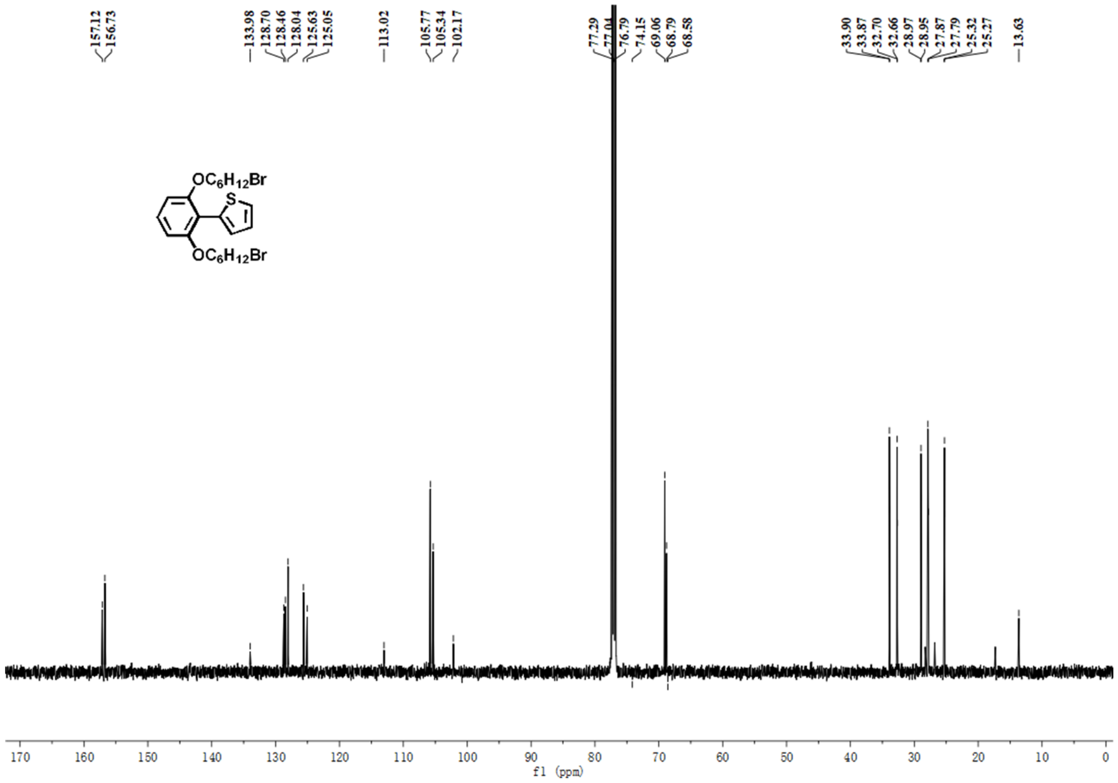

Figure S3. ^{13}C NMR of compound 4

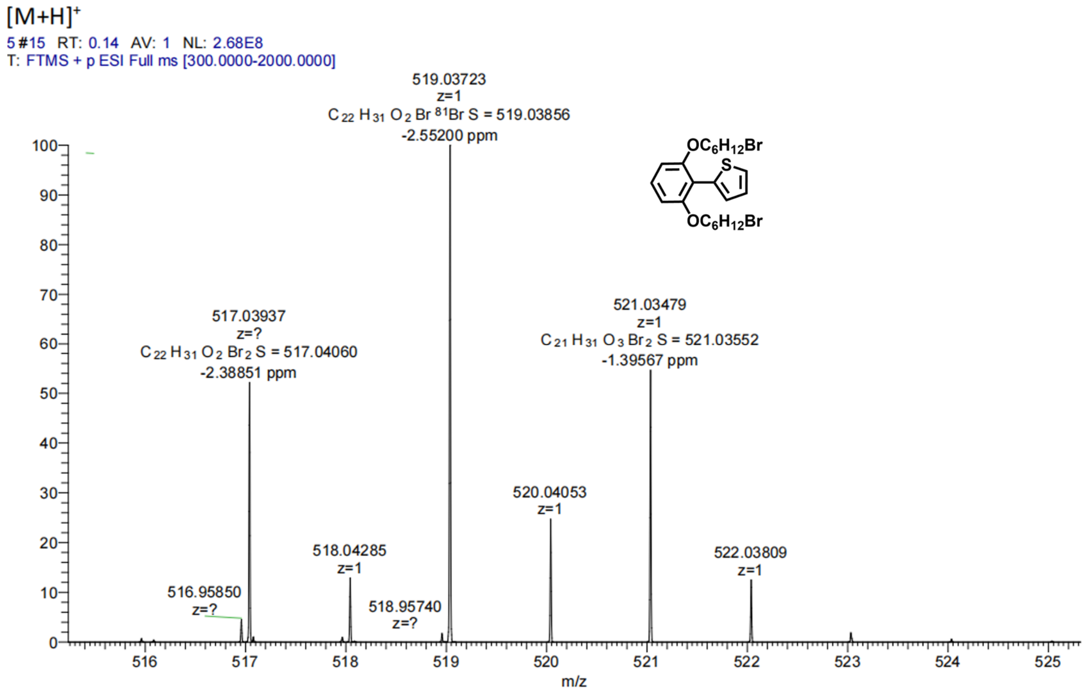

Figure S4. HRMS of compound 4.

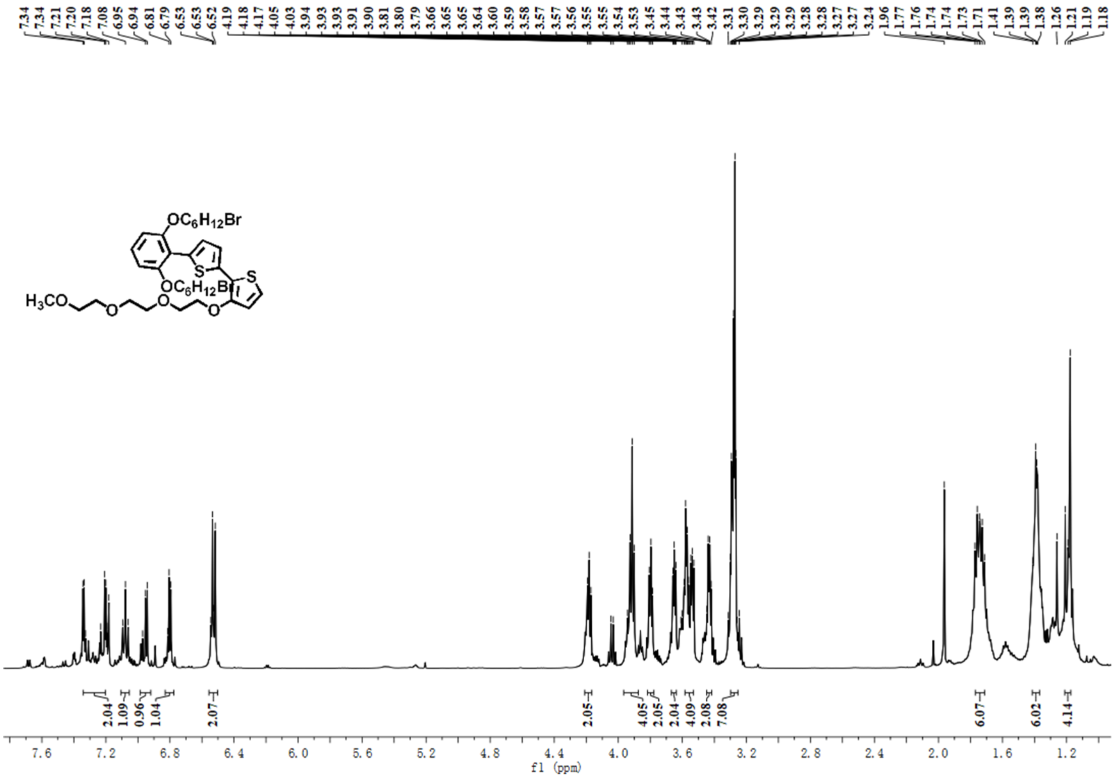

Figure S5. ^{1}H NMR of compound 7.

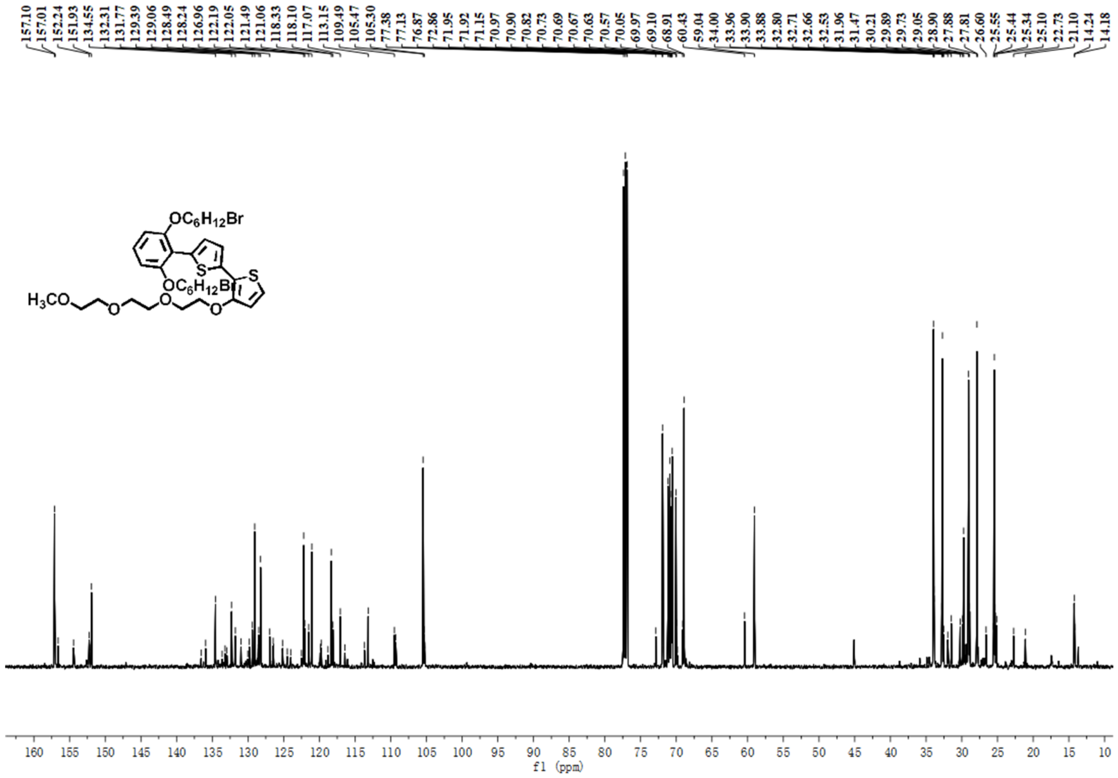

Figure S6. ^{13}C NMR of compound 7.

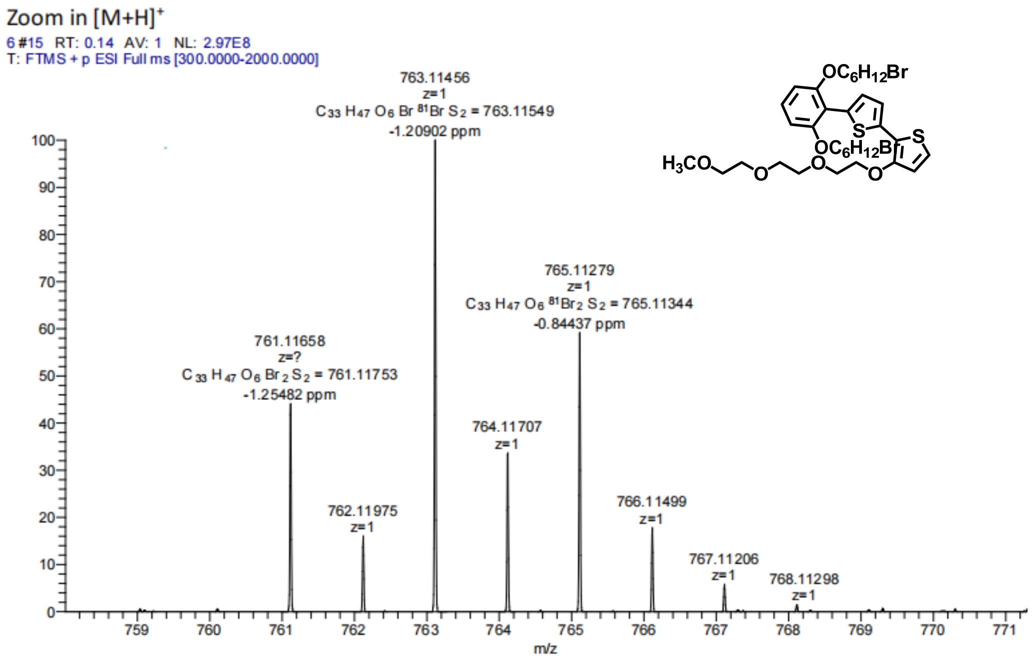

Figure S7. HRMS of compound 7.

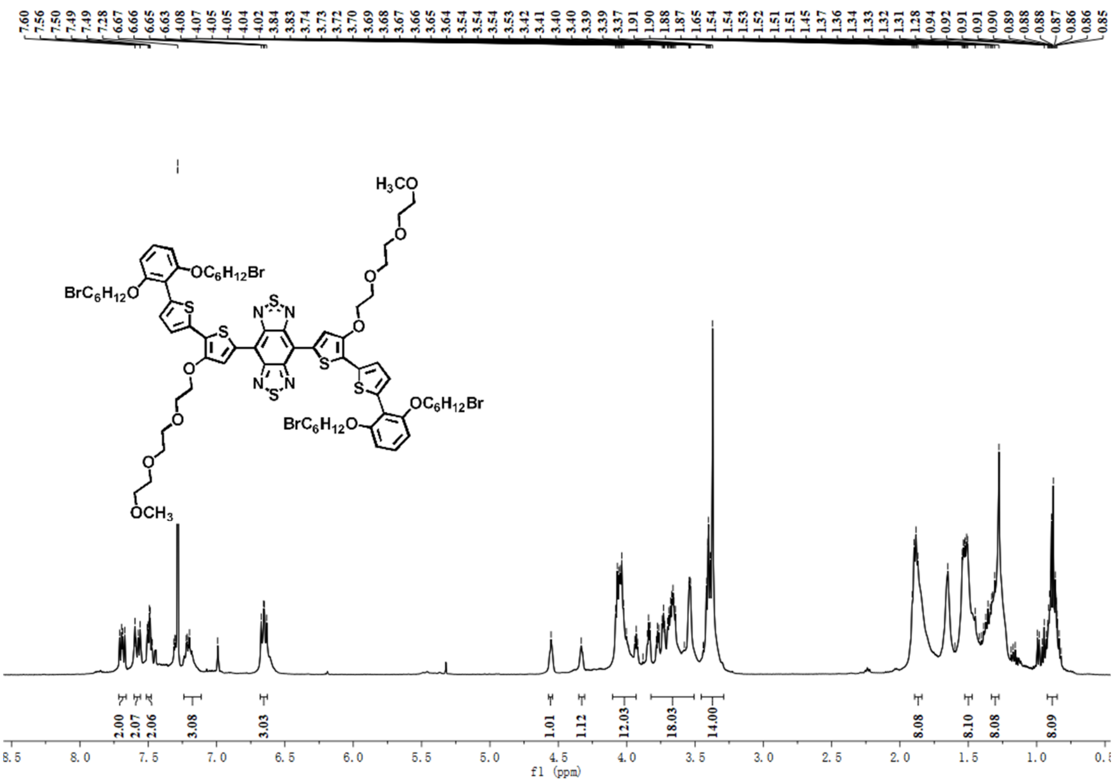

Figure S8. ^{1}H NMR of compound **IR-BTOG**.

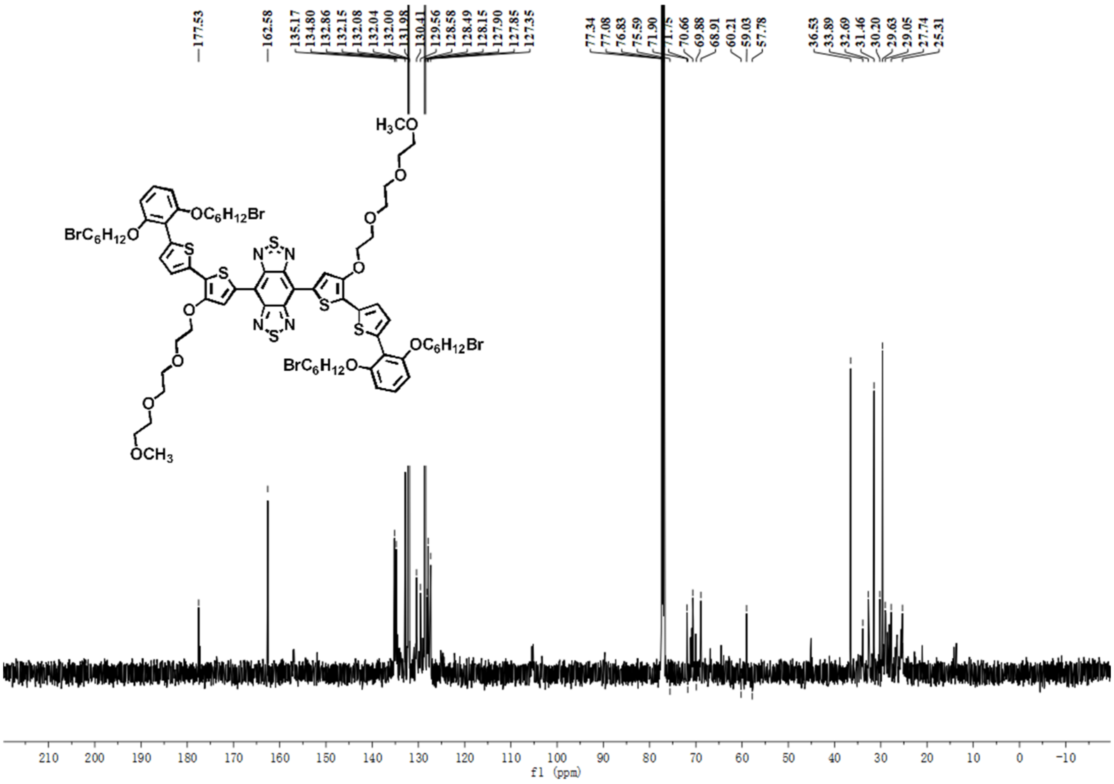

Figure S9. ^{13}C NMR of compound **IR-BTOG**.

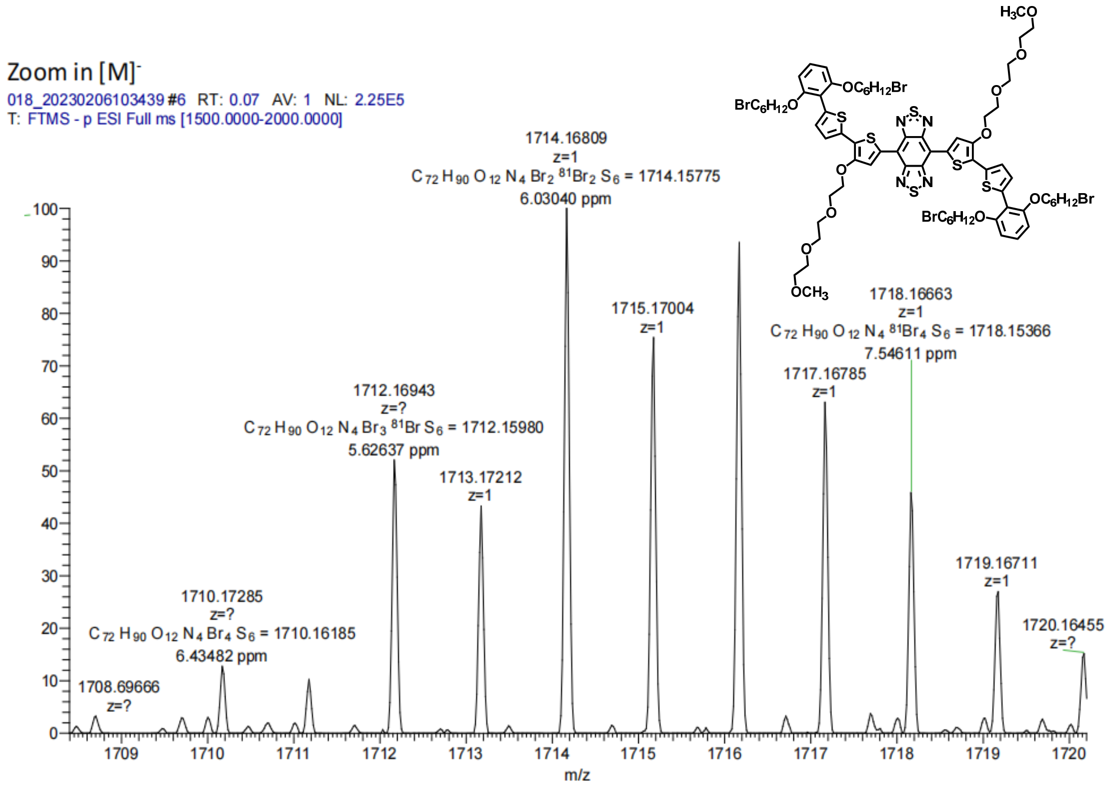

Figure S10. HRMS of compound **IR-BTOG**.

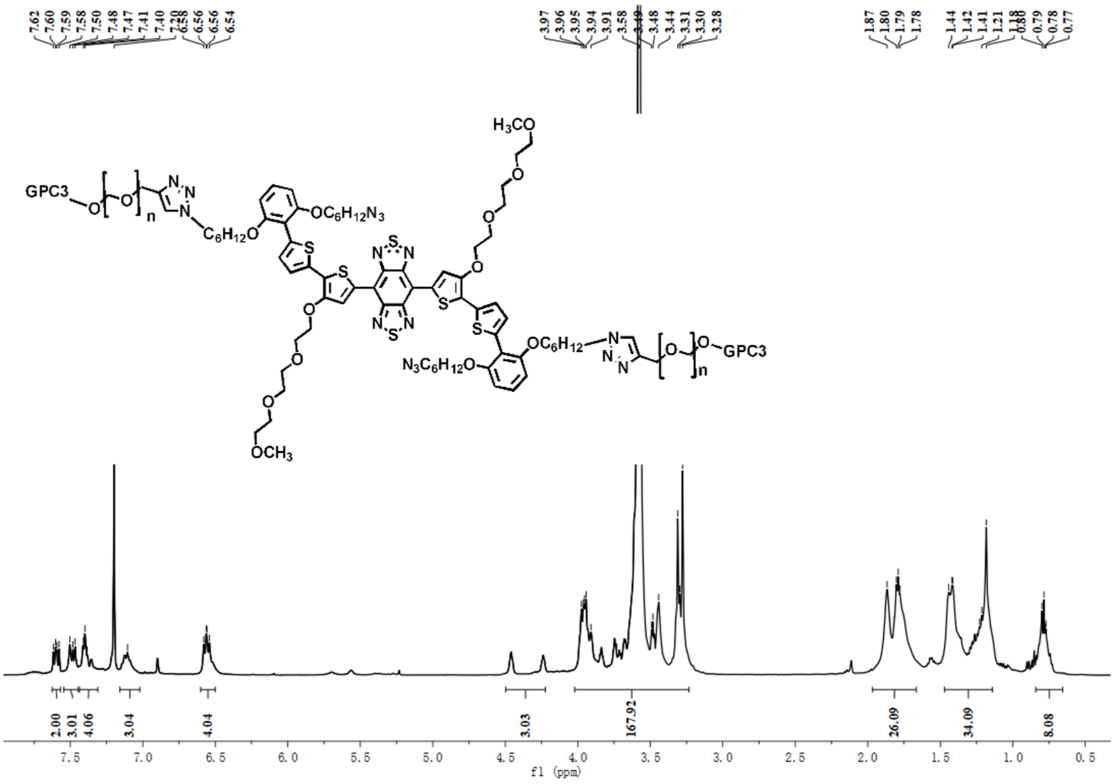

Figure S11. ^{1}H NMR of compound **BTOGP-GPC3**.

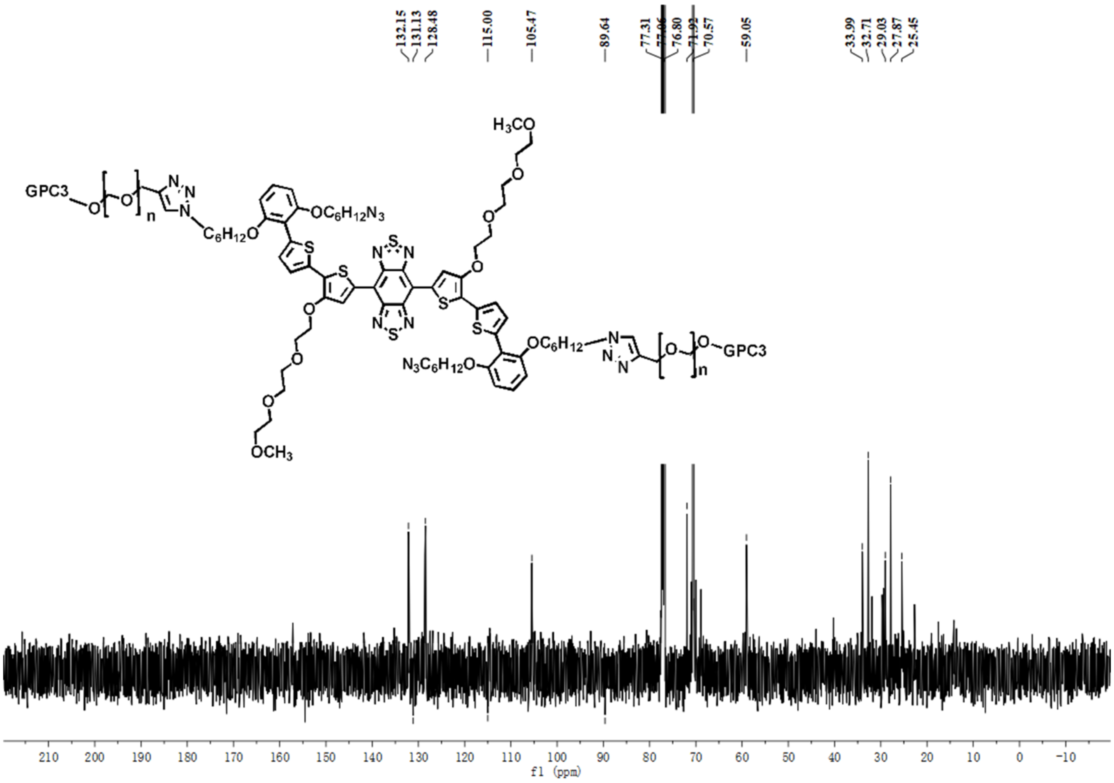

Figure S12. ^{13}C NMR of compound **BTOGP-GPC3**.

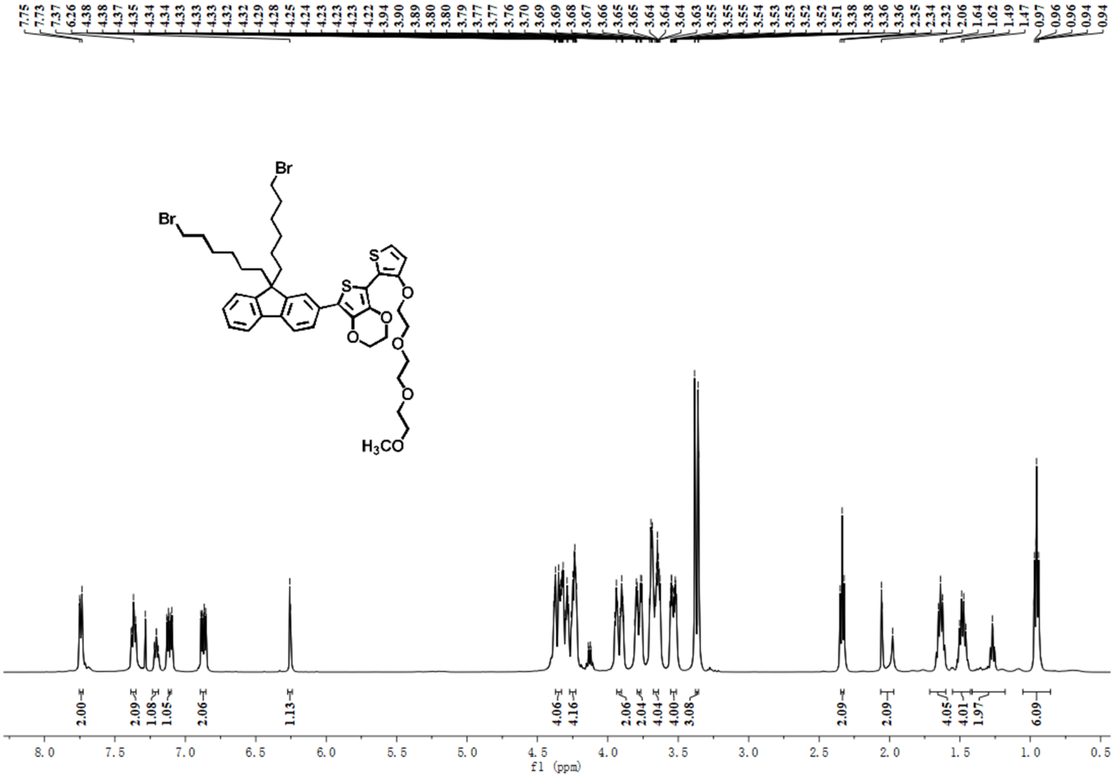

Figure S13. ^{1}H NMR of compound 12

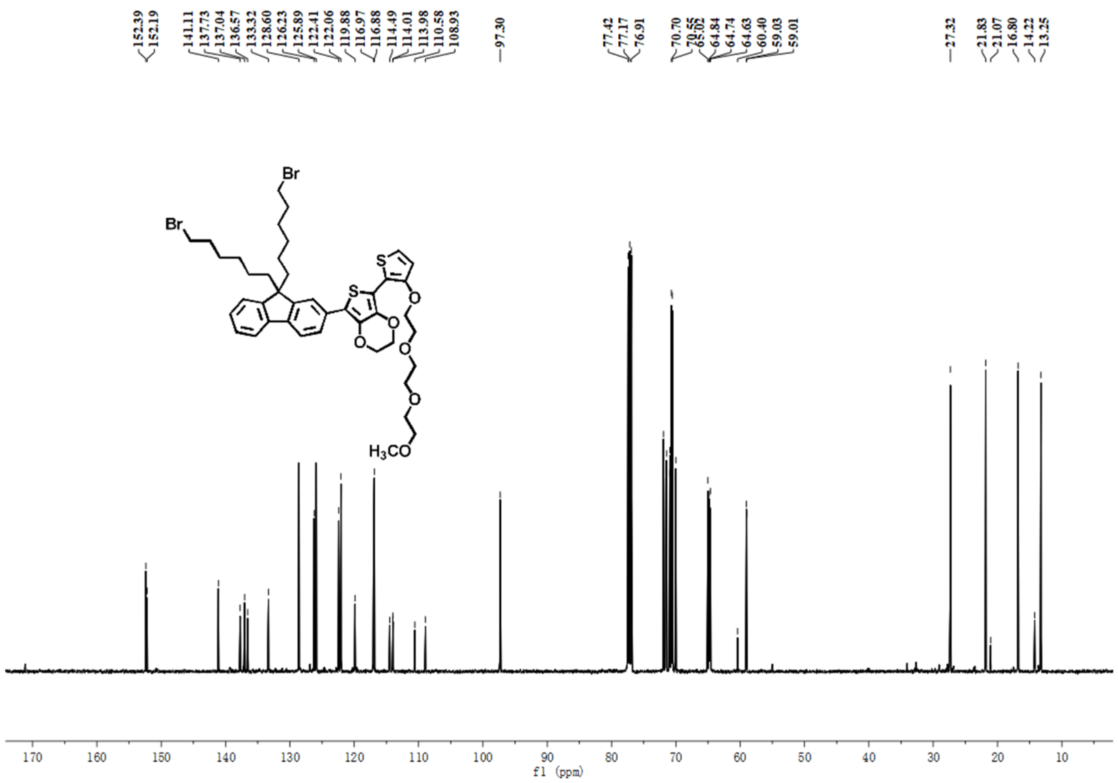

Figure S14. ^{13}C NMR of compound 12

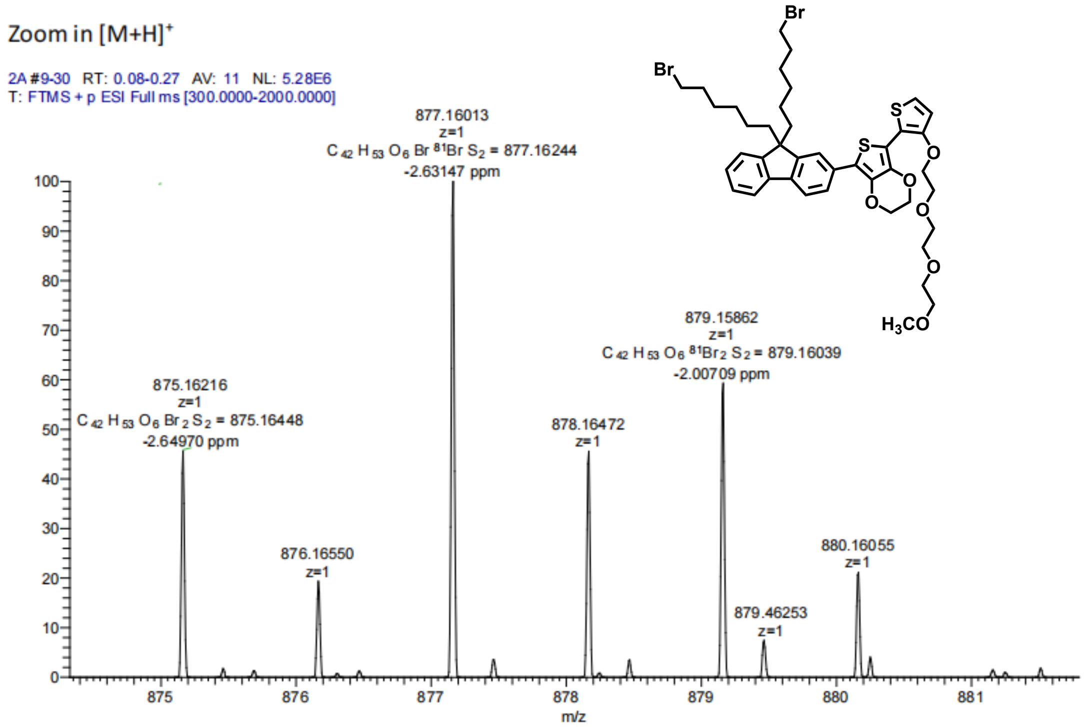

Figure S15 HRMS of compound 12

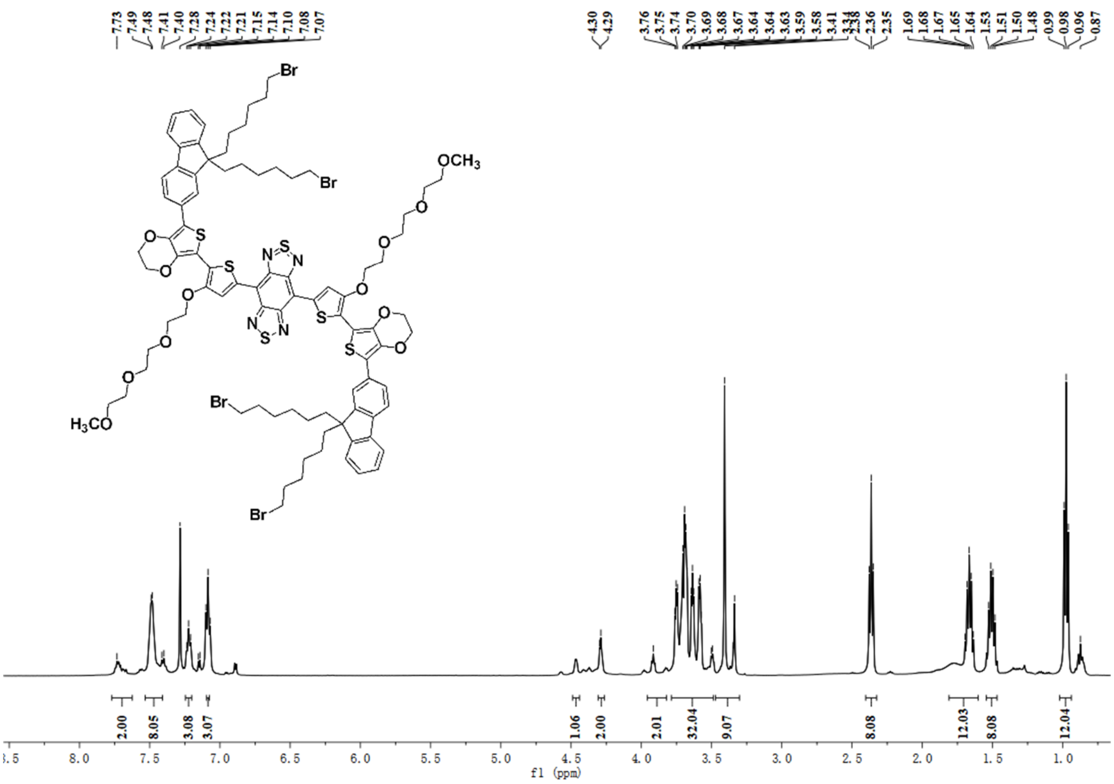

Figure S16. ^{1}H NMR of compound **IR-FEOG**

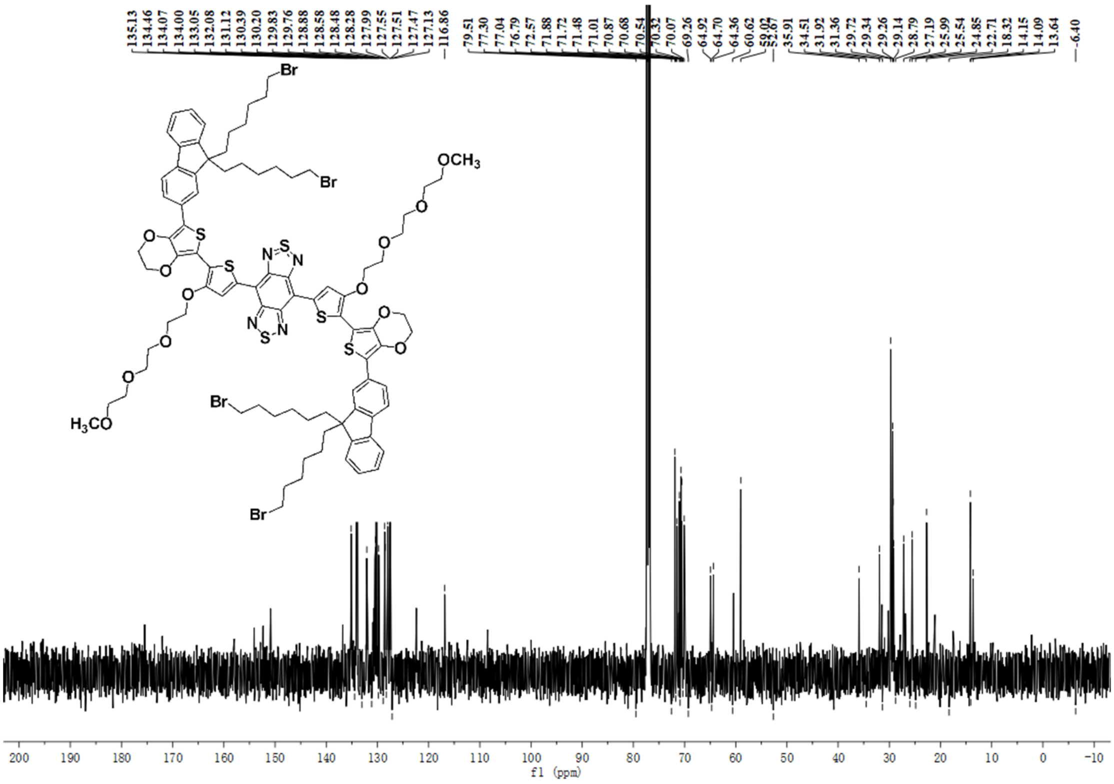

Figure S17. ^{13}C NMR of compound **IR-FEOG**.

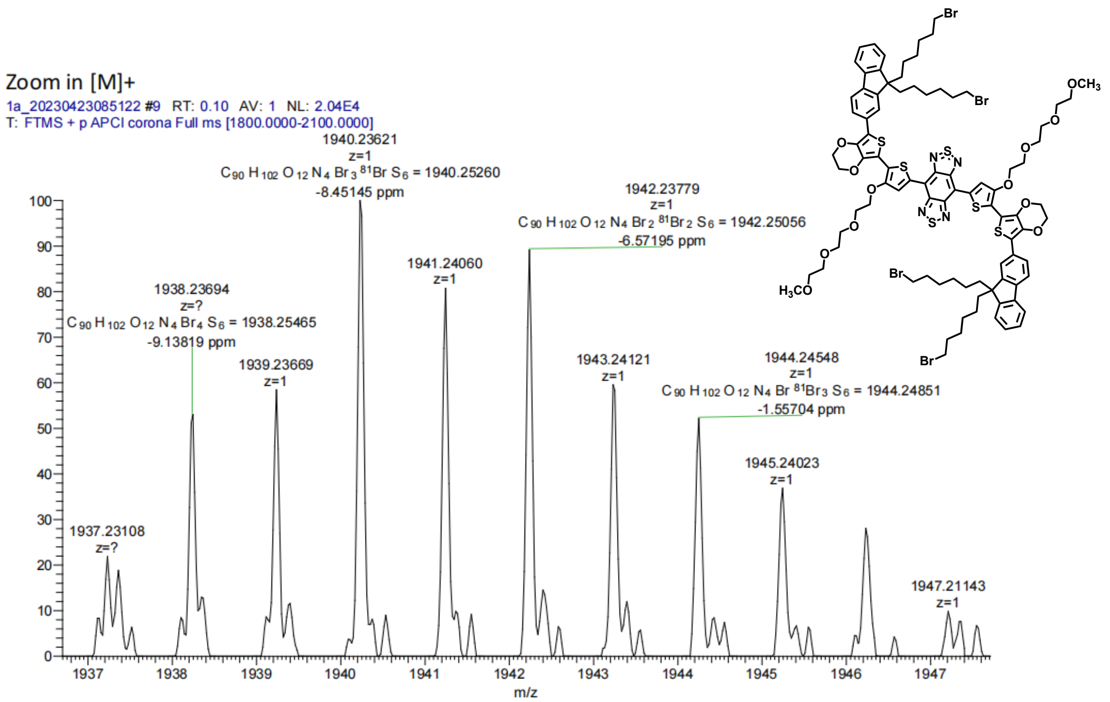

Figure S18. HRMS of compound **IR-FEOG**.

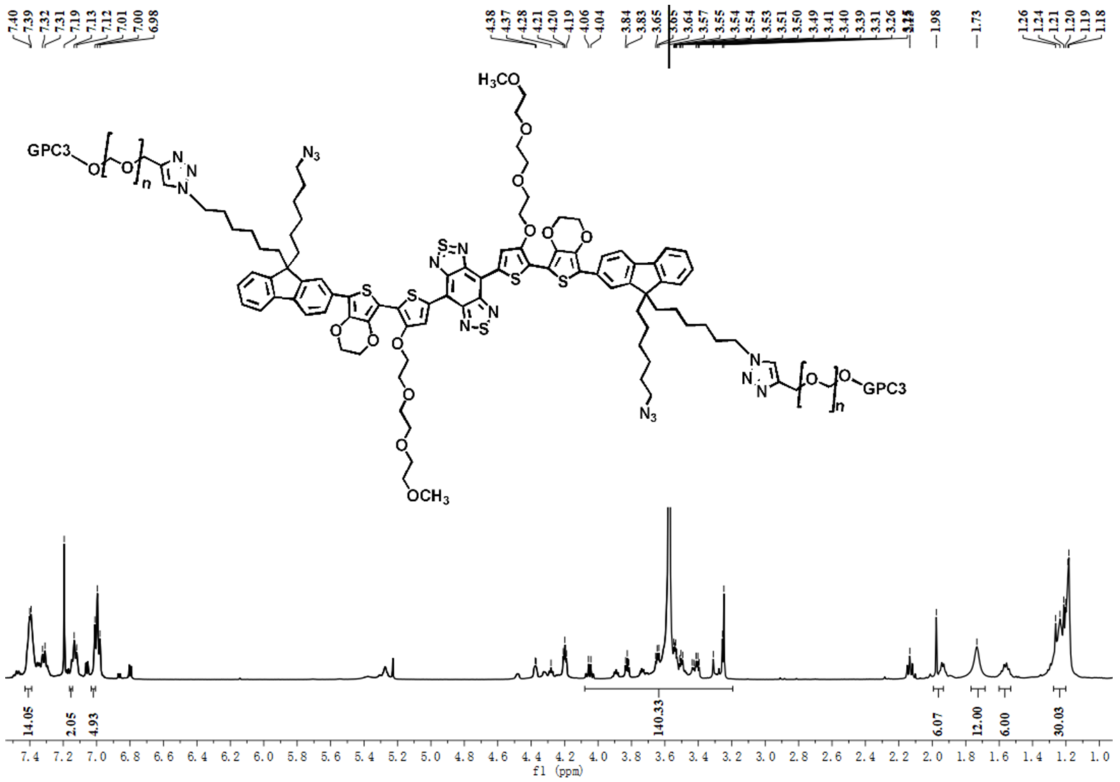

Figure S19. ^{1}H NMR of compound **FEOGP-GPC3**

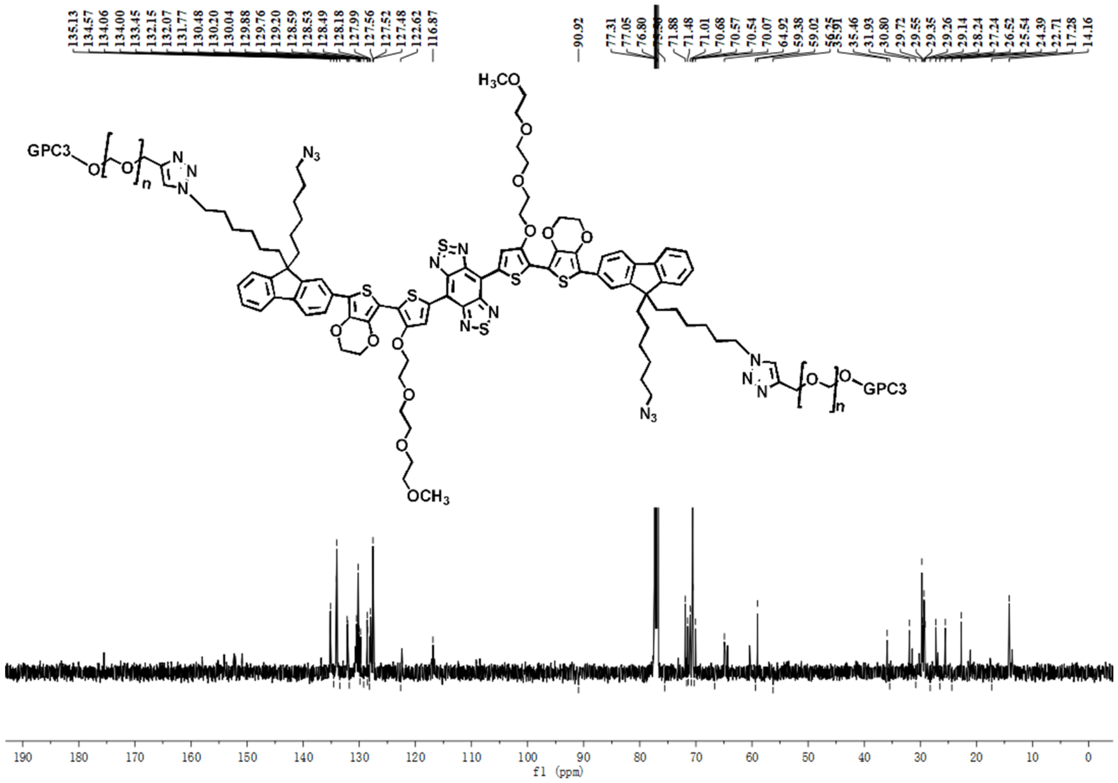

Figure S20. ^{13}C NMR of compound **FEOGP-GPC3**.

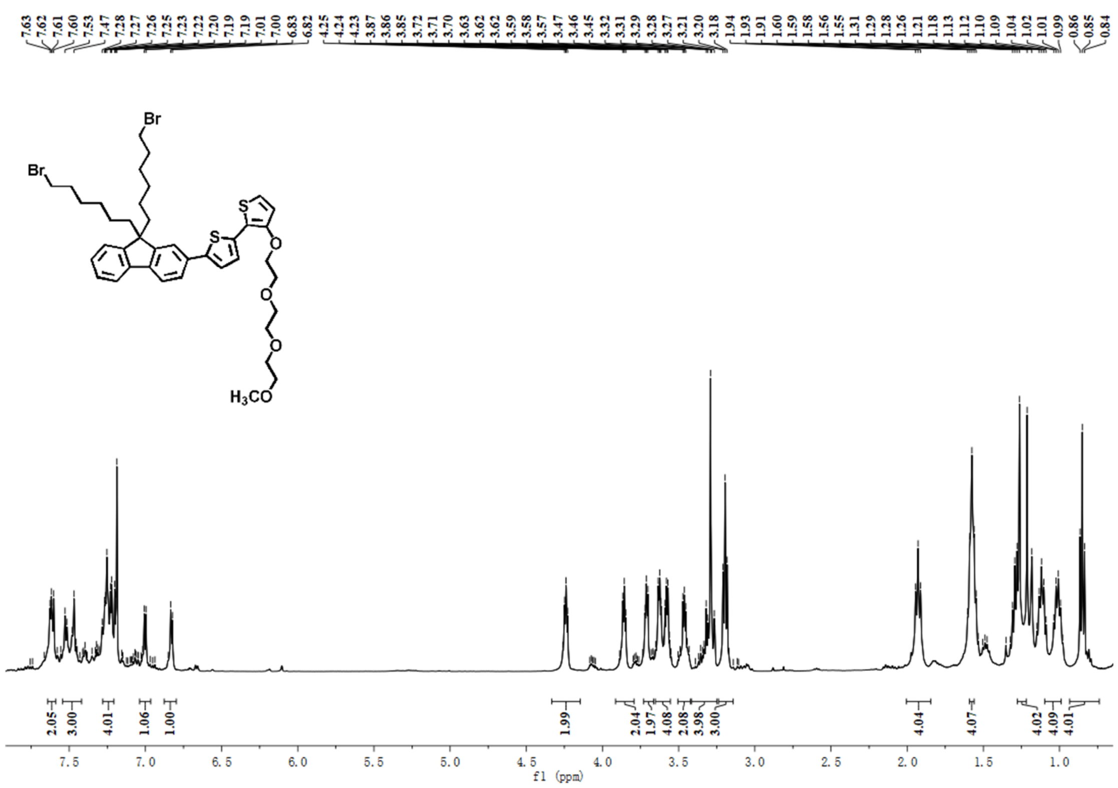

Figure S21. ^{1}H NMR of compound **15**

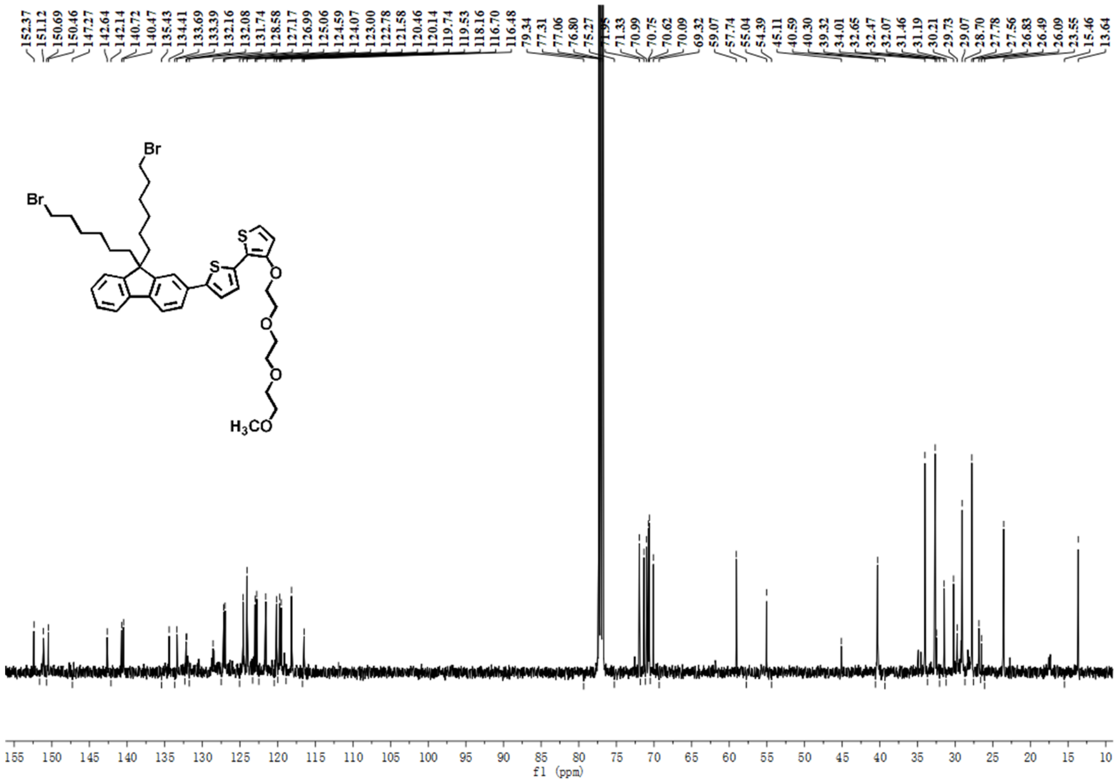

Figure S22. ^{13}C NMR of compound **15.**

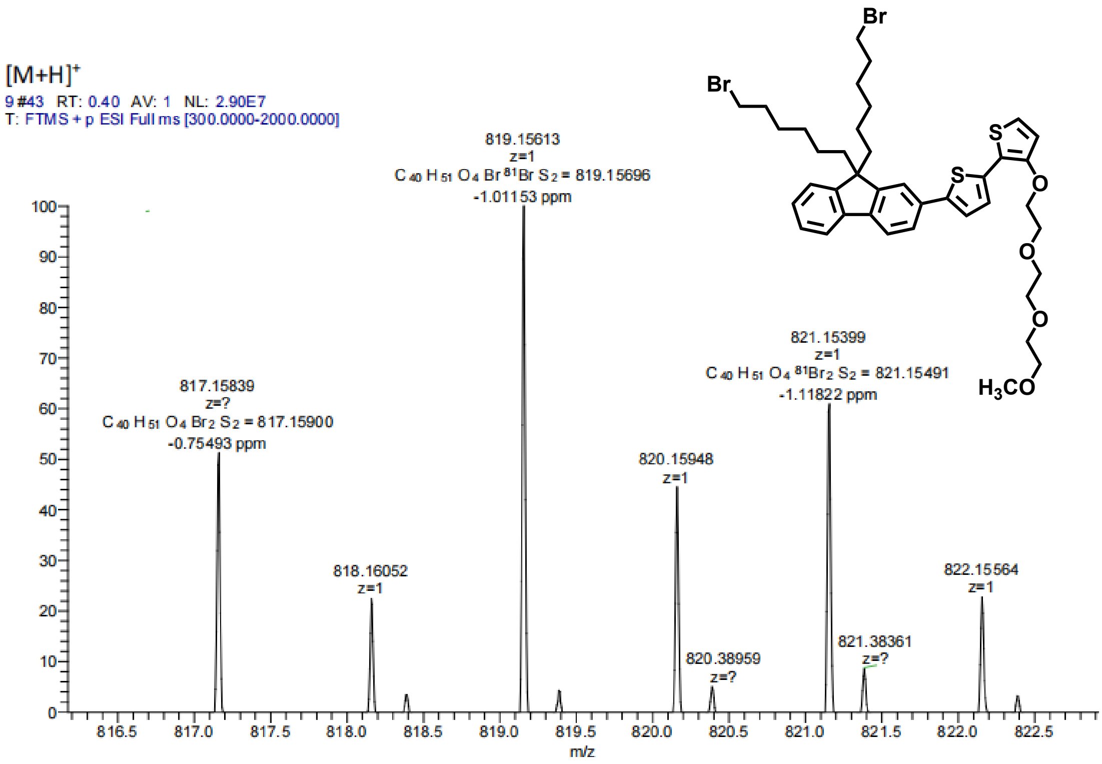

Figure S23. HRMS of compound **15.**

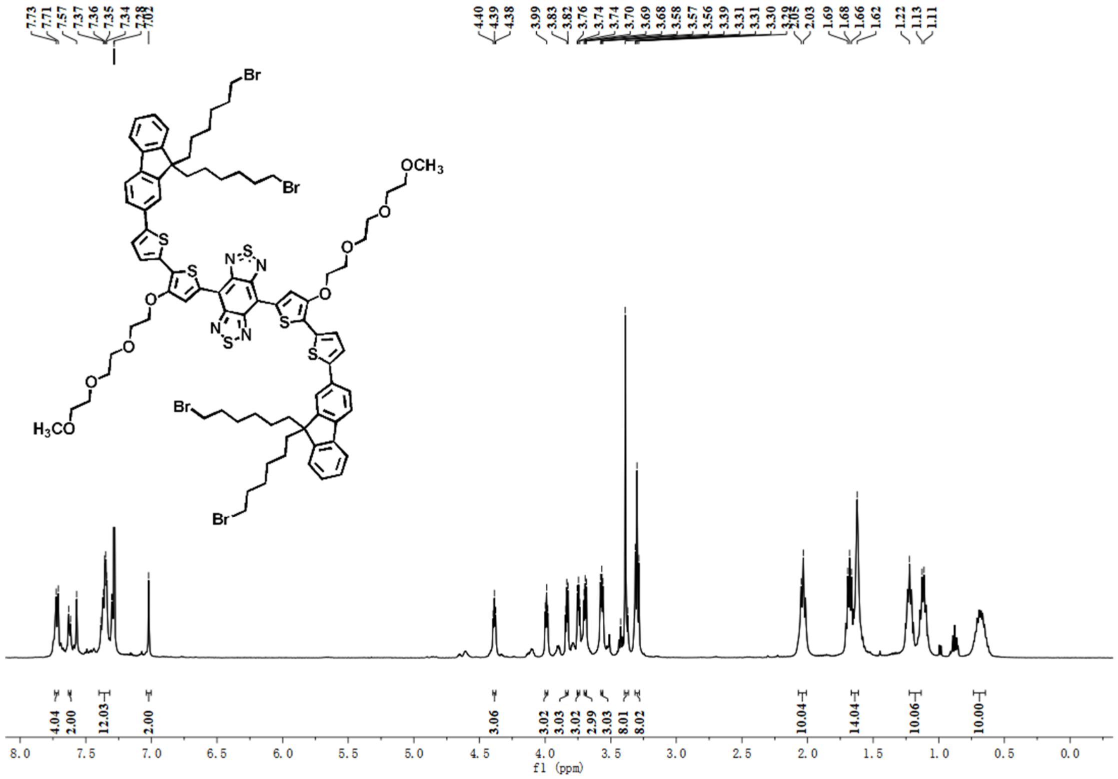

Figure S24. ^{1}H NMR of compound **IR-FTOG.**

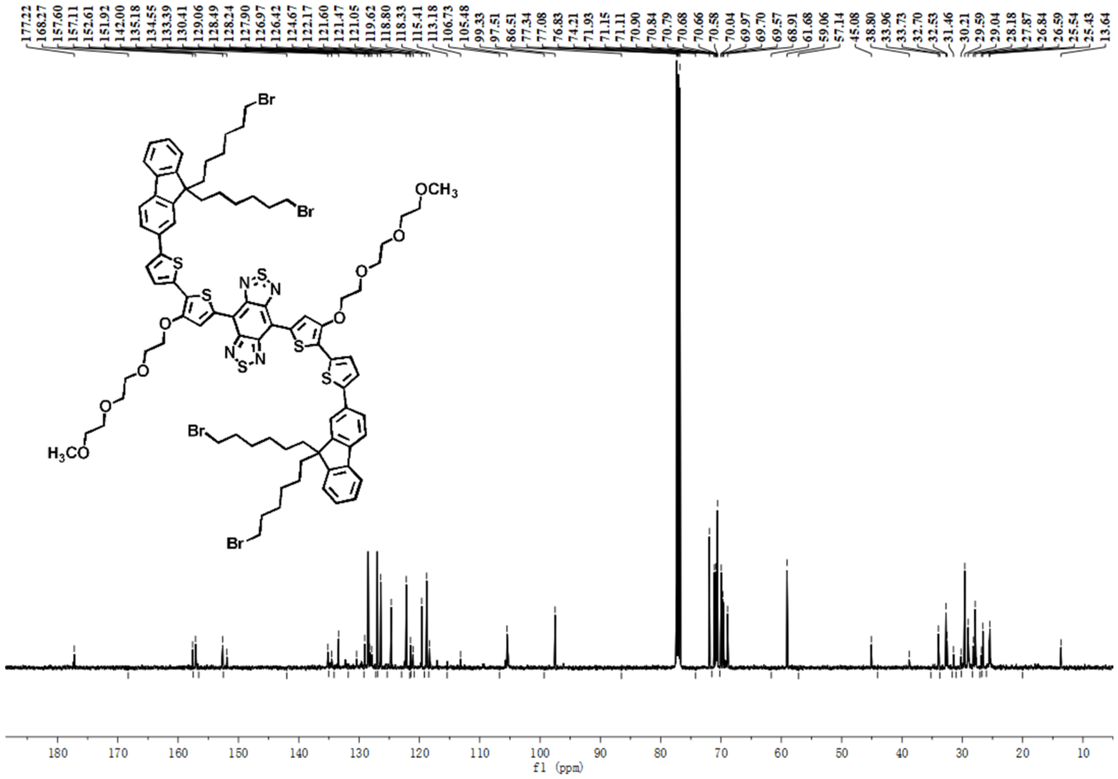

Figure S25. ^{13}C NMR of compound **IR-FTOG.**

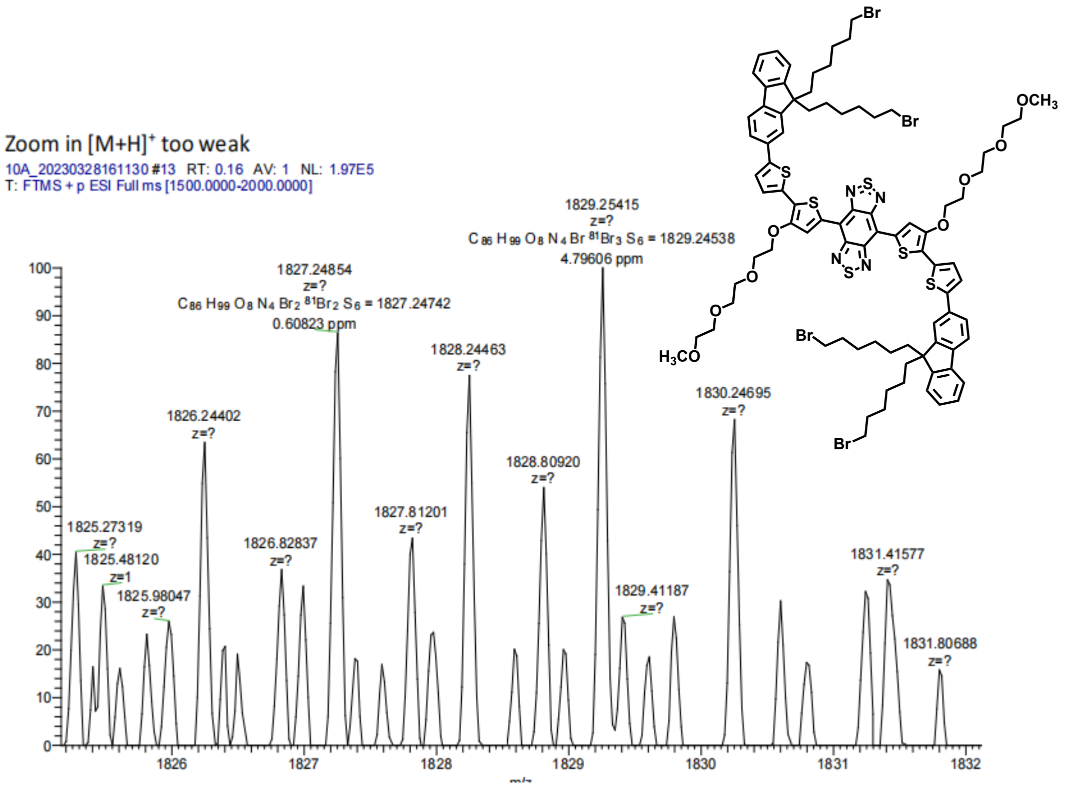

Figure S26. HRMS of compound **IR-FTOG.**

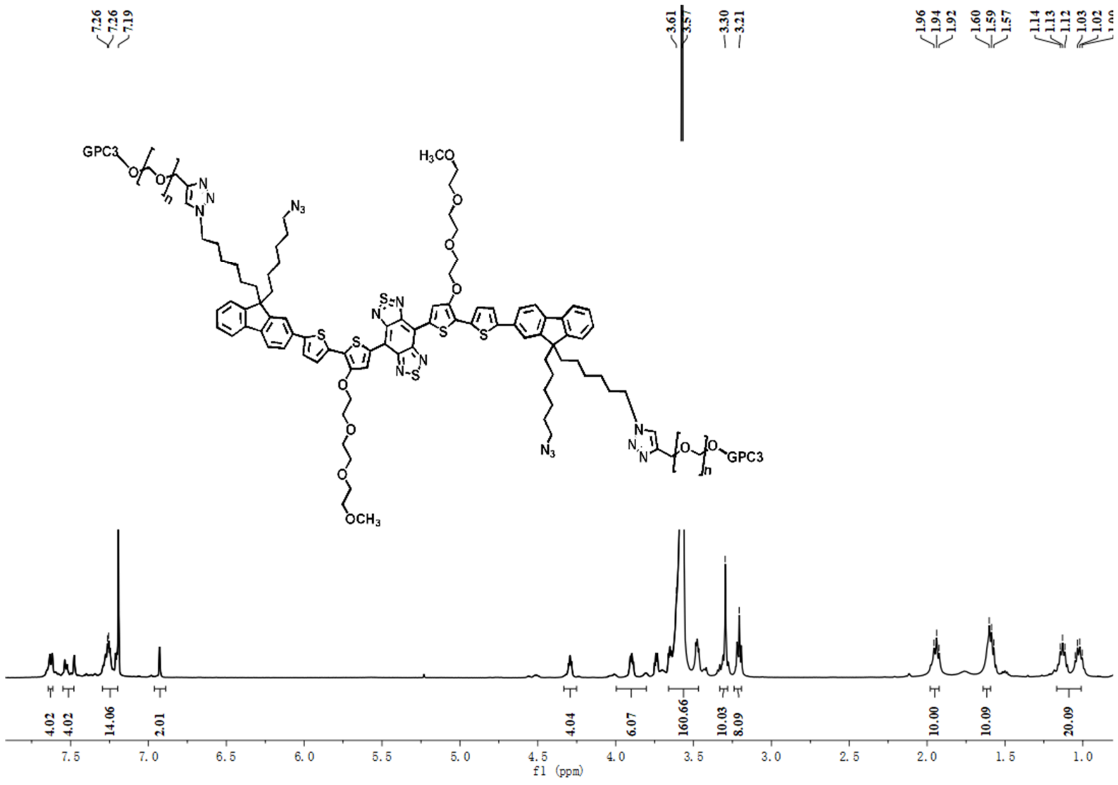

Figure S27. ^{1}HNMR of compound **FTOG-GPC3.**

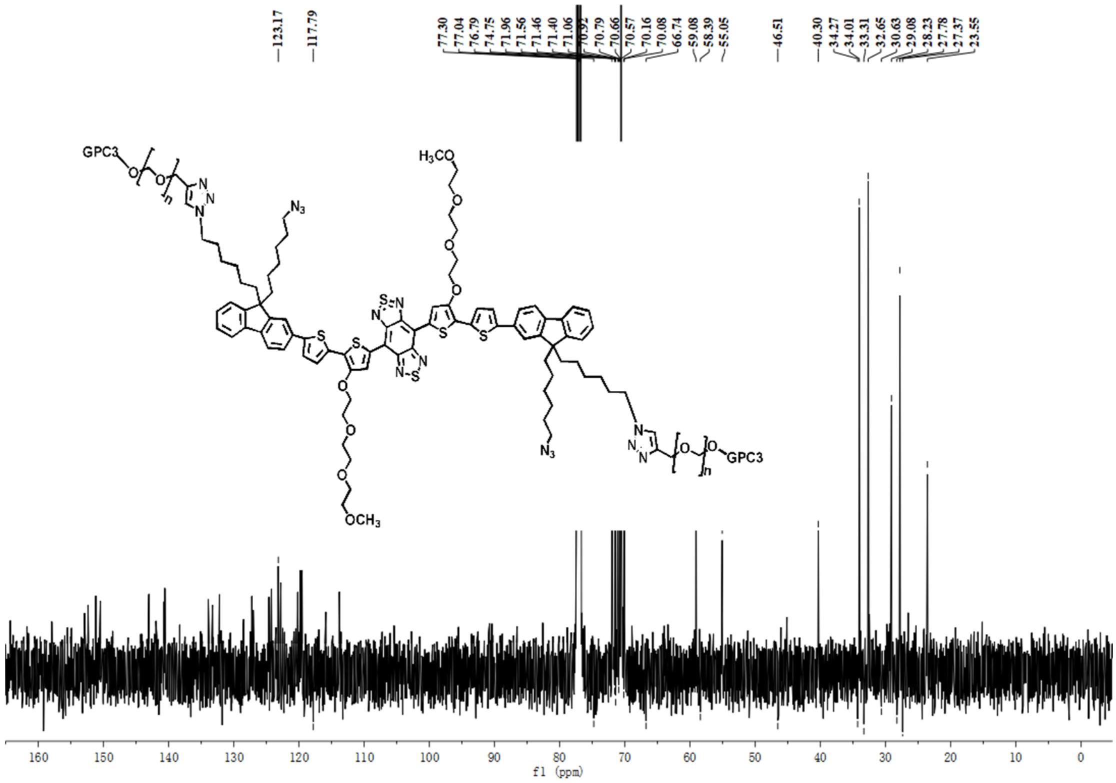

Figure S28. ^{13}C NMR of compound **FTOG-GPC3.**

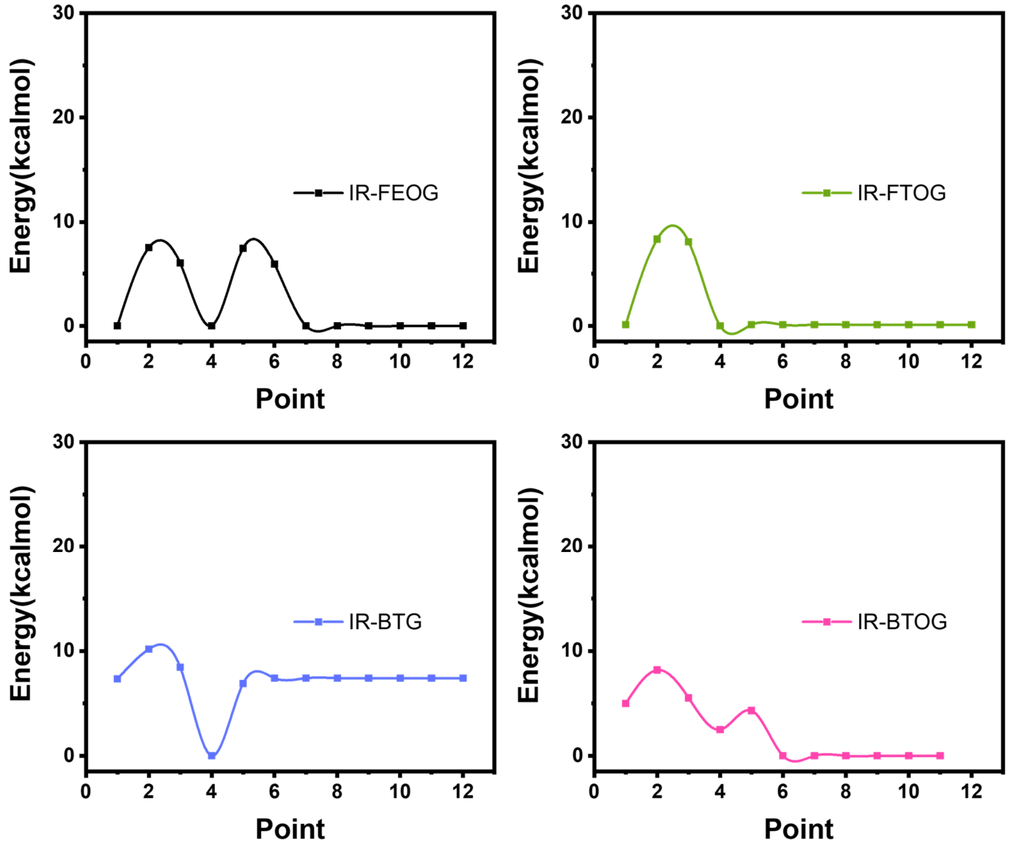

Figure S29. Lowest energy values obtained after geometry optimization and potential energy surface (PES) scans, with the dihedral angles optimized for each of the four molecules (IR-FEQS, IR-FTQG, IR-BTG, and IR-BTOG).

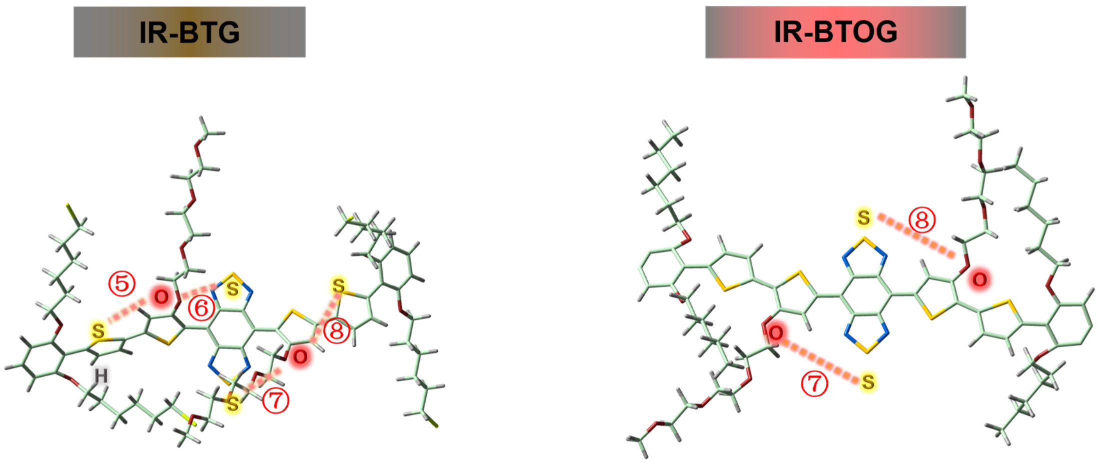

IR-BTG

	S•••O
⑤	5.61 Å, 97.26 °
⑥	4.34 Å, 101.98°
⑦	4.19 Å, 103.29°
⑧	6.36 Å, 124.08 °

IR-BTOG

	S•••O
⑦	5.44 Å, 62.97°
⑧	5.54 Å, 64.06 °

Figure S30. Computed weak S-O interactions in IR-BTG and IR-BTOG, along with their corresponding quantified values.

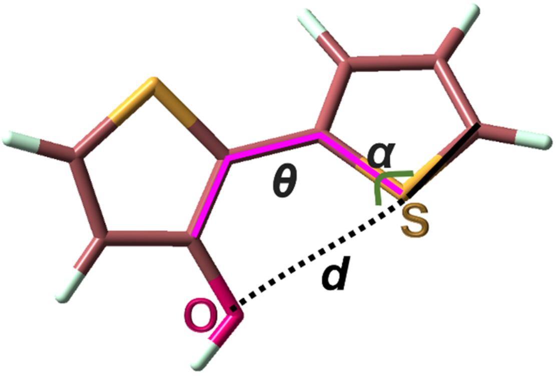

$$S = (-\cos\alpha) \bullet \cos^2\theta(1 - e^{\Delta d})^2$$

Figure S31. The geometrical parameters of the computational model of S-O conformation locks (SoCLs) (d, $\Delta d = drw - d$ ($d$rw: The values of the sum of van der Waals radii of two atoms)).

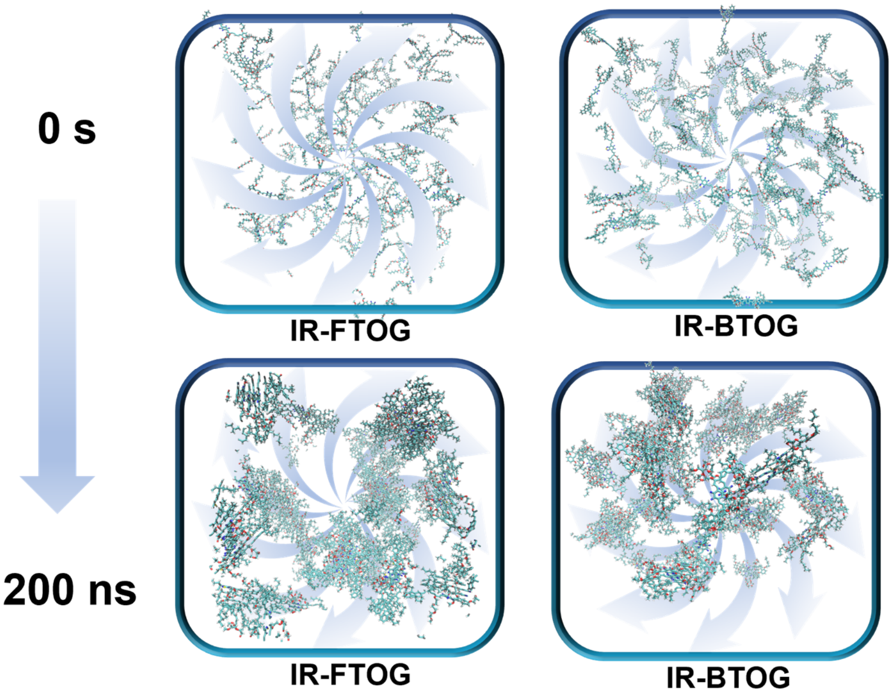

Figure S32. Molecular dynamics simulations of IR-FTOG and IR-BTOG**.**

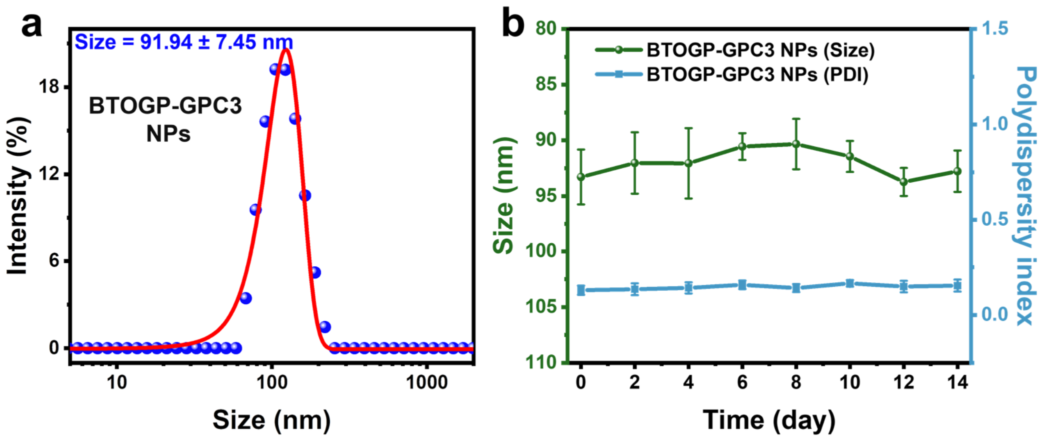

Figure S33. Nanoparticle size (a) and (b) 14-day stability of BTOGP-GPC3 nanoparticles.

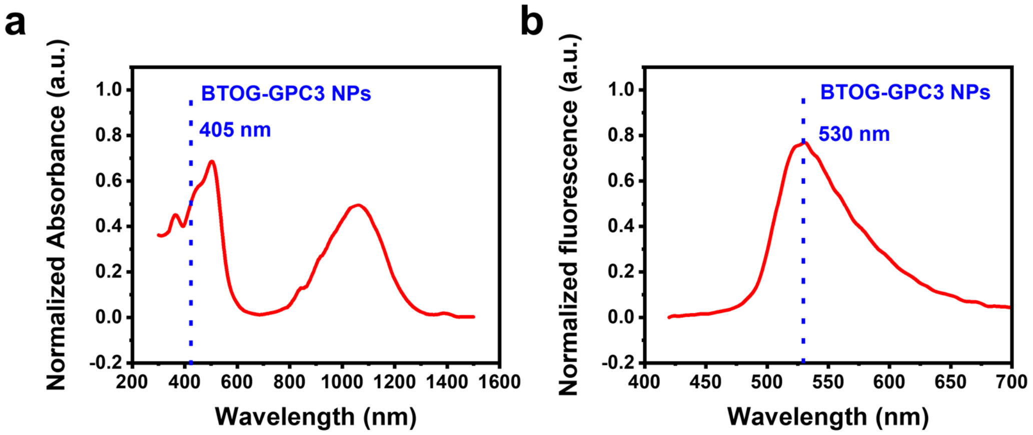

Figure S34. (a) Absorption spectra of BTOG-GPC3 NPs (300–1500 nm). (b) fluorescence emission spectra of BTOG-GPC3 NPs (λex = 405 nm).

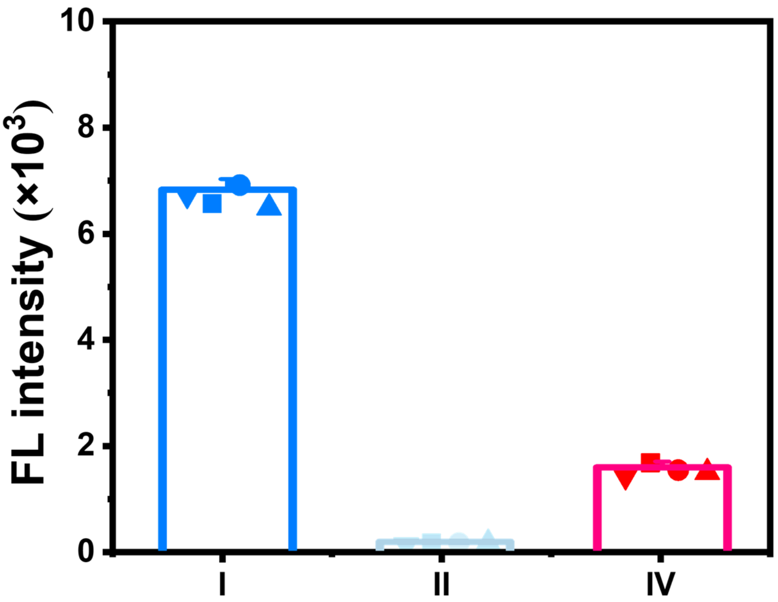

Figure S35. The Semi-quantification of the fluorescence intensity of Figure 5f.

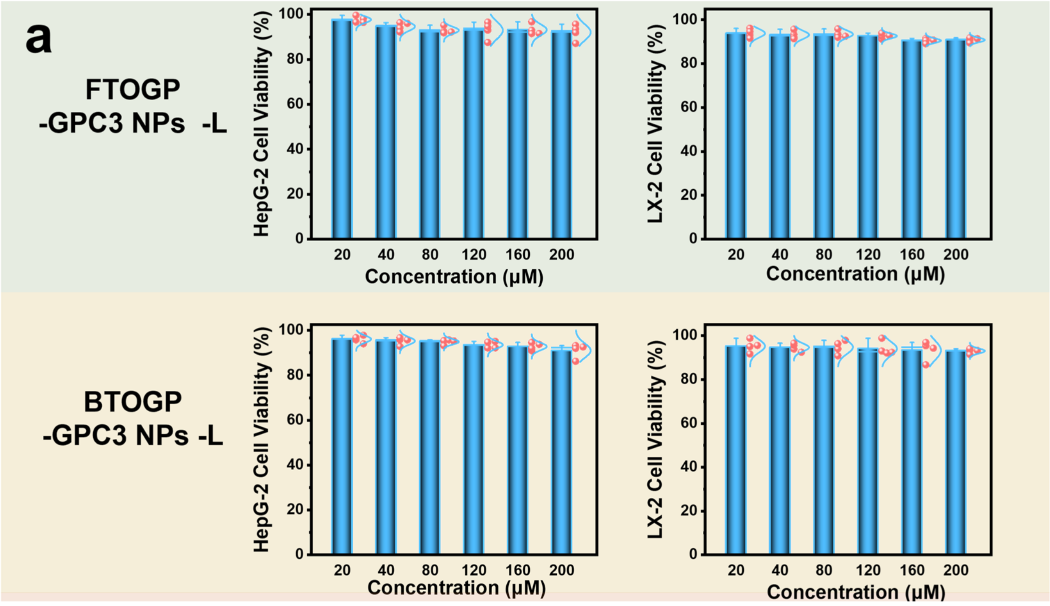

Figure S36. The CCK8 cell viability assays of FTOGP-GPC3 NPs -L and BTOGP-GPC3 NPs -L..

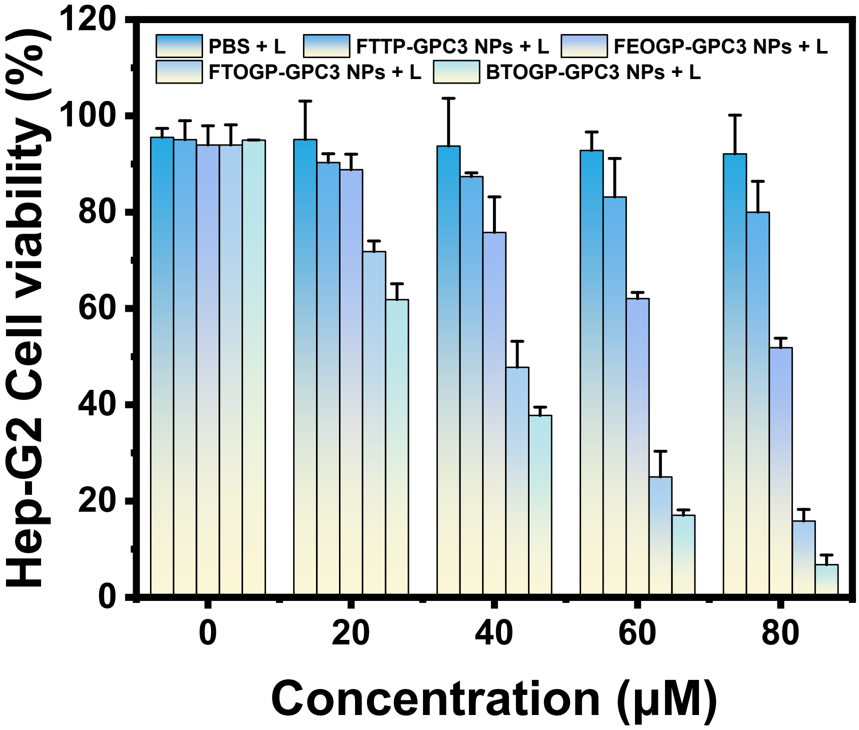

Figure S37. Cell viability of HepG-2 cells with different treatments**.**

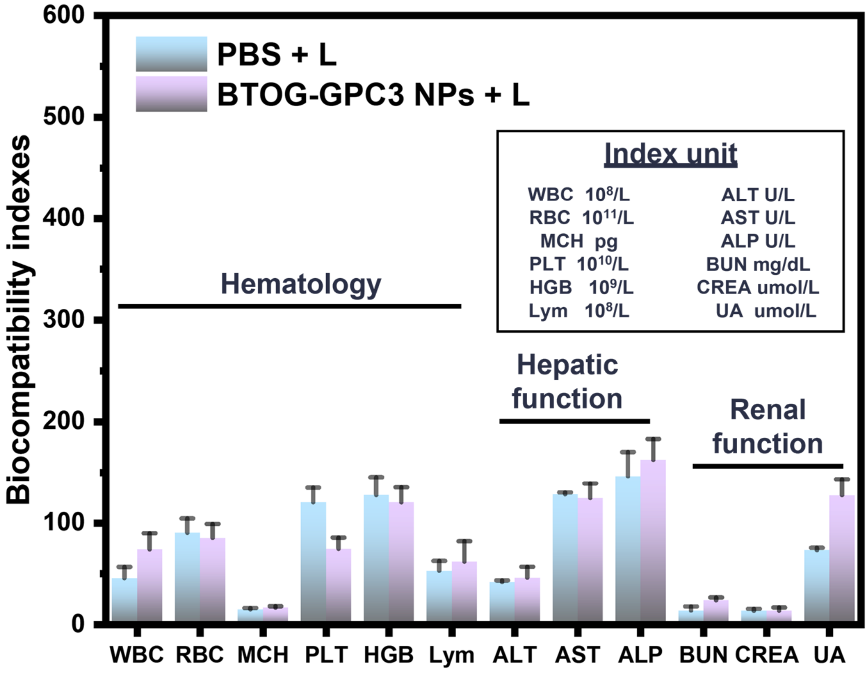

Figure S38. Whole blood and blood biochemistry of healthy BALB/c mice treated with PBS + L, BTOG-GPC3 NPs + L for 14 days. Error bars, mean ± SD (n = 3).

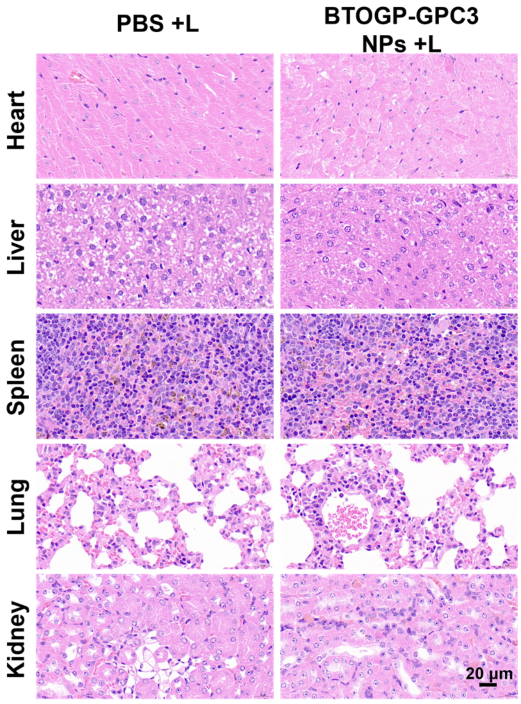

Figure S39. H&E staining images of significant organs from healthy BALB/c mice treated with PBS + L, BTOG-GPC3NPs + L.

3. Supplementary tables

Table S1. Calculated first vertical S_0-S_1 excitation energies (E_{01}), first vertical S_1-S_0 emission energies (E_{10}), electronic configurations, and reorganization energies determined at the B3LYP */6-31G g (d,p) level.

		Absorption ($S_0 \rightarrow S_1$)			Emission ($S_1 \rightarrow S_0$)		
Compound	ω*	E_{01} eV (nm)	f_{01}	Elec. Config.	E_{10} eV (nm)	f_{10}	Reorganization energy (nm) Exp
IR-FEOG	0.116	1.247 (993)	0.67	H→L 100%	1.03 (1208)	0.13	0.22 (214)
IR-FTOG	0.112	1.168 (1061)	0.95	H→L 100%	0.91 (1349)	0.02	0.25 (287)
IR-BTG	0.116	1.457 (850)	0.54	H→L 100%	1.09 (1135)	0.46	0.37 (284)
IR-BTOG	0.113	1.130 (1096)	0.77	H→L 100%	0.90 (1376)	0.72	0.23 (279)

The reorganization energies are obtained as E_{01} minus E_{10}.

Table S2. The optimized dihedral angles (θ), orbital plane angles (α), bond lengths (*d*, Δ*d* = *d*rw–*d* (*d*rw: The values of the sum of van der Waals radii of two atoms)), and descriptors $S = (-\cos\alpha) \bullet \cos^2\theta(1 - e^{\Delta d})^2$ for IR-FEOG, IR-FTOG, IR-BTG and IR-BTOG.

Systems	Location	θ (°)	α (°)	*d* (Å)	*Δd* (Å)	*S*
IR-FEOG（S···O）	①	32.57	107.59	4.44	-1.19	0.104
	②	32.57	92.0	4.73	-1.48	0.015
IR-FEOG（S···N）	①	4.24	95.78	2.78	0.57	0.059
	②	4.24	94.89	2.80	0.55	0.046
IR-FEOG（H···N）	①	4.24	103.82	2.22	0.64	0.191
	②	4.24	103.19	2.17	0.69	0.224
IR-FTOG（S···O）	①	15.55	146.40	2.93	0.32	0.110
	②	15.55	113.77	2.89	0.36	0.070
IR-FTOG（S···N）	①	1.37	165.00	2.76	0.59	0.624
	②	1.37	164.84	2.79	0.56	0.543
IR-FTOG（H···N）	①	1.37	119.80	2.17	0.69	0.490
	②	1.37	119.23	2.18	0.68	0.463
IR-BTG（S···O）	①	10.15	141.18	3.01	0.24	0.056
	②	10.15	91.67	3.20	0.05	0.001
	③	10.15	153.15	2.49	0.76	1.120
	④	10.15	94.82	2.82	0.43	0.024
	⑤	28.63	97.26	5.61	-2.36	0.080
	⑥	47.55	101.98	4.34	-1.09	0.042
	⑦	28.63	103.29	4.19	-0.94	0.066
	⑧	47.55	124.08	6.36	-3.12	0.233
IR-BTG（S···N）	①	47.55	139.67	2.91	0.44	0.106
	②	47.55	152.76	3.12	0.23	0.027
IR-BTG（N···H）	①	47.55	115.95	2.79	0.07	0.001
	②	47.55	99.49	2.77	0.09	0.001
IR-BTG（O···H）	①	10.15	108.20	2.19	0.51	0.134
	②	10.15	107.50	2.25	0.45	0.094

IR-BTOG（S···O）	①	10.28	170.63	2.73	0.52	0.444
	②	10.28	94.53	3.13	0.12	0.001
	③	3.55	144.90	3.06	0.19	0.036
	④	3.55	140.02	3.01	0.24	0.056
	⑤	10.28	137.16	2.54	0.71	0.759
	⑥	10.28	97.95	3.01	0.24	0.010
	⑦	0.57	62.97	5.44	-2.19	-
	⑧	0.57	64.06	5.54	-2.29	-
IR-BTOG（S···N）	①	0.57	154.29	2.79	0.56	0.507
	②	0.57	164.67	2.77	0.58	0.595
IR-BTOG（N···H）	①	0.57	120.39	2.16	0.7	0.519
	②	0.57	120.54	2.17	0.69	0.501
IR-BTOG（O···H）	①	10.28	123.18	1.83	0.87	1.019
	②	10.28	131.30	1.86	0.84	1.107

Table S3. Cartesian coordinates for the DFT optimized structure of IR-BTOG.

Atom	X	Y	Z
C	-2.593	0.249	-0.525
C	-3.397	-0.385	-1.477
C	-4.762	-0.175	-1.274
C	-5.053	0.613	-0.158
S	-3.596	1.125	0.624
O	-5.766	-0.662	-2.034
C	-5.505	-1.797	-2.843
C	-6.871	-2.287	-3.286
O	-6.698	-3.494	-3.985
C	-7.91	-4.076	-4.411
C	-8.588	-4.856	-3.289
O	-9.841	-5.281	-3.763
C	-10.594	-5.951	-2.785
C	-11.945	-6.293	-3.385
O	-12.697	-6.92	-2.379
C	-13.99	-7.289	-2.794
C	-6.359	0.929	0.35
C	-7.577	0.788	-0.29
C	-8.671	1.161	0.516
C	-8.316	1.6	1.769
S	-6.589	1.552	1.962
C	-9.214	2.011	2.86
C	-10.364	1.245	3.161
C	-11.211	1.61	4.207
C	-10.915	2.732	4.976
C	-9.793	3.499	4.706
C	-8.956	3.141	3.652
O	-7.834	3.895	3.437
C	-7.858	4.737	2.282
C	-8.703	5.985	2.504
C	-8.814	6.86	1.251
C	-7.478	7.417	0.752
C	-7.66	8.394	-0.412
C	-6.325	8.92	-0.911
Br	-6.555	10.176	-2.393
O	-10.567	0.151	2.389
C	-11.581	-0.773	2.738

C	-11.405	-1.994	1.852
C	-12.487	-3.046	2.093
C	-12.229	-4.346	1.331
C	-13.363	-5.361	1.508
C	-13.015	-6.694	0.868
Br	-14.513	-7.954	0.995
C	-1.173	0.236	-0.458
C	-0.442	0.937	0.553
C	1.013	0.931	0.648
C	1.831	0.21	-0.283
C	1.099	-0.489	-1.296
C	-0.355	-0.483	-1.391
N	-0.805	-1.205	-2.414
S	0.496	-1.837	-3.19
N	1.682	-1.21	-2.246
N	1.463	1.653	1.671
S	0.161	2.286	2.446
N	-1.024	1.66	1.502
C	3.25	0.184	-0.209
C	4.055	0.835	0.73
C	5.42	0.616	0.538
C	5.709	-0.21	-0.554
S	4.25	-0.706	-1.345
O	6.42	1.116	1.296
C	6.054	1.899	2.42
C	7.355	2.292	3.11
O	7.131	3.134	4.212
C	6.706	2.471	5.381
C	6.697	3.496	6.5
O	6.256	2.844	7.663
C	6.197	3.695	8.78
C	5.702	2.875	9.957
O	5.648	3.725	11.073
C	5.195	3.068	12.226
C	6.99	-0.587	-1.067
C	6.56	-1.22	-2.219
C	7.619	-1.705	-3.013
C	8.869	-1.466	-2.486
S	8.73	-0.614	-0.975
C	9.747	-2.543	-3.12
C	10.913	-3.169	-2.619

C	11.685	-4	-3.431
C	11.309	-4.224	-4.751
C	10.179	-3.616	-5.274
C	9.413	-2.781	-4.465
O	8.282	-2.23	-5.003
C	8.355	-0.837	-5.315
C	9.118	-0.582	-6.608
C	9.277	0.909	-6.923
C	7.956	1.652	-7.137
C	8.175	3.099	-7.586
C	6.856	3.829	-7.772
Br	7.132	5.676	-8.354
O	11.213	-2.922	-1.322
C	12.278	-3.622	-0.708
C	12.304	-3.214	0.755
C	13.413	-3.922	1.532
C	13.449	-3.517	3.005
C	14.561	-4.227	3.781
C	14.575	-3.809	5.241
Br	15.997	-4.712	6.234
H	-4.993	-2.574	-2.263
H	-4.882	-1.531	-3.705
H	-7.353	-1.53	-3.925
H	-7.495	-2.412	-2.389
H	-7.666	-4.75	-5.236
H	-8.605	-3.312	-4.79
H	-8.714	-4.22	-2.398
H	-7.961	-5.712	-2.995
H	-10.741	-5.313	-1.897
H	-10.089	-6.871	-2.453
H	-11.813	-6.957	-4.254
H	-12.438	-5.373	-3.737
H	-14.563	-6.416	-3.141
H	-14.487	-7.723	-1.924
H	-13.954	-8.03	-3.606
H	-7.663	0.436	-1.308
H	-9.703	1.102	0.195
H	-12.096	1.027	4.43
H	-11.577	3.008	5.791
H	-9.543	4.377	5.291
H	-8.222	4.176	1.411

H	-6.809	4.987	2.104
H	-8.275	6.562	3.333
H	-9.708	5.676	2.814
H	-9.489	7.697	1.468
H	-9.289	6.284	0.445
H	-6.823	6.595	0.432
H	-6.962	7.921	1.581
H	-8.288	9.237	-0.098
H	-8.185	7.898	-1.237
H	-5.688	8.122	-1.295
H	-5.787	9.469	-0.138
H	-12.573	-0.321	2.591
H	-11.488	-1.053	3.797
H	-10.412	-2.419	2.044
H	-11.416	-1.675	0.803
H	-13.466	-2.64	1.804
H	-12.552	-3.271	3.167
H	-11.282	-4.783	1.677
H	-12.099	-4.129	0.262
H	-14.283	-4.97	1.058
H	-13.57	-5.51	2.575
H	-12.181	-7.184	1.373
H	-12.798	-6.606	-0.197
H	5.406	1.316	3.087
H	5.511	2.799	2.106
H	7.977	2.853	2.406
H	7.904	1.384	3.404
H	7.392	1.649	5.636
H	5.698	2.047	5.273
H	6.028	4.329	6.233
H	7.709	3.909	6.634
H	5.511	4.538	8.6
H	7.189	4.114	9.012
H	6.386	2.029	10.132
H	4.709	2.458	9.725
H	4.177	2.67	12.092
H	5.186	3.802	13.035
H	5.858	2.234	12.505
H	5.514	-1.331	-2.467
H	7.477	-2.214	-3.956
H	12.574	-4.48	-3.041

H	11.913	-4.876	-5.375	1
H	9.865	-3.772	-6.3	
H	8.815	-0.286	-4.483	2
H	7.313	-0.517	-5.392	
H	8.602	-1.09	-7.432	
H	10.112	-1.037	-6.524	
H	9.894	1.012	-7.825	
H	9.837	1.395	-6.112	
H	7.367	1.649	-6.211	
H	7.357	1.118	-7.889	
H	8.736	3.117	-8.528	
H	8.782	3.632	-6.845	
H	6.289	3.893	-6.843	
H	7.367	1.649	-6.211	
H	7.357	1.118	-7.889	
H	8.736	3.117	-8.528	
H	8.782	3.632	-6.845	
H	6.289	3.893	-6.843	
H	6.236	3.365	-8.541	
H	13.231	-3.37	-1.196	
H	12.122	-4.706	-0.806	
H	11.327	-3.44	1.197	
H	12.437	-2.127	0.815	
H	14.387	-3.7	1.072	
H	13.278	-5.01	1.46	
H	12.477	-3.74	3.466	
H	13.585	-2.429	3.079	
H	15.535	-4.002	3.33	
H	14.427	-5.314	3.717	
H	13.646	-4.069	5.75	
H	14.764	-2.741	5.358	
H	-2.974	-0.974	-2.277	
H	3.626	1.441	1.513	

Table S4. LUMO composition analysis results of the molecular fluorophores IR-FEOG, IR-FTOG, IR-BTG, and IR-BTOG based on the Hirshfeld method by Multiwfn.

Dye	Acceptor	Donor 1	Donor 2	Shielding unit
IR-FEOG	79.18%	12.01%	7.01%	1.8%
IR-FTOG	86.48%	11.67%	2.68%	0.83%
IR-BTG	91.24%	7.96%	0.51%	0.29%
IR-BTOG	68.48%	22.15%	6.65%	2.72%

Table S5. Optical data of NIR-II fluorophores

Dye	ε(10^{3}L/mol·c) (1064 nm)	λ_{abs} (nm)	λ_{em} (nm)	Stokes shift (nm)	QY (%) (λex: 1064 nm)
IR-FTT [a]	23	890	1112	222	0.0596
IR-FEOG [a]	30.8	944	1140	196	0.1353
IR-FTOG [a]	34.3	1032	1242	208	0.1731
IR-BTOG [a]	52.5	1051	1303	252	0.0956
FTTP-GPC3 NPs [b]	22	1010	-	-	0.0003
FEOGP-GPC3 NPs [b]	33.4	952	1151	199	0.0106
FTOGP-GPC3 NPs [b]	39.9	1034	1250	216	0.0126
BTOGP-GPC3 NPs [b]	45.4	1062	1316	205	0.00736

[a]) measured in DCM; [b]) measured in water.

Table S6. Fitting parameters of ground states and excited states of materials.

Sample	Wavelength (nm)	τ_1 (ps)	τ_2 (ps)	τ_3 (ps)	$\tau_{\text{average,decay}}$ (ps)	$\tau_{\text{average,decay}}^{-1}$ (ps^{-1})
IR-FTOG NPs	1141	0.82 (74.0%)	12.24 (17.2%)	580.77 (8.8%)	37.16	0.027
IR-BTOG NPs	1141	0.173 (91.09 %)	2.23 (4.85%)	20.10 (4.06%)	1.069	0.935

Table S7. Summary of photothermal agents (PTAs) for phototheranostics application. (η = photothermal conversion efficiency， 1064 nm).

PTAs	Laser irradiation condition	Chemical structure	Power (W cm^{-2})	Φ%	η	Reference
Organic PTAs （small molecules and semiconducting polymers NPs）						
TPABT-TD NPs	1064 nm	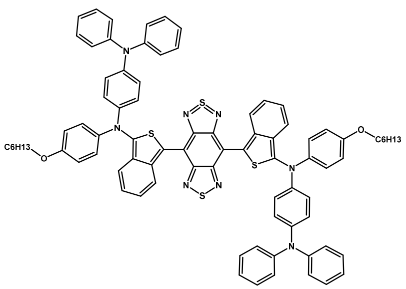	1	NA	68.98 %	***Angew. Chem. Int. Ed*. 2024**, 63, e202401877
Q-T NPs	1064 nm	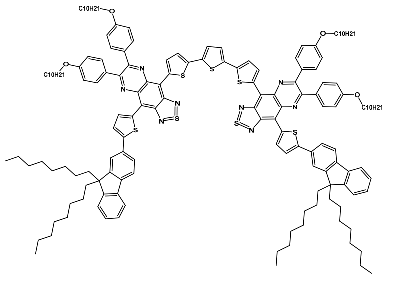	1	0.16%	73%	***Small.* 2023**, 19, 2205640

BAF4 NPs	1064 nm	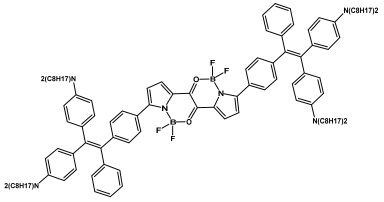	0.75	NA	80	***Angew. Chem. Int. Ed.* 2021**, 60, 22376–22384
BETA NPs	1064 nm	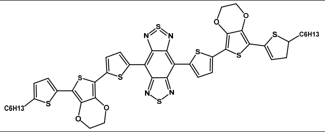	1	0.019%	47.6	***Angew. Chem. Int. Ed.* 2023**, 62, e202215372
SW-8 Nps	1064 nm	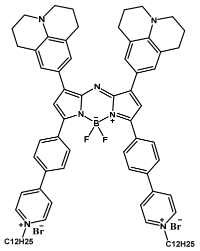	0.23	NA	75	***Research*. 2023**, 6, 0169
BETT-2	1064 nm	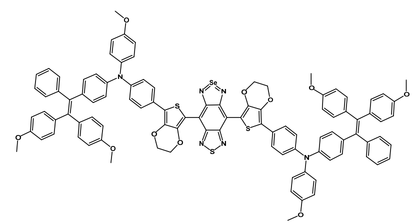	0.8	0.13	56.6	***Adv. Mater.* 2024**, 36, 2309748
IR-SS NPs	1064 nm	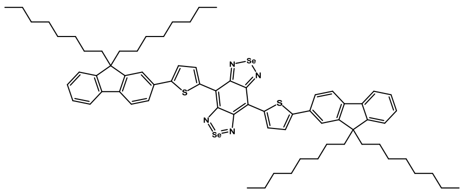	1	NA	77	***Adv. Mater.* 2020**, 32, 2001146

Inorganic PTAs, including lanthanide-doped NPs, QDs, etc						
AuNR@NF1064	1064 nm	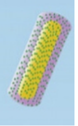	1	NA	68.5 %	***Small*. 2024**, 10.1002/smll.202407787
Ni-CDs	1064	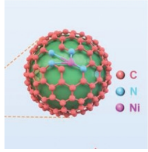	0.8	NA	76.1	***Adv Funct Mater***. 2021, 31(26): 2100549.
PtNP-shell-PEG	1064 nm	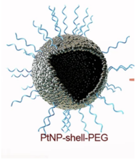	1	NA	73.7%	***Nat. Commun.*** 2024, 6362.
PtAg	1064	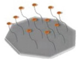	1	NA	45.7	***Adv Funct Mater***. 2021, 8(17): 2100386

4. Reference

[1] a) J.-D. Chai, M. Head-Gordon, *Phys. Chem. Chem. Phys.* **2008**, *10*, 6615-6620; b) H. Sun, S. Zhang, C. Zhong, Z. Sun, *J Comput Chem.* **2016**, *37*, 684-693; c) B. Moore, H. Sun, N. Govind, K. Kowalski, J. Autschbach, *J. Chem. Theory. Comput.* **2015**, *11*, 3305-3320; d) E. Frisc, M. Frisch, F. Clemente, G. Trucks, *Inc., Wallingford CT.* **2013**.

[2] a) M. J. Abraham, T. Murtola, R. Schulz, S. Páll, J. C. Smith, B. Hess, E. Lindahl, *SoftwareX.* **2015**, *1*, 19-25; b) C. Zhu, *Time-dependent Density Functional Theory: Nonadiabatic Molecular Dynamics*, CRC Press, **2022**.

[3] a) S. Diao, G. Hong, J. T. Robinson, L. Jiao, A. L. Antaris, J. Z. Wu, C. L. Choi, H. Dai, *J Am Chem Soc.* **2012**, *134*, 16971-16974; b) Q. Yang, Z. Ma, H. Wang, B. Zhou, S. Zhu, Y. Zhong, J. Wang, H. Wan, A. Antaris, R. Ma, X. Zhang, J. Yang, X. Zhang, H. Sun, W. Liu, Y. Liang, H. Dai, *Adv Mater.* **2017**, *29*, 1605497.

[4] W. E. Meador, E. Y. Lin, I. Lim, H. C. Friedman, D. Ndaleh, A. K. Shaik, N. I. Hammer, B. Yang, J. R. Caram, E. M. Sletten, *Nat. chem.* **2024**, *16*, 970-978.

[5] G.-l. Wu, B. Sun, Y. He, X. Tan, Q. Pan, S. Yang, N. Li, M. Wang, P. Wu, F. Liu, H. Xiao, L. Tang, S. Zhu, Q. Yang, *Chem Eng J.* **2023**, *463*, 142372.

[6] H. Wan, J. Yue, S. Zhu, T. Uno, X. Zhang, Q. Yang, K. Yu, G. Hong, J. Wang, L. Li, Z. Ma, H. Gao, Y. Zhong, J. Su, A. L. Antaris, Y. Xia, J. Luo, Y. Liang, H. Dai, *Nat. Commun.* **2018**, *9*, 1171

[7] P. Chen, F. Qu, S. Chen, J. Li, Q. Shen, P. Sun, Q. Fan, *Adv. Fun. Mater.* **2022**, *32*, 2208463.

[8] a) Q. Yang, Z. Hu, S. Zhu, R. Ma, H. Ma, Z. Ma, H. Wan, T. Zhu, Z. Jiang, W. Liu, *J. Am. Chem. Soc.* **2018**, *140*, 1715-1724; b) X. Zhang, H. Wang, A. L. Antaris, L. Li, S. Diao, R. Ma, A. Nguyen, G. Hong, Z. Ma, J. Wang, *Adv. Mater.* **2016**, *28*, 6872; c) R. Tian, H. Ma, Q. Yang, H. Wan, S. Zhu, S. Chandra, H. Sun, D. O. Kiesewetter, G. Niu, Y. Liang, *Chem. Sci.* **2019**, *10*, 326-332; d) H. Wan, H. Ma, S. Zhu, F. Wang, Y. Tian, R. Ma, Q. Yang, Z. Hu, T. Zhu, W. Wang, *Adv. Fun. Mater.* **2018**, *28*, 1804956; e) A. He, F. Xia, D. Han, Q. Yang, W. Tan, *Sci. China. Chem.* **2024**, *67*, 2767-2774.
